# ACSL5 Regulates Glucose Metabolism and Chemotherapy Sensitivity in Colorectal Cancer Cells under Glutamine Deficiency

**DOI:** 10.1002/advs.202510801

**Published:** 2025-12-08

**Authors:** Shuai Tian, Qiaoxia Zhang, Xuedan Sun, Rick Francis Thorne, Zeyuan Shi, Qiang Ji, Zhangran Sun, Yuanxiang Lu, Qun Zhao, Xianjun Yu, Wanglai Hu, Mian Wu

**Affiliations:** ^1^ Translational Research Institute of Peoples Hospital of Zhengzhou University and Academy of Medical Science Tianjian Laboratory of Advanced Biomedical Sciences Zhengzhou University Henan International Joint Laboratory of Non‐coding RNA and Metabolism in Cancer Zhengzhou 450003 China; ^2^ Department of Hepatobiliary Surgery Centre for Leading Medicine and Advanced Technologies of IHM The First Affiliated Hospital of USTC Division of Life Sciences and Medicine University of Science and Technology of China Hefei 230031 China; ^3^ Department of Pharmacology School of Basic Medical Sciences Academy of Medical Science Zhengzhou University Zhengzhou 450000 China; ^4^ Department of Breast Surgery Zhengzhou University People Hospital & Henan Provincial People's Hospital Zhengzhou 450003 China; ^5^ School of Basic Medical Sciences Hubei Key Laboratory of Embryonic Stem Cell Research Biomedical Research Institute Hubei University of Medicine Shiyan 442000 China; ^6^ Academy of Medical Science Tianjian Laboratory of Advanced Biomedical Sciences State Key Laboratory of Metabolic Dysregulation & Prevention and Treatment of Esophageal Cancer Zhengzhou University Translational Research Institute People's Hospital of Zhengzhou University Henan International Joint Laboratory of Non‐coding RNA and Metabolism in Cancer Zhengzhou 450003 China

**Keywords:** ACSL5, chemotherapy sensitivity, DNA damage, glucose metabolism, glutamine deficiency, p53

## Abstract

Glutamine metabolism is crucial for sustaining tumor cell viability and growth, broadly promoting prospects for the therapeutic targeting of glutamine dependence. However, further research is needed to address key translational issues, particularly to better understand the adaptive survival responses employed by cancer cells in overcoming nutrient deficiency. Long‐chain acyl‐CoA synthetase 5 (ACSL5) is found to be upregulated under glutamine deprivation, acting to sustain tumor cell viability by enhancing both glycolytic flux and oxidative phosphorylation. ACSL5 operates within a p53 regulatory loop: p53 transcriptionally upregulates ACSL5, while ACSL5 competes with MIB1 to stabilize MDM2, suppressing p53 expression. Mechanistically, ACSL5 relieves p53‐mediated inhibition of PGAM1 to drive glycolysis, while its mitochondrial localization promotes IDH2 activation to accelerate the TCA cycle. Nonetheless, these metabolic increases also generate reactive oxygen species (ROS), inducing DNA damage and significantly enhancing colorectal cancer cell sensitivity to oxaliplatin. The latter provides an explanation as to why colorectal tumors with high ACSL5 expression display preferentially improved patient outcomes from chemotherapy. Collectively, the findings reveal a new pathway for non‐genetic chemotherapy resistance mechanisms, deepen the understanding of metabolic reprogramming in tumor cells, and offer potential therapeutic targets for future treatment strategies.

## Graphical abstract

Glutamine deprivation triggers ACSL5 upregulation in tumor cells, sustaining their viability via dual metabolic rewiring programs. ACSL5 enhances glycolysis by relieving p53's inhibition of PGAM1 while also sustaining mitochondrial respiration and TCA cycle flux through promoting IDH2 dimerization. This comes at a cost, elevated ROS and weakened p53 dependent DNA repair mechanisms sensitize tumor cells to oxaliplatin chemotherapy.

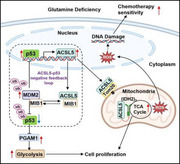

## 1 Introduction

Colorectal cancer ranks as the third most commonly diagnosed cancer globally, with its incidence rate continuing to rise in emerging economies.^[^ ^1^ ^]^ The increasing number of early‐onset colorectal cancer (EOCRC) cases, particularly among patients under 50, has become a significant global health challenge.^[^ ^2^ ^]^ Over the past decade, substantial progress has been made in understanding the biological mechanisms underpinning colorectal cancer development and progression, improving treatment strategies, and enhancing overall survival rates. However, the field still faces critical challenges, including optimizing treatment protocols and advancing precision medicine approaches, largely facilitated by further in‐depth research to identify key molecular biomarkers and treatment targets.^[^ ^1^ ^]^

Metabolic reprogramming is a hallmark of cancer cells, enabling their growth and survival within the hostile tumor microenvironment. The study of cancer metabolism dates back to the early 20th century when Otto Warburg discovered that cancer cells preferentially produce energy through glycolysis, even in the presence of sufficient oxygen—a phenomenon now known as the “Warburg effect”.^[^ ^3^ ^]^ Continuing research over subsequent decades has deepened our understanding of metabolic reprogramming, revealing a complex network of pathway alterations that collectively drive tumor development and cancer progression.^[^ ^4^ ^]^ Contemporary findings show that metabolic reprogramming extends beyond effects on glycolysis, reflecting a spectrum of adaptive metabolic changes that collectively support the rapid proliferation and survival of tumor cells. In particular, glutamine metabolism provides an essential source of carbon and nitrogen to support the synthesis of proteins, lipids, nucleotides, and amino acids, as well as its downstream metabolites, activating key signaling pathways such as mTOR, NF‐κB, and HIF‐1α.^[^ ^5^, ^6^, ^7^ ^]^ Moreover, glutamine also serves as a precursor for glutamate, contributing to TCA cycle replenishment via its conversion to α‐ketoglutarate while also contributing to the production of glutathione (GSH) toward maintaining redox homeostasis.^[^ ^8^, ^9^ ^]^ Notably, glutamine is one of the most consumed amino acids by cancer cells, with conditions of metabolic stress or oncogenic activation triggering increased glutamine uptake and utilization, a dependency often referred to as “glutamine addiction”.^[^ ^10^ ^]^

Given the central role of glutamine in tumor biology, glutamine metabolism has been prioritized as a potential therapeutic target for precision medicine. The main strategies considered have included inhibiting glutaminase (GLS) or blocking glutamine transport proteins, although single‐agent studies have often proven insufficient to completely suppress tumor growth. However, more promising findings have been obtained by combining metabolic inhibitors with immunotherapy. For example, the glutaminase inhibitor CB‐839 (Telaglenastat) significantly enhances the cytotoxicity of tumor‐infiltrating lymphocytes (TIL) against melanoma cells and improves the anti‐tumor activity of anti‐PD‐1 or anti‐CTLA‐4 antibodies in mouse models, showing favorable anti‐tumor effects in various solid and hematological tumors.^[^ ^11^, ^12^ ^]^ Additionally, the glutamine‐blocking drug JHU083 induces tumor cell death and reprograms immunosuppressive macrophages into immune‐enhancing macrophages, activating the body's anti‐tumor immune response.^[^ ^13^ ^]^ Other studies have shown that combining glutamine metabolism inhibitors with conventional chemotherapeutic drugs can further enhance efficacy. For example, combining GLS inhibitors CB‐839 with 5‐fluorouracil (5‐FU).^[^ ^14^ ^]^ Moreover, targeting glutamine metabolism may improve the tumor microenvironment and enhance the anti‐tumor activity of immune cells.^[^ ^15^, ^16^ ^]^ Nonetheless, while these findings have revealed the potential of targeting glutamine metabolism, there remains an implicit need to overcome current therapeutic inefficiencies. Indeed, as highlighted by our previous studies, the underlying resistance mechanisms of tumor cells to glutamine deprivation are complex and diverse.^[^ ^17^, ^18^, ^19^, ^20^ ^]^

The metabolic plasticity of tumor cells presents a major challenge for clinical translation. This adaptability is driven by the interdependence of glucose and glutamine metabolism—pathways that jointly sustain cellular energy production and supply essential metabolic intermediates.^[^ ^21^, ^22^ ^]^ These pathways not only promote tumor cell proliferation and invasion via activation of key signaling networks but also enable dynamic adaptation to microenvironmental stress.^[^ ^23^ ^]^ For example, inhibition of glycolysis triggers compensatory glutamine uptake to maintain TCA cycle flux and energy supply, while glutamine scarcity shifts cells toward glycolytic dependency to sustain tumor cell survival.^[^ ^20^ ^]^ Glutamine further acts as a metabolic nexus, coordinating lipid metabolism by supplying citrate for fatty acid synthesis and NADPH for fatty acid oxidation (FAO), thereby dynamically balancing anabolic and catabolic demands. While therapeutic co‐targeting of glucose and glutamine pathways could disrupt this adaptive resilience, the lack of robust biomarkers to identify tumors reliant on these dual metabolic networks remains a critical barrier to precision intervention.

Seeking to provide new insights into tumor cell survival responses under limiting glutamine conditions, our screen highlighted that ACSL5 (long‐chain Acyl‐CoA Synthetase 5) was commonly upregulated in carcinoma cell lines of mixed origin. Previous reports have indicated that ACSL5 is regulated by multiple factors, including nutritional status, hormones (such as insulin), and environmental stress.^[^ ^24^ ^]^ ACSL5 is primarily known as a key enzyme involved in fatty acid synthesis, catalyzing the conjugation of long‐chain fatty acids with coenzyme A (CoA) to produce activated acyl‐CoA. Contradictory roles for ACSL5 have currently been reported in cancer. For instance, ACSL5 has been shown to promote tumor proliferation and metastasis in colorectal and gastric cancers, whereas its expression is associated with a favorable prognosis in ER‐negative breast cancers.^[^ ^25^, ^26^, ^27^ ^]^ Here, our investigations involving colorectal cancer provide a basis for reconciling such contrasting findings.

We found that the induction of ACSL5 under glutamine‐deficient conditions functions as a compensatory mechanism to support tumor cell survival and proliferation. This benefit was realized from dual effects on mitochondrial respiration and glycolysis, actions arising from discrete cellular pools of ACSL5. Nevertheless, the heightened metabolic activities of ACSL5 sensitizing cancer cells to chemotherapy drugs by promoting ROS production and accelerating DNA damage. Notably, colorectal cancer patients whose tumors expressed higher ACSL5 levels were shown to benefit from chemotherapy, proposing a rationale as to why ACSL5 is prognostic for improved outcomes. These findings elucidate ACSL5's complex role in cancer metabolism and establish its potential as a predictive biomarker for chemotherapy response. They also provide a rationale for future therapeutic strategies simultaneously targeting both glycolysis and mitochondrial respiration in cancer cells.

## 2 Results

### 2.1 ACSL5 is Upregulated and Translocates to Mitochondria under Glutamine Deficiency

Since many cancer cells heavily rely on glutamine, we hypothesized that adaptive mechanisms relevant to the targeting of glutamine metabolism would be revealed in cells subjected to glutamine deficiency conditions. To this end, we conducted proteomic screening on human hepatocellular carcinoma HepG2 cells cultured in either glutamine‐replete or depleted medium. From the list of differentially expressed proteins (Table S1, Supporting Information), we prioritized cell metabolism‐related candidates to create a shortlist of 12 differentially expressed proteins. This included 6 upregulated (IGFBP1, ARL5B, FTO, SLC19A2, ACSL5, and PUM2) and 6 downregulated proteins (PEBP1, FADS2, DUSP9, FKBP1A, GLB1, and GCDH) (Figure S1A, Supporting Information). As further refinement, we used Western blotting to examine the levels of each protein candidate in five carcinoma cell lines of diverse tissue origins (HepG2, HCT116, A549, KYSE‐450, and 786‐O). These results provided good concordance with the HepG2 cell screening data, although only ACSL5 was consistently upregulated in all five cell lines in response to glutamine depletion (Figure S1B, Supporting Information). Accordingly, we chose to focus our experiments on ACSL5 with its remarkable increase in HCT116 cells, suggesting this as the ideal cell line model for investigating the underlying mechanisms.

**Figure 1:**
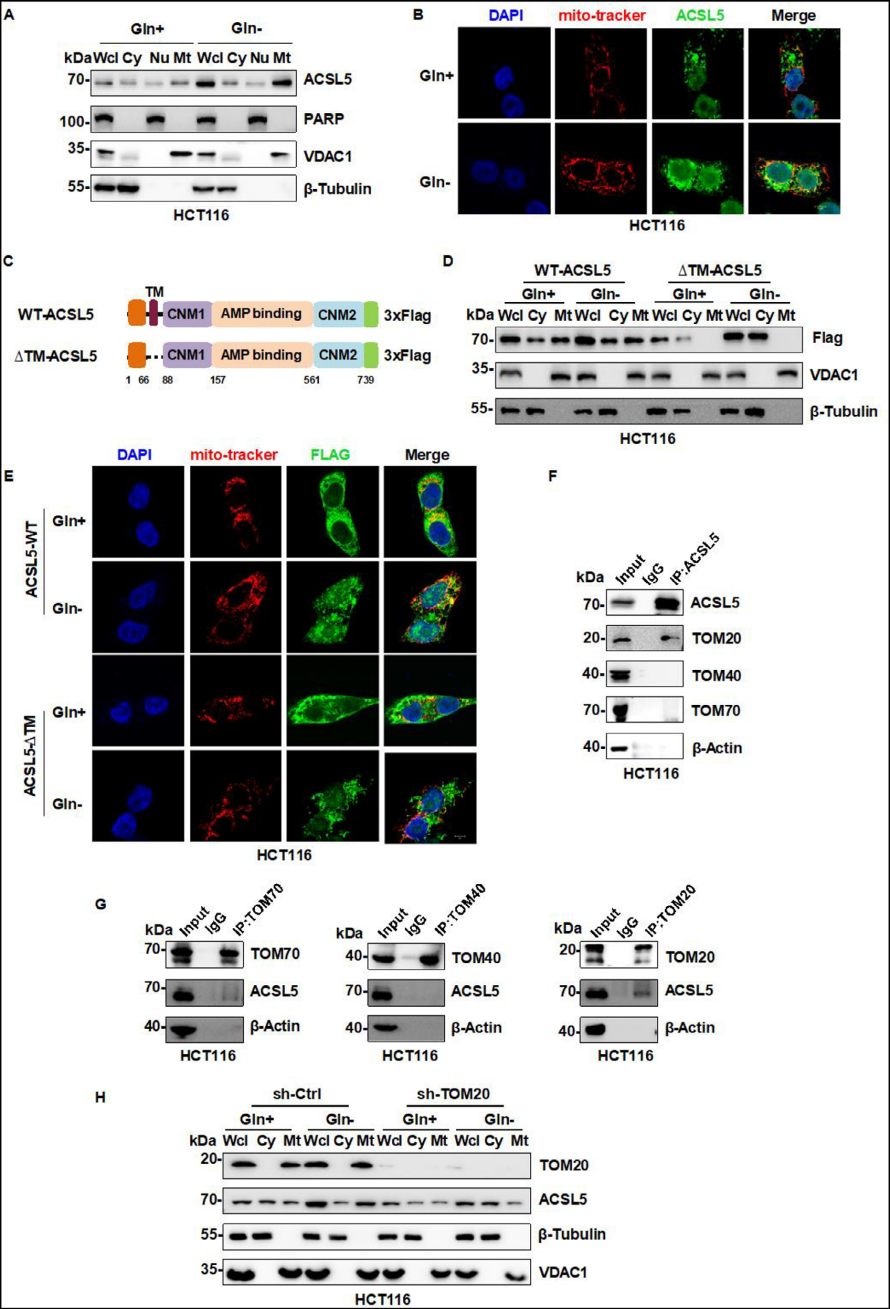
ACSL5 is upregulated and translocates to mitochondria under glutamine deficiency. A) Western blot analysis of ACSL5 protein levels in whole cell lysates (Wcl) or subcellular fractions, including cytosol (Cy), nucleus (Nu), and mitochondria (Mt) in HCT116 cells subjected to control (4 mm glutamine) or glutamine deficiency conditions for 48 h. The fidelity of the nuclear, mitochondrial, and cytosolic fractions was verified by detection of PARP, VDAC1, and β‐Tubulin, respectively. B) Representative confocal images of HCT116 cells cultured with or without glutamine showing immunofluorescence staining against ACSL5 (green) in concert with MitoTracker (red) and DAPI staining to decorate mitochondria and nuclei, respectively. Scale bars, 5 µm. C) Diagrammatic representation of Flag‐tagged expression constructs showing wild type (WT‐ACSL5) or mutant ACSL5 (ΔTM‐ACSL5) with the potential mitochondria transmembrane domain removed. D) HCT116 cells overexpressing wild‐type ACSL5 (WT‐ACSL5) or mutant ACSL5 (ΔTM‐ACSL5) constructs were cultured with or without glutamine for 48 h before Western blotting analysis of ACSL5 in the indicated subcellular fractions (fraction fidelity demonstrated as per (A)). E) Representative confocal images of WT‐ACSL5 and ΔTM‐ACSL5 expressing HCT116 cells with or without glutamine deficiency conditions for 48 h. Scale bars, 5 µm. F, G) Co‐immunoprecipitation analyses conducted in HCT116 cells between ACSL5, TOM20, TOM40, and TOM70 (F) or individually between TOM70, TOM40 and TOM20 and ACSL5 (G). β‐Actin served as a negative control. H) HCT116 cells were transduced with an shRNA control (shCtrl) or shTOM20 construct were cultured with or without glutamine for 48 h before Western blot analysis of ACSL5 in whole cell lysates or the indicated subcellular fractions (fraction fidelity demonstrated as per (A)). (A, B, D–H) represent three independent experiments.

Prior studies indicate that ACSL5 is localized to the mitochondrial outer membrane, but like other ACSL proteins, it may occupy different cellular locations such as the endoplasmic reticulum.^[^ ^28^ ^]^ To clarify the cellular location of ACSL5, particularly its increased levels following glutamine restriction, we performed cell fractionation and immunofluorescence experiments on HCT116 cells cultured with or without glutamine. The results showed that glutamine restriction largely supplemented the mitochondrial pool of ACSL5 protein with other staining of mitochondrial‐adjacent organelles (**Figure** **1**A,B). Consistently, treating cells with the glutaminase inhibitor CB‐839 phenocopied the results of glutamine deprivation with notable increases in ACSL5 mitochondrial levels (Figure S1C,D, Supporting Information).

The precise details of how ACSL5 specifically targets mitochondria are not fully clear, although it reportedly carries a mitochondrial transmembrane sequence (residues 66‐88).^[^ ^24^ ^]^ To glean further information regarding the mitochondrial targeting of ACSL5, we constructed the ΔTM‐ACSL5 mutant lacking residues 66‐88. Indeed, after ectopically expressing the ΔTM‐ACSL5, we found that the ΔTM‐ACSL5 mutant failed to target mitochondria under both glutamine‐deficient and replete culture conditions (Figure 1C–E), indicative of the essential role of the TM sequence. To further assess the sub‐mitochondrial localization of ACSL5, we performed proteinase K protection assays on isolated mitochondria. In comparison to the outer mitochondrial membrane (OMM) proteins TOM20 and TOM70, ACSL5 appeared partially resistant to proteinase K digestion, while the inner mitochondrial membrane (IMM) COXIV and ATP5A1 were resistant unless digestions were performed in the presence of Triton‐X‐100 (Figure S1E,F, Supporting Information). Additional co‐immunoprecipitation (CO‐IP) assays to determine if ACSL5 interacted with OMM receptors showed that reciprocal interactions occur between ACSL5 and TOM20 but not with TOM40 or TOM70 (Figure 1F,G). Instructively, knockdown of TOM20 prevented the increase in the mitochondrial pool of ACSL5 under glutamine‐deficient conditions (Figure 1H), whereas the knockdown of either TOM40 or TOM70 elicited no such effects (Figure S1G,H, Supporting Information).

Collectively, these results indicate that ACSL5 induction may be broadly relevant to the responses of diverse‐origin carcinoma cells to glutamine deficiency. Furthermore, this process supplements both cytoplasmic and mitochondrial pools of ACSL5, the latter resulting from ACSL5 targeting mitochondria via direct interactions with TOM20.

### 2.2 ACSL5 Promotes the Proliferation of Colorectal Cancer Cells

**Figure 2:**
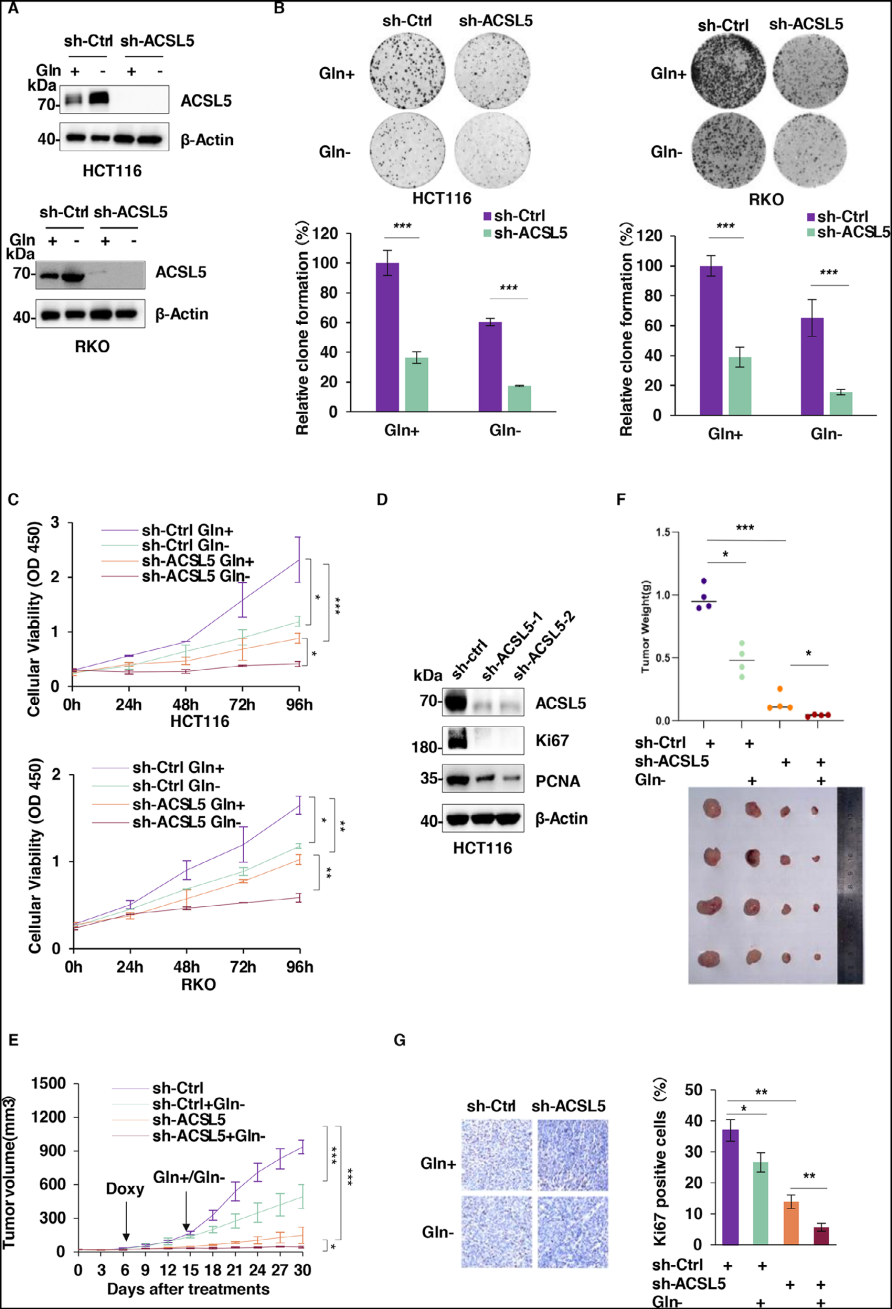
ACSL5 promotes the proliferation of colorectal cancer cells. A) Western blot analysis of ACSL5 in whole cell lysates after transducing HCT116 (top) or RKO cells (bottom) with control (sh‐Ctrl) or ACSL5‐targeting (sh‐ACSL5) lentiviral constructs in combination with or without glutamine deficiency conditions for 48 h. β‐Actin was used as a loading control throughout. B) Colony formation assays were conducted on the cells from (A) in combination with or without glutamine deprivation. Representative well images (top) and quantitation of relative growth using % area measurements (bottom). C) CCK‐8 assays were conducted on the cells from (A) in combination with or without glutamine deprivation. D) Western blot analysis of ACSL5, Ki67, PCNA, and β‐Actin loading control levels in HCT116 cells after transduction with shCtrl or two independent shRNAs targeting ACSL5. E–G) HCT116 cells expressing sh‐ctrl versus sh‐ACSL5 were inoculated into the flanks of nude mice. The establishment of xenografted tumors was monitored every 3 days by caliper‐estimated volume measurements (E). Comparison of final tumor weights in individual mice (F) together with determination of mitotic rates in the tumors using immunohistochemical (IHC) staining against Ki67 (G). Representative IHC images (left; Scale bars, 20 µm) and proliferative indices determined from the relative numbers of Ki67+ cells (right). (A‐D) represent three or more independent experiments. (B,C, E, G) Data are mean ± SD: (B,C,E) two‐way ANOVA with Tukey's multiple comparisons test; (G) two‐tailed multiple *t* test. ^*^ *P* < 0.05, ^**^ *P* < 0.01, ^***^ *P* < 0.001.

To explore the relevance of ACSL5 to primary cancer cell phenotypes, we employed shRNA‐mediated knockdown of ACSL5 in the HCT116 and RKO colorectal cancer cell lines. Clonogenicity assays together with CCK‐8 assays showed that depleting ACSL5 diminished both the clone‐forming ability and growth of both cell lines under normal and glutamine‐deficient conditions, with the inhibitory effects of ACSL5 knockdown being accentuated in the absence of glutamine (**Figure** **2**A–C). Consistent with an antiproliferative effect, knockdown of ACSL5 resulted in significant decreases in the expression of the proliferation‐related markers Ki67 and PCNA (Figure 2D). To ensure these findings were reflected in vivo, we conducted xenograft experiments in nude mice maintained with control or glutamine‐deficient chow, the latter a feeding regime which results in an approximate 40–60% reduction in circulating glutamine levels.^[^ ^17^, ^29^ ^]^ Under both regular and glutamine‐reduced conditions, ACSL5 knockdown cells formed significantly smaller tumors compared to control cells, with the excised tumors showing reduced Ki67 positivity, reflecting lower rates of proliferation (Figure 2E–G).

Together, these results indicate that ACSL5 promotes the proliferation of tumor cells both in vitro and in vivo, likely providing survival advantages for cancer cells in an energy‐restricted environment characterized by glutamine deficiency.

### 2.3 p53 Transcriptionally Upregulates ACSL5 During Glutamine Deficiency

**Figure 3:**
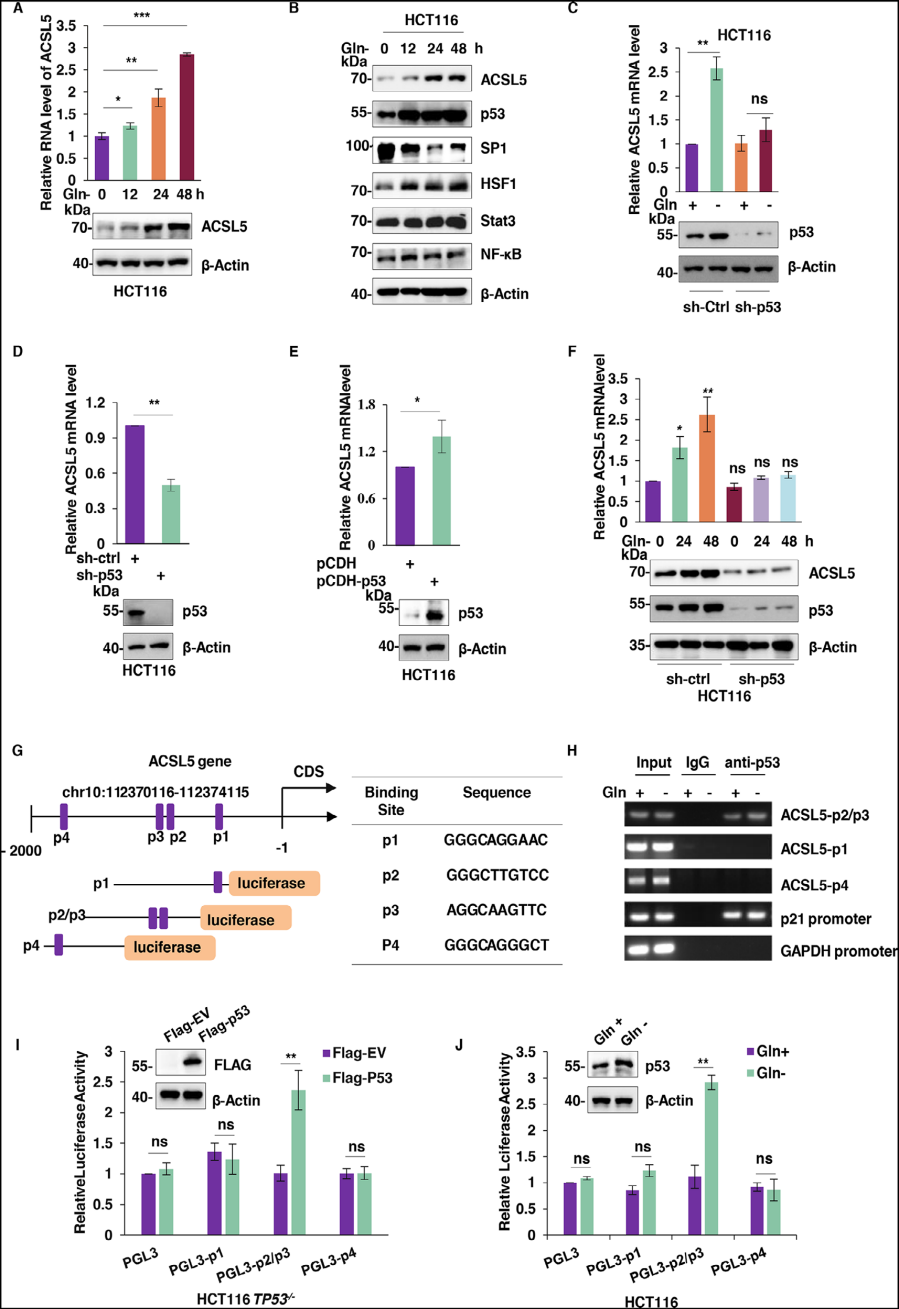
p53 transcriptionally upregulates ACSL5 during glutamine deficiency. A) Analysis of ACSL5 expression in HCT116 cells using qPCR (top) and Western blot (bottom) following removal of glutamine from the culture medium for the indicated times. β‐Actin served as a housekeeping gene and loading control, respectively, throughout the experiments. B) Western blot analysis of transcription factor expression (p53, SP1, HSF1, Stat3, NF‐κB) in ACSL5 protein levels in HCT116 cells subjected to control or glutamine deficiency conditions for 48 h. C) Analysis of p53 expression in HCT116 cells subjected to control shRNA and p53 knockdown (sh‐p53) in combination with or without glutamine removal using qPCR (top) and Western blot (bottom). D, E) The levels of ACSL5 mRNA (top) were determined by qPCR in HCT116 cells relative to controls after p53 knockdown (D) or overexpression using the pCDH‐p53 construct (E). Changes in p53 expression were verified using Western blotting (bottom). F) The relative levels of ACSL5 mRNA were determined by qPCR in control and p53 knockdown HCT116 cells in combination with or without glutamine deprivation (top). Changes in p53 expression were verified using Western blotting (bottom). G) Schematic depicting the location of putative p53 binding sites (p1, p2, p3, and p4) in the sense strand of the ACSL5 promoter determined from the JASPAR2020 and UCSC databases together with the design of overlapping pGL3‐based luciferase reporter constructs incorporating sites p1, p2/p3, and p4 (left). The predicted p53 binding sequences are listed (right). H) ChIP assays conducted in HCT116 cells cultured with or without glutamine for 48 h against p53 antibodies or control IgG. Semi‐quantitative PCR was used to detect recovered p1, p2/p3, and p4 fragments with amplicons targeting the p21 and GAPDH promoters serving as positive and negative controls, respectively. I) Dual luciferase reporter assays were conducted using the pGL3 reporters in (G) in p53 null (*TP53* ^−/−^) HCT116 cells without (Flag‐EV) or with ectopic expression of Flag‐p53. J) Dual luciferase reporter assays were conducted using the pGL3 reporters in (G) in wildtype HCT116 cells without or with glutamine deprivation for 48 h. (A‐F, H‐J) represent three independent experiments. (A, C, D‐F, I, J) Data are mean ± SD; (A, C, F) two‐tailed multiple *t* test; (D, E) two‐tailed unpaired *t* test; (I, J) two‐way ANOVA with Tukey's multiple comparisons test. ^*^ *P* < 0.05, ^**^ *P* < 0.01, ^***^ *P* < 0.001.

We next considered the basis for the increase in ACSL5 expression following glutamine deprivation. Removal of glutamine resulted in time‐dependent increases in ACSL5 mRNA levels with correlative changes in ACSL5 protein expression (**Figure** **3**A), together suggesting that glutamine deficiency invoked increased ACSL5 transcription. To investigate how glutamine influences the upregulation of ACSL5 mRNA, we used the online tools JASPAR and PROMO5 to predict potential transcription factor binding motifs in the upstream promoter region of *ACSL5*. From this data, we prioritized SP1, HSF1, Stat3, NF‐κB, and p53 for further analysis (Figure S2A, Supporting Information). We first evaluated the impact of glutamine deprivation on their expression, observing increases in the levels of p53 and HSF1, decreases in SP1 levels, but no changes in Stat3 and NF‐κB expression (Figure 3B). Further knockdown experiments undertaken against each transcription factor showed that p53 depletion abolished the upregulation of ACSL5 mRNA in response to glutamine deprivation, whereas the knockdown of HSF1, SP1, Stat3, or NF‐κB did not affect the glutamine deprivation‐induced upregulation of ACSL5 mRNA (Figure 3C; Figure S2B–E, Supporting Information).

To further validate the role of p53 in ACSL5 transcription, we undertook knockdown and overexpression experiments to manipulate p53 levels. Consistently, the levels of ACSL5 mRNA were reduced when p53 was silenced, while its overexpression increased ACSL5 levels (Figure 3D,E). Moreover, p53 silencing completely prevented the upregulation of ACSL5 mRNA in response to glutamine deprivation (Figure 3F). To verify that p53 was directly responsible for ACSL5 transactivation, we constructed luciferase reporters based on the upstream promoter region of *ACSL5*. For this, we created overlapping truncated sequences, each possessing one of the four potential p53 binding sites (p1‐p4), grouping together the p2 and p3 motifs given their close proximity (Figure 3G). ChIP assays targeting the different p53 binding sites in ACSL5 recorded specific interactions between p53 and p2/p3 site, which were relatively increased following glutamine deprivation (Figure 3H). Dual‐luciferase reporter assays conducted in TP53 null HCT116 cells showed that ectopic expression of p53 significantly increased the reporter activity of p2/p3 but not other constructs (Figure 3I). Similarly, glutamine deficiency, which also induces p53 expression, was shown to only increase the activity of the p2/p3 reporter (Figure 3J). Lastly, we compared the glutamine deprivation response of cell lines with different p53 genetic backgrounds, including the wild‐type HCT116 and RKO cell lines, p53 knockout (*TP53* ^−/−^) HCT116 cells, and the HT29 p53 mutant cell line. We found that ACSL5 mRNA was only upregulated in response to glutamine deprivation in p53 wild‐type cell lines, whereas no such response was observed in p53 knockout or p53 mutant cell lines (Figure S2F–I, Supporting Information).

Collectively, these results show that p53 is substantially responsible for the transactivation of ACSL5 in cells exposed to glutamine restriction, acting directly at its promoter through binding to the p2/p3 region.

### 2.4 ACSL5 Competes with MDM2 for Binding MIB1 to Regulate p53

**Figure 4:**
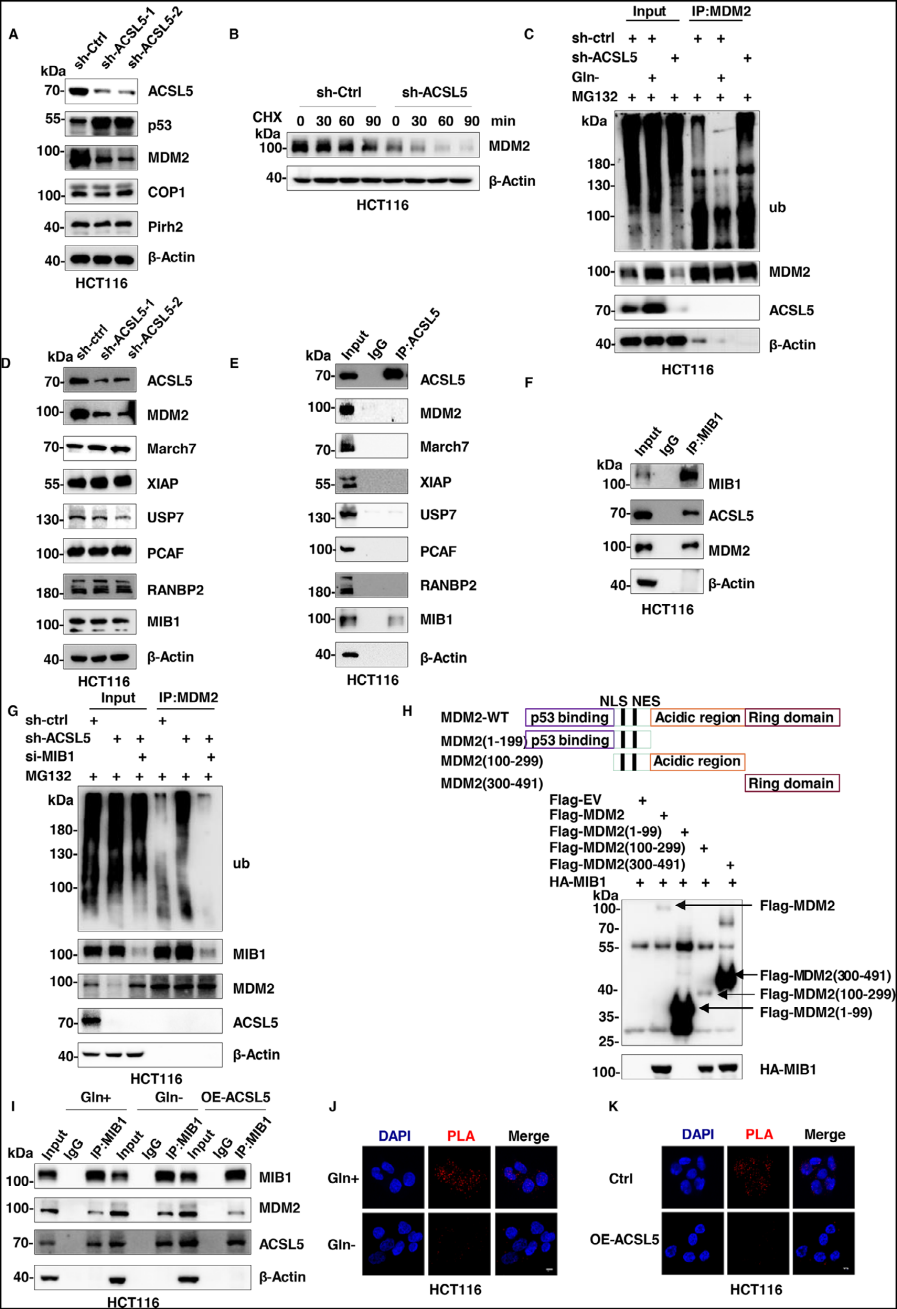
ACSL5 competes with MDM2 for binding MIB1 to regulate p53. A) HCT116 cells were transduced with lentiviruses containing control (sh‐Ctrl) or independent shRNAs targeting ACSL5. Thereafter, the levels of ACSL5, p53, and the p53‐targeting ubiquitin ligases MDM2, COP1, and Pirh2, analyzed by Western blotting. β‐Actin was used throughout as a control. B) Cycloheximide chase assays conducted on HCT116 cells bearing sh‐ctrl or sh‐ACSL5 lentiviruses, comparing the levels and stability of MDM2 using Western blot. C) Ubiquitination assays against MDM2 were conducted in sh‐ctrl or sh‐ACSL5 HCT116 cells in the indicated combinations of glutamine deficiency followed by MG132 treatment. Input and MDM2 immunoprecipitants were subject to immunoblotting against ubiquitin, MDM2, and ACSL5 as indicated. D) HCT116 cells were transduced with lentiviruses containing control (sh‐Ctrl) or independent shRNAs targeting ACSL5. Thereafter, the levels of ACSL5, MDM2, and the MDM2‐targeting ubiquitin ligases March7, XIAP, USP7, PCAF, RANBP2, and MIB1 were analyzed by Western blotting. E) ACSL5 immunoprecipitants from HCT116 cells were subject to Western blotting to detect binding interactions between ACSL5, MDM2, or MDM2‐targeting ubiquitin ligases. F) MIB1 immunoprecipitants from HCT116 cells were subject to Western blotting to detect binding interactions between MIB1, ACSL5, and MDM2. G) MDM2 ubiquitination assays were conducted as per (C) in HCT116 cells, combining the shRNA‐mediated knockout of ACSL5 (sh‐ACSL5) with treatment with control siRNAs or against MIB1 (si‐MIB1) before MG132 treatment. Input and MDM2 immunoprecipitants were subject to immunoblotting against ubiquitin, MIB1, MDM2, and ACSL5 as indicated. H) Empty Flag‐vector (EV) or Flag‐tagged versions of wildtype (WT) or truncated MDM2 mutants were transfected in the indicated combinations along with HA‐MIB1 into 293T cells before lysis and immunoprecipitation with anti‐Flag antibodies. Samples were subject to Western blotting with anti‐Flag and HA antibodies to reveal co‐precipitating proteins. I) Co‐immunoprecipitation assays were conducted in control HCT116 cells cultured with or without glutamine for 48 h or after transfected OE‐ACSL5(pCDNA3.1‐ACSL5) to detect associations between MIB1, MDM2, and ACSL5. J, K) Proximity Ligation Assays (PLA) undertaken in HCT116 cells between ACSL5 and MIB1 in cells cultured with or without glutamine for 48 h (J) or in control versus OE‐ACSL5(pCDNA3.1‐ACSL5) transfected cells (K). Representative images collected using confocal microscopy using DAPI to decorate cell nuclei. (A‐K) represent three independent experiments.

While p53 was shown to be responsible for the increases in ACSL5 expression, we incidentally found that ACSL5 knockdown resulted in increased p53 protein levels, suggesting a form of reciprocal regulation. This mechanism was not transcriptional in nature since p53 mRNA levels remained unchanged following ACSL5 knockdown (Figure S3A, Supporting Information). In concert, we surveyed the expression of various E3 ubiquitin ligases known to target p53 for proteasomal destruction, finding that the protein levels of MDM2 but not COP1 or Pirh2 were decreased following knockdown of ACSL5 (**Figure** **4**A). Moreover, polyubiquitination assays showed that ACSL5 overexpression enhanced p53 ubiquitination; however, this effect was abolished upon MDM2 depletion (Figure S3B, Supporting Information). Notably, ACSL5 did not alter MDM2 mRNA levels (Figure S3C, Supporting Information). Further supporting the notion that ACSL5 may inhibit p53 protein stability by upregulating MDM2, we found that ACSL5 knockdown decreased MDM2 protein stability in association with increases in its polyubiquitination (Figure 4B,C). Additionally, glutamine deprivation markedly decreased MDM2 ubiquitination, together suggesting that ACSL5 regulates MDM2 stability by modulating its ubiquitination and proteasomal turnover. Moreover, since ACSL5 was not recovered with MDM2 immunoprecipitates, this suggested ACSL5 imparts such regulation in an indirect manner.

Previous studies have identified March7, XIAP, USP7, and PCAF as E3 ligases which specifically target MDM2.^[^ ^30^, ^31^, ^32^, ^33^ ^]^ Using mass spectrometry data to identify common interacting proteins, two E3 ubiquitin ligase candidates, namely RANBP2 and MIB1, were identified in the intersection between the ACSL5 and MDM2 interactomes^[^ ^30^ ^]^ (Figure S3D and Table S2, Supporting Information). Depleting or overexpressing ACSL5 did not alter the protein levels of RANBP2, MIB1, or other MDM2‐targeting E3 ligases (Figure 4D; Figure S3E, Supporting Information). Nevertheless, co‐immunoprecipitation experiments demonstrated that ACSL5 selectively bound to MIB1, with the notable finding that ACSL5, together with MDM2, was captured within MIB1 immunoprecipitates (Figure 4E,F). Additionally, we found that MIB1 knockdown increased MDM2 protein levels in association with enhanced protein stability (Figure S3F,G, Supporting Information). Instructively, the results of polyubiquitination assays showed that the increases in MDM2 ubiquitination following ACSL5 knockdown were effectively inhibited when MIB1 was simultaneously depleted (Figure 4G), proposing that ACSL5 binding modulates MIB1 activity and its targeting of MDM2. This likely represents a competitive mechanism since gradient overexpression of ACSL5 inhibited interactions between MIB1 and MDM2 in a dose‐dependent manner (Figure S3H, Supporting Information). Supporting this idea, mapping protein binding showed ACSL5 and MDM2 both interacted with MIB1 through its RING domain (Figure 4H; Figure S3I–K, Supporting Information). Moreover, as anticipated, interactions between MIB1 and ACSL5 were increased upon glutamine deprivation conditions with concordant decreases in interactions between MIB1 and MDM2, phenocopying the effects of ACSL5 overexpression (Figure 4I). Lastly, we found using proximity ligation assays (PLA) that glutamine deprivation, as well as ACSL5 overexpression, reduced interactions between MDM2 and MIB1 (Figure 4J,K).

Together, these findings indicate that ACSL5 competes with MDM2 for binding to MIB1, thereby reducing MIB1‐mediated ubiquitination and degradation of MDM2, with the ensuing stabilization of MDM2 promoting increased p53 degradation. This mechanism presumably acts as a negative feedback regulatory loop between ACSL5 and p53, ensuring their balanced expression is modulated following the glutamine deprivation response.

### 2.5 Upregulation of ACSL5 under Glutamine Deficiency Promotes Glycolysis through the p53‐PGAM1 Axis

**Figure 5:**
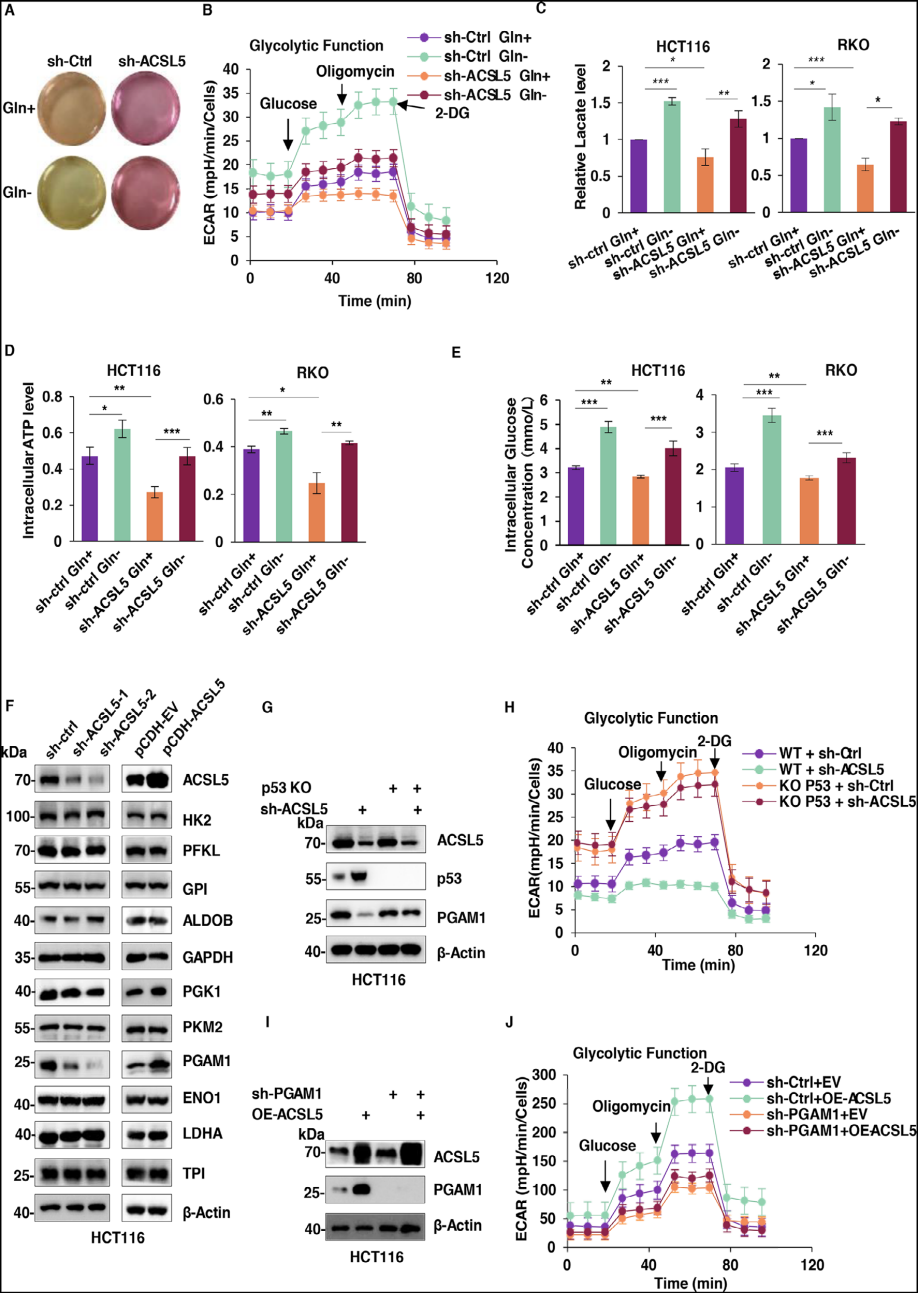
Upregulation of ACSL5 under glutamine deficiency promotes glycolysis through the p53‐PGAM1 axis. A) The degree of culture medium acidification compared in shCtrl and shACSL5 transduced HCT116 cells cultured with or without glutamine for 48 h. B) Cells from (A) were subjected to glycolysis stress tests using the Seahorse XF Analyzer. C–E) Levels of extracellular lactate production (C), intracellular ATP (D), and intracellular glucose concentrations (E) were compared in HCT116 (left) or RKO cells (right) after transduction with shCtrl or shACSL5 in combination with or without glutamine for 48 h. F) Western blot comparing the expression of core glycolytic enzymes in HCT116 cells following knockdown (left) or overexpression of ACSL5 (right). β‐Actin was used as a loading control throughout. G, H) WT and p53 null (KO) HCT116 cells were transduced with either sh‐ctrl or shACSL5 as indicated. Cells were analyzed by Western blot against ACSL5, p53, and PGAM1 (G) or subject to glycolysis stress tests (H). I, J) HCT116 cells were either transduced with shPGAM1 or transfected with pCDNA3.1‐ACSL5 or matched controls as indicated. Cells were analyzed by Western blot against ACSL5 and PGAM1 (I) or subject to glycolysis stress tests (J). (A‐J) represent three independent experiments. (B‐E, H, J) Data are mean ± SD: (C‐E) two‐way ANOVA with Tukey's test; ^*^ *P* < 0.05, ^**^ *P* < 0.01, ^***^ *P* < 0.001.

After establishing the mechanisms governing ACSL5 regulation during glutamine deficiency, we returned to consider how this ultimately serves to alter tumor cell metabolism and influence growth. Anaerobic glycolysis and aerobic mitochondrial respiration are the two main pathways by which generate cellular energy. Since tumor cells typically counteract restricted glutamine supplies through adaptive switching to glycolysis, we considered if ACSL5 was involved in regulating glycolysis, where, notably, p53 engages in glycolytic inhibition. To investigate this hypothesis, we first examined how glutamine deprivation along with ACSL5 knockdown affected proxy measures of glycolysis, including culture medium acidification, Seahorse XF assays measuring extracellular acidification rates (ECAR) together with lactate and ATP production. We found that subjecting cells to glutamine deprivation substantively enhanced glycolytic flux with concordant increases in lactate and ATP production levels, whereas ACSL5 silencing dampened glycolysis (**Figure** **5**A–D). Despite this, increases in glycolytic measures were still evident in ACSL5 knockdown cells following glutamine deprivation, indicating that ACSL5 is involved but not solely responsible for the glycolytic switch following glutamine restriction.

Glycolysis is highly dependent upon the activity of glycolytic enzymes as well as glucose availability, the latter regulated by glucose uptake. We observed that intracellular glucose concentrations increased under glutamine deprivation but decreased after ACSL5 knockdown, consistent with the changes in glycolysis levels observed in our preceding experiments (Figure 5E). We further explored the mechanism by which ACSL5 promotes glycolysis, examining protein expression changes in glycolytic cascade enzymes as well as LDHA in response to manipulating ACSL5 expression. Noticeable reductions were evident in phosphoglycerate mutase (PGAM1) following ACSL5 knockdown, while its overexpression increased PGAM1 levels (Figure 5F). Follow‐on experiments showed that the reductions in PGAM1 protein levels resulting from ACSL5 knockdown were dependent on p53, where notably, glycolytic regulation became uncoupled from the effects of ACSL5 silencing in p53‐knockout cells (Figure 5G,H). Moreover, consistent with the involvement of PGAM1 in ACSL5‐mediated glycolytic changes, we found that PGAM1 silencing completely negated the heightened glycolytic response arising after ACSL5 overexpression (Figure 5I,J). Thus, these findings establish that ACSL5 promotes glycolysis through regulating p53, which in turn modulates PGAM1 expression and glycolytic activity.

### 2.6 ACSL5 Promotes the TCA Cycle under Glutamine Deficiency

**Figure 6:**
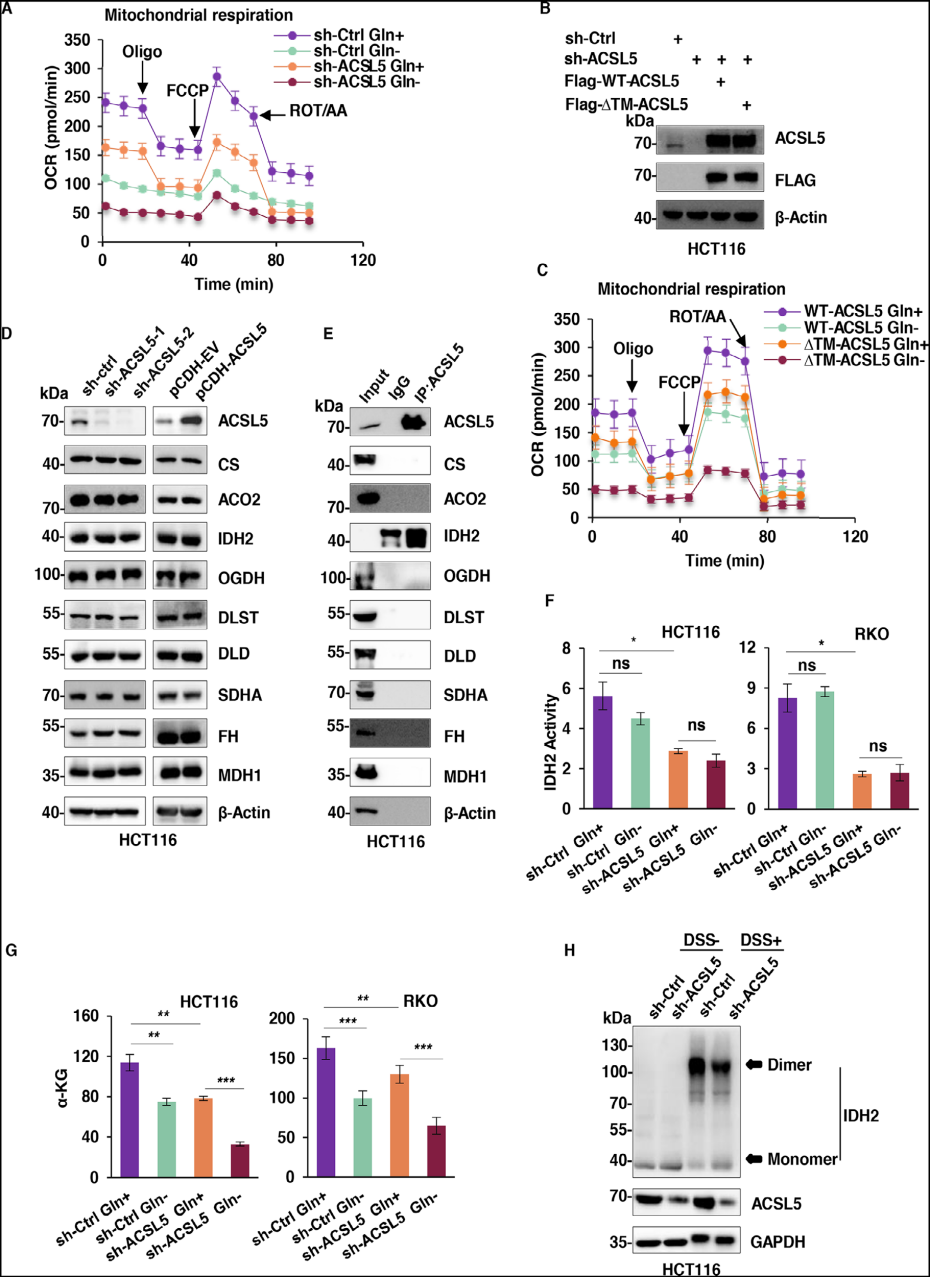
ACSL5 promotes the TCA Cycle under glutamine deficiency. A) Mitochondrial stress tests measuring oxygen consumption rates (OCR) were performed on HCT116 cells transduced with control (shCtrl) or shACSL5 lentiviruses using the Seahorse XF Analyzer in the presence and absence of exogenous glutamine. B, C) HCT116 cells were transduced with control (shCtrl) or shACSL5 lentiviruses with ACSL5 levels replenished by transfection with Flag‐tagged WT‐ACSL5 or ΔTM‐ACSL5 as indicated. The cells were analyzed by Western blotting using anti‐ACSL5 and anti‐Flag antibodies (B) or subjected to Mitochondrial stress tests (C). D) Western blot comparing the expression of core TCA cycle enzymes in HCT116 cells following knockdown (left) or overexpression of ACSL5 (right). β‐Actin was used as a loading control throughout. E) ACSL5 immunoprecipitants from HCT116 cells were subject to Western blotting to detect binding interactions between ACSL5 and core TCA cycle enzymes. F, G) IDH2 enzyme activity measurements (F) or α‐KG levels (G) in HCT116 (left) or RKO cells (right) following transduction with shctrl or shACSL5 lentiviruses in the presence and absence of glutamine. H) HCT116 cells transduced with shctrl or shACSL5 were treated without or with DSS crosslinker before subjecting whole cell lysates to Western blotting against IDH2, ACSL5, and GAPDH. (A‐H) represent three independent experiments. (A, C, F, G) Data are mean ± SD: (F, G) two‐way ANOVA with Tukey's test; ns, not significant, ^*^ *P* < 0.05, ^**^ *P* < 0.01, ^***^ *P* < 0.001.

In concert with the preceding analysis of glycolysis, we considered how the induction of ACSL5 impacts mitochondrial respiration. Indeed, the increased mitochondrial association of ACSL5 following glutamine withdrawal provided an implicit clue regarding the likely involvement of ACSL5 in mitochondrial respiration. Accordingly, we used Seahorse XF assays to measure the oxygen consumption rate (OCR) in control and ACSL5‐knockdown cells cultured under both glutamine‐replete and ‐deprived conditions. As expected, we found that glutamine restriction decreased OCR in control cells, while the knockdown of ACSL5 also dampened OCR, particularly during glutamine restriction, where OCR reductions were more pronounced (**Figure** **6**A). These results establish that ACSL5 is responsible for promoting mitochondrial respiration under both glutamine‐replete and depleted conditions, although not exclusively. We further took advantage of the ∆TM‐ACSL5 mutant construct developed in previous experiments to test the importance of ACSL5 localization to mitochondria. Using both wildtype and mutant ACSL5 constructs to reconstitute ACSL5‐knockdown cells, we found that OCR levels were accentuated in WT‐ACSL5 expressing cells compared to those bearing ∆TM‐ACSL5 under control conditions. Moreover, under glutamine deprivation, cells expressing ∆TM‐ACSL5 displayed highly diminished OCR rates relative to their WT‐ACSL5 counterparts (Figure 6B,C). These results suggest that ACSL5 localization to mitochondria functions to enhance mitochondrial respiration, supporting the notion of dual effects to not only promote glycolysis but also influence mitochondrial metabolism in tumor cells.

To glean additional clues, we examined the impact of ACSL5 knockdown or overexpression on the expression of key TCA cycle enzymes, although no significant changes in their levels were observed (Figure 6D). Alternatively, using immunoprecipitation to examine whether these enzymes could interact with ACSL5, we found that isocitrate dehydrogenase 2 (IDH2) was selectively recovered with ACSL5 (Figure 6E). Further protein domain mapping experiments were used to define the basis of the ACSL5‐IDH2 interaction, finding that the AMP domain of ACSL5 binds to the catalytic domain of IDH2 (Figure S4A,B, Supporting Information). Moreover, since the active form of IDH2 constitutes a dimer, we examined if ACSL5 influenced IDH2 dimerization. First, using alternative HA and GFP epitope‐tagged IDH2 constructs in combination with two‐phase immunoprecipitation experiments, we found that endogenous ACSL5 forms a ternary complex with dimerized IDH2 (Figure S4C, Supporting Information). Second, using DSS crosslinkers to stabilize endogenous IDH2 dimers, we found that ACSL5 knockdown resulted in lesser amounts of dimerized IDH2 and increased levels of monomeric IDH2, while ACSL5 overexpression enhanced IDH2 dimerization (Figure 6H; Figure S4D, Supporting Information). Consistently, IDH2 dimerization increased under glutamine‐deficient conditions (Figure S4E, Supporting Information). Together, these data demonstrate that ACSL5 directly binds to and stabilizes the active dimeric form of IDH2, notionally to promote its function.

IDH2 plays a key role in glutamine metabolism and mitochondrial respiration, converting isocitrate to α‐ketoglutarate (α‐KG) to enter the TCA cycle. Instructively, we found that ACSL5 knockdown reduced IDH2 enzymatic activity and reduced cellular α‐KG levels (Figure 6F,G). However, we noted that IDH2 activity did not change between glutamine‐replete and depleted conditions in these experiments. To verify that the mitochondrial respiratory defects caused by ACSL5 deletion (Figure 6A) were fundamentally due to the blockage of TCA cycle flux downstream of IDH2, we supplemented cells with α‐ketoglutarate (α‐KG) under glutamine starvation. The results clearly showed that α‐KG treatment significantly rescued OCR impairment (Figure S4F, Supporting Information). Together, these data support the notion that ACSL5 enhances mitochondrial respiration, modulating IDH2 activity to replenish α‐KG levels and maintain TCA cycle flux.

Given the direct role of ACSL5 in providing fuel for fatty acid β‐oxidation, the intriguing conclusion that ACSL5 enhances the TCA cycle via effects on IDH2 enzyme activity provides a conundrum as to which of these mechanisms is more important upon ACSL5 overexpression. To address this question, we employed the specific β‐oxidation inhibitor Etomoxir to block the fatty acid oxidation pathway in combination with manipulation of ACSL5, using Seahorse XF assays to monitor changes in mitochondrial respiration. As expected, Etomoxir treatment substantially deflated cellular OCR rates in control and ACSL5 overexpressing cells, although the OCR rates in Etomoxir‐treated ACSL5 overexpressing cells remained significantly higher than untreated control cells (Figure S4G, Supporting Information). This finding suggests that inhibiting β‐oxidation only partially counteracts the increase in mitochondrial respiration resulting from ACSL5 overexpression, supporting the idea that ACSL5 has a dual regulatory role in mitochondrial metabolism – namely, it not only increases the supply of acetyl‐CoA through the classical β‐oxidation pathway but also optimizes the efficiency of the TCA cycle by enhancing the dimerization and activity of the IDH2 enzyme.

### 2.7 ACSL5 Promotes ROS Production, Induces DNA Damage, and Increases Sensitivity to Oxaliplatin

**Figure 7:**
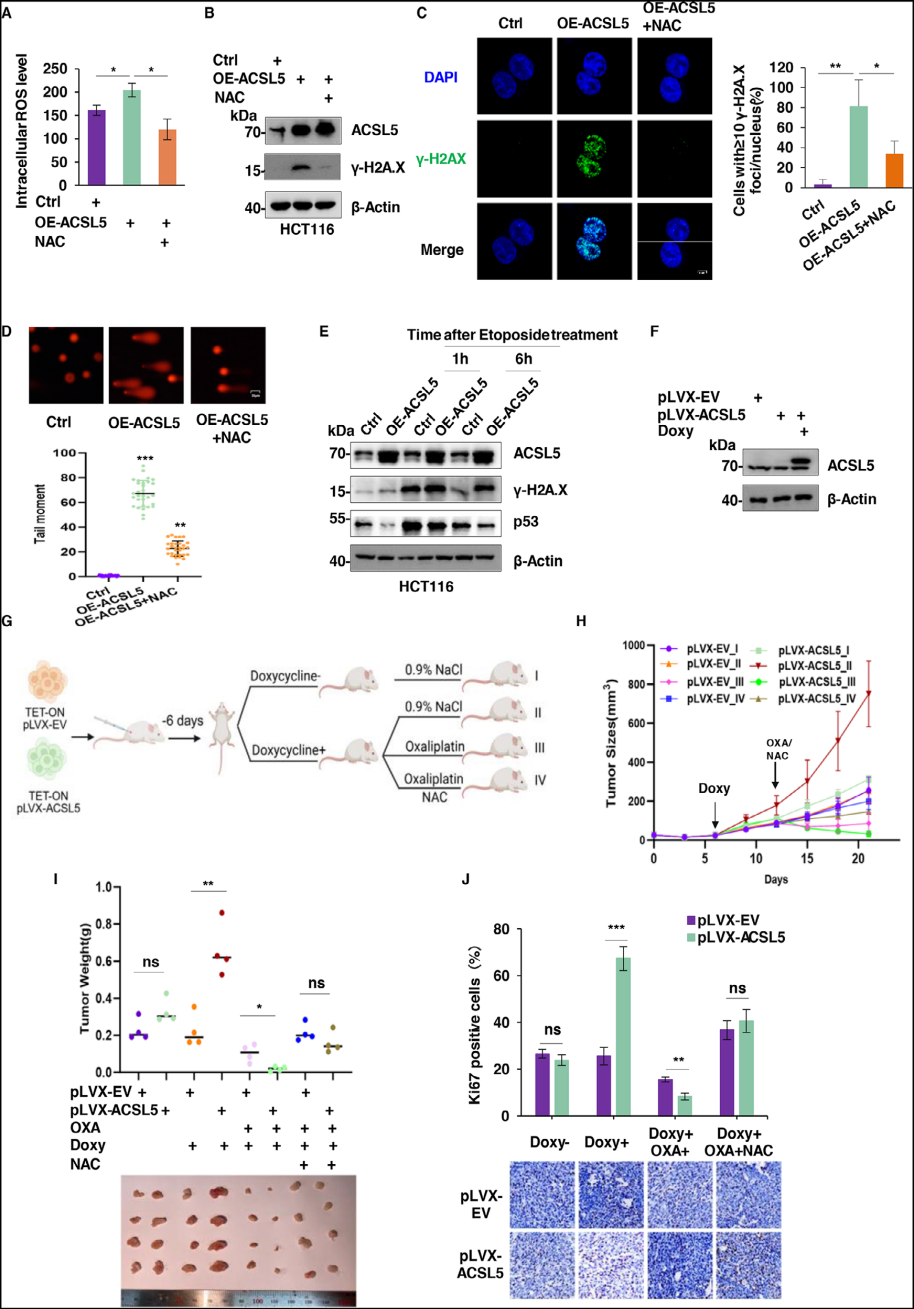
ACSL5 promotes ROS production, induces DNA damage, and increases sensitivity to Oxaliplatin. A) Intracellular ROS level measurements undertaken in HCT116 cells transduced with control (pCDH‐EV) or ACSL5 overexpression (pCDH‐ACSL5) in combination with 5 mm N‐Acetylcysteine (NAC) treatment for 4 h. B) Western blot analysis of the cells in (A) detecting ACSL5 and γ‐H2A.X. β‐Actin was used throughout as a loading control. C) Representative confocal images (left) showing immunostaining against γ‐H2A.X and nuclei decorated by DAPI in the cells from (A) (scale bar,5 µm). The percentage of cells with ≥10 γ‐H2A.X foci/nucleus was determined from 10 random cells (right). D) DNA damage measurements undertaken in the cells from (A) using comet assays. Representative single cell electrophoresis images (upper) and analysis of tail moments using CaspLab software (bottom, n = 30 cells/group, AU: arbitrary units). E) Control (pCDH‐EV) or ACSL5 overexpressing (pCDH‐ACSL5) HCT116 cells were untreated or treated with 40 um etoposide for 2 h before changing the medium prior to cell harvest at 1 or 6 h followed by Western blot analyses against ACSL5, γ‐H2A.X, and p53. F‐J) Western blot analysis verifying the doxycycline (Doxy)‐inducible expression of ACSL5 established in HCT116 cells using the pLVX system (F). Protocol for inducing ACSL5 overexpression with doxycycline (2 mg kg^−1^) and treating with oxaliplatin (OXA) and NAC (1 mg mL^−1^) in subcutaneous HCT116 xenografts established in nude mice (G). Growth curves of the xenografts (H), final tumor weights (I), together with determination of mitotic rates in the tumors using immunohistochemical (IHC) staining against Ki67 (J) are shown. Representative IHC images (bottom; scale bars, 20 µm) and proliferative indices determined from the relative numbers of Ki67^+^ cells (top). (A‐F) represent three independent experiments. (A, C, D, H‐J) Data are mean ± SD: (A, C, D, J) two‐way ANOVA; (H, I) two‐tailed unpaired *t* test. ns, not significant, ^*^ *P* < 0.05, ^**^ *P* < 0.01, ^***^ *P* < 0.001.

The process of β‐oxidation generates reactive oxygen species (ROS), primarily within the mitochondria. Appropriate levels of ROS are required to maintain cell homeostasis, with higher levels acting as signaling molecules to activate the antioxidant defense system and stress response pathways. However, excessive ROS levels can trigger oxidative stress and damage biological macromolecules such as lipids, proteins, and DNA. Thus, the enhancement of fatty acid β‐oxidation by ACSL5 would be predicted to increase intracellular ROS levels. Indeed, increased ROS levels were detected in ACSL5 overexpressing cells, which were otherwise inhibitable by treatment with the ROS scavenger NAC (N‐acetylcysteine) (**Figure** **7**A). Conversely, relatively diminished ROS levels were detected after ACSL5 knockdown, while the addition of the ROS inducer elesclomol effectively reversed this phenomenon (Figure S5A, Supporting Information).

Nonetheless, it remained undefined as to whether the changes in ROS resulting from ACSL5 overexpression were sufficient to cause cellular damage. Therefore, we considered the impact of elevated cellular ROS levels from the perspective of DNA, noting increases in the protein levels of the DNA damage response marker γ‐H2A.X together with increased numbers of γ‐H2A.X foci (Figure 7B,C). Moreover, increases in the number of DNA strand breaks were indicated by the longer comet tails detected in ACSL5 overexpressing cells during single‐cell gel electrophoresis assays (Figure 7D). Consistently, knockdown of ACSL5 reduced the tonic levels of γ‐H2A.X, whereas Elesclomol treatment promoted increased γ‐H2A.X expression and enhanced the formation of nuclear foci and DNA strand breaks (Figure S5B–D, Supporting Information). Collectively, these findings underscore the positive relationship between increased ACSL5 levels, intracellular ROS, and DNA damage.

Given our findings that ACSL5 induces DNA damage but also inhibits p53 expression, this raises a further intriguing point, given the function of p53 as a key regulator of DNA repair.^[^ ^34^ ^]^ On this basis, we speculated that ACSL5 may not only exacerbate DNA damage through promoting ROS but also by weakening p53‐dependent DNA repair mechanisms. We tested this idea by examining the sensitivity of ACSL5 manipulated HCT116 cells to Etoposide, an inhibitor of topoisomerase II that induces the accumulation of DNA double‐strand breaks. Using γ‐H2A.X as a proxy for DNA damage, we found that ACSL5 overexpressing cells displayed relatively higher γ‐H2A.X levels than controls, whereas ACSL5 knockdown cells displayed reduced γ‐H2A.X levels (Figure 7E; Figure S5E, Supporting Information). As anticipated, ACSL5 overexpression reduced p53 induction in response to Etoposide, with γ‐H2A.X levels inversely tracking with p53 in these experiments. This further implies that ACSL5 exacerbates DNA damage not only through increased ROS but also that its ultimate feedback restraint on p53 levels may also weaken p53‐dependent DNA repair mechanisms.

Indeed, reinforcing this point, bioinformatic interrogation of transcriptomic data after ACSL5 knockdown showed the profound enrichment of DNA‐associated pathways, including DNA replication and numerous DNA Damage Response (DDR) pathways, including the upregulation of key genes involved in DNA replication, base excision repair (BER), as well as the p53 pathway (Figure S6A–C, Supporting Information). To verify these transcriptomic changes reflected alterations in the DDR, we assessed the expression and activity of the base excision repair (BER) and nucleotide excision repair (NER) pathways. Our analysis of colon cancer cells and murine colon tissues from the in vitro and in vivo models showed that ACSL5 depletion upregulated the expression of the core BER enzymes OGG1 and APE1, while XRCC1 levels remained unchanged. However, no obvious changes were detected in the levels of NER‐related factors (XPA, XPC, and ERCC1) (Figure S6D,E, Supporting Information). Functional assays supported these changes where ACSL5 depletion enhanced BER activity but not NER activity (Figure S6F,G, Supporting Information). Collectively, these data support a dual‐hit model: ACSL5 fuels genomic instability not only by generating ROS‐induced DNA damage but also by simultaneously suppressing p53‐dependent DNA damage responses.

Additionally, since the induction of DNA damage in tumor cells underlies the actions of many chemotherapeutic agents,^[^ ^35^ ^]^ we reasoned that manipulating ACSL5 would influence the activity of drugs commonly used to treat colorectal cancer, such as the fluoropyrimidine 5‐FU, the platinum‐based drugs oxaliplatin and cisplatin, and other topoisomerase inhibitors such as irinotecan. Measuring cell viability using CCK‐8 assays showed that ACSL5 overexpression was particularly effective in sensitizing cells to the effects of oxaliplatin, with a significant reduction in IC50 values compared with control cells, although the same effects were not observed with 5‐FU, cisplatin, and irinotecan treatments (Figure S5F, Supporting Information). Moreover, the addition of NAC reversed the cell viability differences between control and ACSL5 overexpressing cells (Figure S5G, Supporting Information), indicating that the potentiation of oxaliplatin activity resulted from excessive ROS.

Lastly, given the impact of ACSL5 overexpression on oxaliplatin sensitivity, we investigated if this phenomenon was relevant in an in vivo context using mouse xenografts. Toward this, we established a doxycycline‐inducible TET‐ON system in the HCT116 cell line to permit inducible ACSL5 overexpression, thereby allowing tumors to establish tumors of comparable size prior to treatment without the effects of altered ACSL5 levels (Figure 7F). After the subcutaneous tumors became palpable, mice were administered doxycycline in combination with or without oxaliplatin, along with NAC. No tumor growth differences were recorded in mice not receiving doxycycline, but as predicted from our in vitro findings, significant promotion of tumor growth occurred after doxycycline treatment to induce ACSL5. Notably, ACSL5 overexpression tumors were significantly smaller than controls in mice receiving oxaliplatin, while the addition of NAC treatment, at least partially, reversed the growth inhibitory effects of oxaliplatin and nullified the growth differences between control and ACSL5 overexpression tumors (Figure 7G–I). Immunohistochemical staining against the proliferation marker Ki67 showed that the growth‐promoting abilities of ACSL5 overexpression, as well as the effects of oxaliplatin, were directly related to changes in cell proliferation (Figure 7J). Taken together, these findings indicate that overexpression of ACSL5 promotes tumor proliferation while increasing tumor sensitivity to the chemotherapeutic drug oxaliplatin.

### 2.8 ACSL5 Enhances the Sensitivity of Colorectal Tumors to Chemotherapy

**Figure 8:**
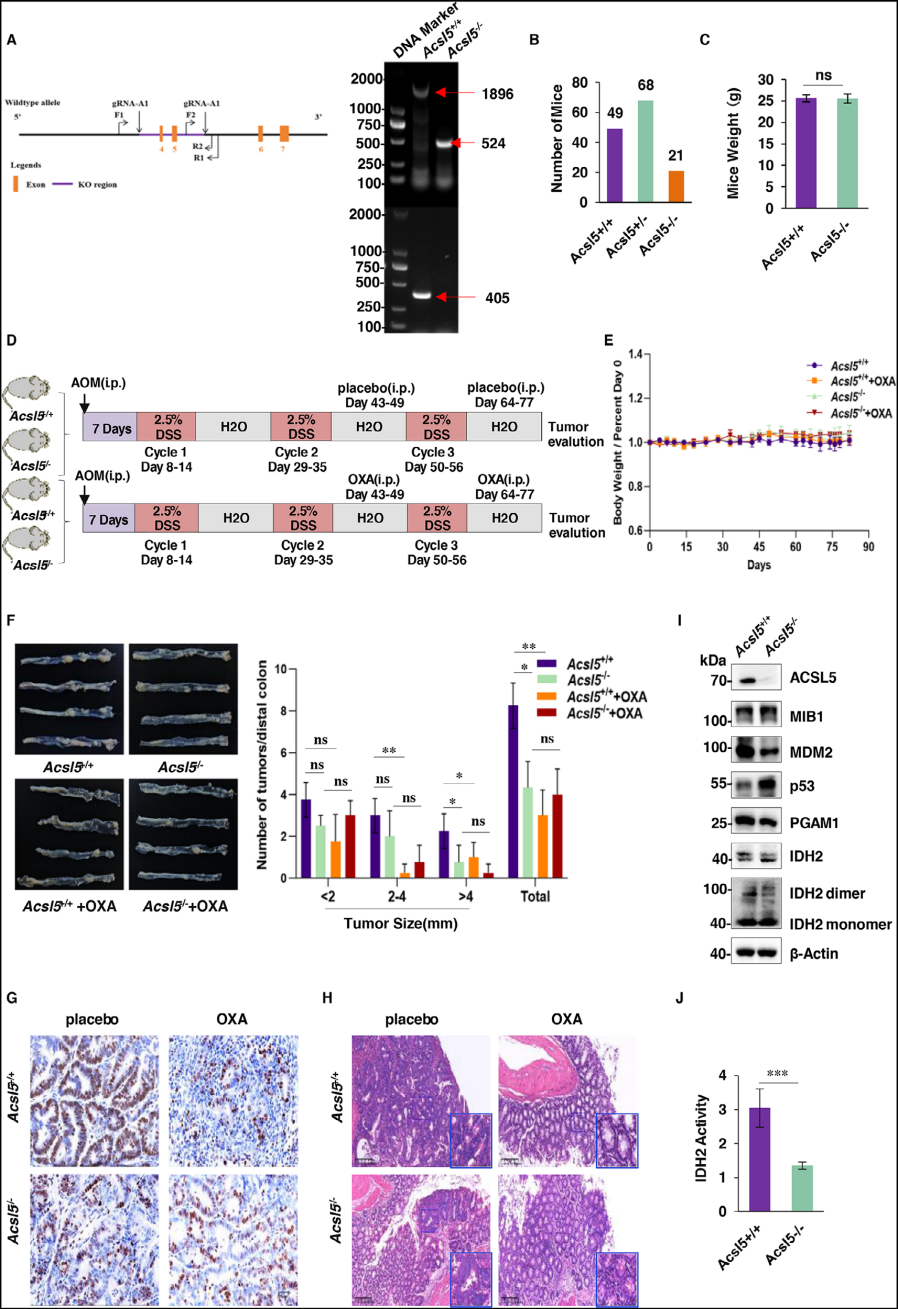
ACSL5 enhances chemotherapy sensitivity in orthotopic mouse models. A) CRISPR‐Cas9‐based strategy to delete exons 4 and exons 5 to knockout *Acsl5* in mice (left). Representative PCR analysis to validate the genotype of wildtype (*Acsl5* ^+/+^) and KO‐ACSL5 (*Acsl5* ^−/−^) mice (right). B) Frequency of genotypes obtained from backcross matings between *Acsl5* ^+/−^ mice. C) Body weights of 8‐week‐old male *Acsl5* ^+/+^ versus *Acsl5* ^−/−^ mice. D) Experimental design of the AOM/DSS‐induced colon tumor model using 8‐week‐old male mice incorporating the timeline for treatments with saline placebo or oxaliplatin (OXA). E) Body weight measurements of the mice from (D) were recorded every 3–4 days. F) Images (left, scale bars, 2 mm) and tumor burden calculation (right) of the distal colon in mice from mice in (D) at completion (day 84) of the AOM/DSS and treatment protocol. G, H) IHC staining against Ki67 (G, scale bars, 20 µm) and accompanying H&E histopathological staining (H, scale bars, 200 µm) of AOM/DSS‐induced colorectal lesions in *Acsl5* ^+/+^ and *Acsl5* ^−/−^ mice. I) Western blot analysis of colon tumor tissues from (D) against MIB1, MDM2, p53, PGAM1 and IDH2 with β‐Actin used as a loading control. J) IDH2 enzyme activity detected in colon tumor tissues from (D). (A, C, G‐J) represent three independent experiments. (C, E, F, J) Data are mean ± SD; (C, J) two‐tailed unpaired *t* test; (F) two‐way ANOVA with Tukey's test. ns, not significant, ^*^ *P* < 0.05, ^**^ *P* < 0.01, ^***^ *P* < 0.001.

Based on the effects of ACSL5 on tumor cell growth and treatment responses, our findings clearly propose a role for ACSL5 in tumor progression, although its role in tumor initiation was unclear. This prompted us to investigate the tumorigenic functions of ACSL5 in a mouse genetic model. We were able to generate global *Acsl5* knockout animals, although backcrossing homozygous (*Acsl5^−/−^*) mice with heterozygous (*Acsl5^+/−^*) mice failed to produce homozygous offspring. Moreover, crossings between *Acsl5^+/−^* heterozygous mice yielded less than expected Mendelian ratios of *Acsl5* ^−/−^ homozygotes (1/7), implicating a mild fecundity defect in *Acsl5^+/−^* heterozygous mice (**Figure** **8**A,B). Nonetheless, viable *Acsl5* ^−/−^ mice showed no significant differences in body weight compared to their wild‐type littermates (Figure 8C), permitting us to proceed with the experimental plan.

We established the AOM/DSS‐induced orthotopic colorectal cancer model comparing *Acsl5* ^−/−^ with *Acsl5* ^+/+^ mice to assess the effects of ACSL5 on tumor development, also including an oxaliplatin treatment arm to determine responses to chemotherapy (Figure 8D). Body weights were continually monitored during the protocol to assess morbidity, although we observed no significant differences among treatment groups throughout the experimental period (Figure 8E). With the placebo intervention, we observed that AOM/DSS induced significantly fewer and smaller distal colorectal tumors in *Acsl5* ^−/−^ mice compared to *Acsl5* ^+/+^ mice. In comparison, oxaliplatin treatment significantly diminished tumor burden in *Acsl5* ^+/+^ but not *Acsl5* ^−/−^ mice (Figure 8F). The rates of tumor growth were reflected by the proportion of Ki67‐positive cells within tumor tissues along with the *Acsl5^−/−^* tumor tissues containing a significantly lesser proportion of mitotic cancer cells (Figure 8G,H). Additionally, we examined the expression of MIB1, MDM2, p53, PGAM1, along with IDH2 expression and enzymatic activity in colorectal tumor tissues from the *Acsl5* ^−/−^ and *Acsl5* ^+/+^ mice. Genetic loss of ACSL5 was associated with increased p53 and decreased MDM2 expression, as well as the diminished dimer formation and activity of IDH2 (Figure 8I,J), providing consistency with the mechanisms disclosed by our in vitro experiments.

**Figure 9:**
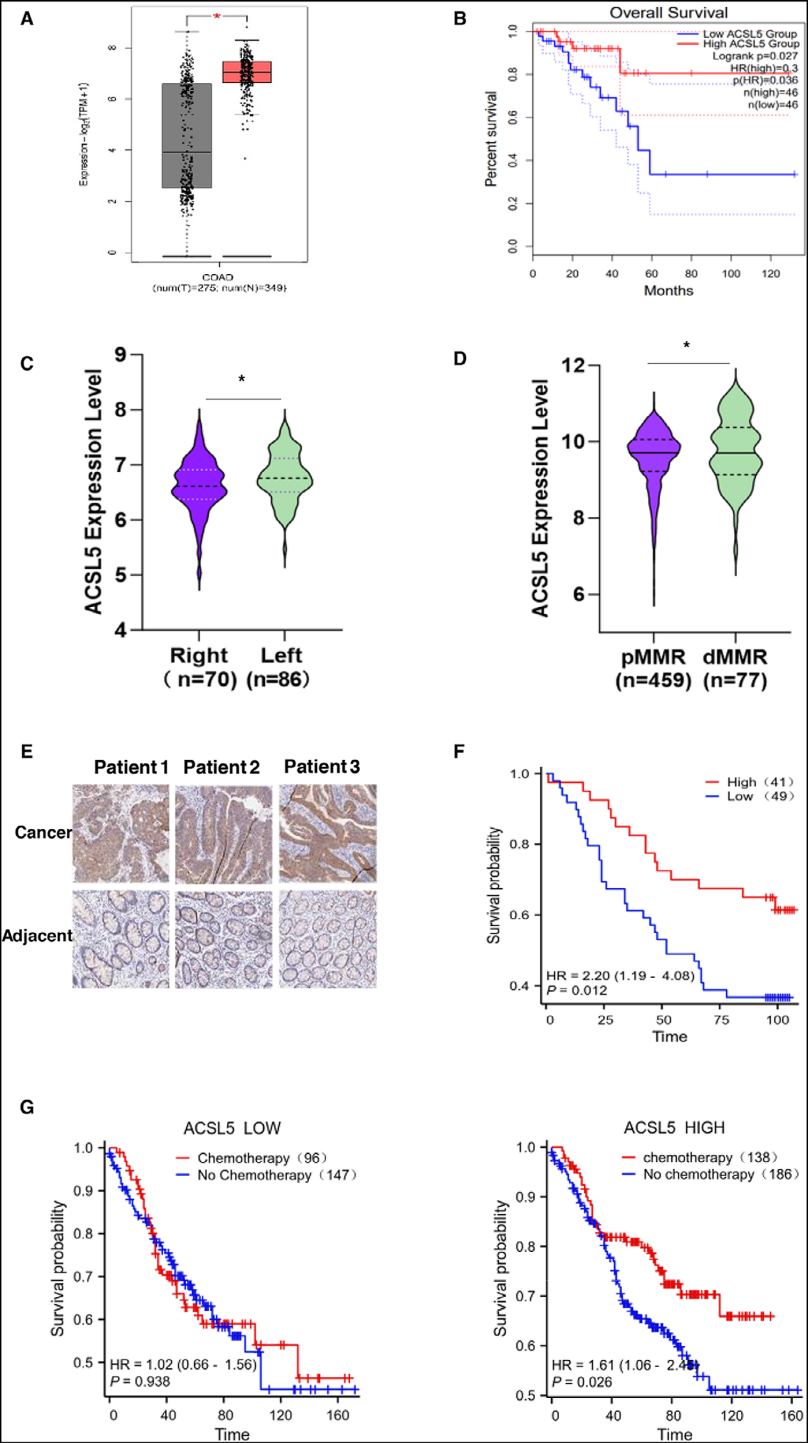
ACSL5 is a prognostic indicator of favorable chemotherapy‐related outcomes in colorectal cancer. A, B) Box and Whisker plot comparing ACSL5 expression between normal (n=275) and colorectal cancer tissues (n=349) in the TCGA‐COAD database (A), along with Kaplan–Meier curves of overall survival stratified by median ACSL5 expression in available patients from the TCGA‐COAD cohort (B). C) Violin plot of ACSL5 expression in right‐sided versus left‐sided colorectal cancers from the GSE103479 dataset (GEO) showing increased ACSL5 expression in the more favorable prognosis left‐sided cases.^[^ ^48^ ^]^ D) Violin plot of ACSL5 expression in proficient mismatch repair (pMMR) versus deficient MMR (dMMR) CRC cases from the GEO GSE40967 dataset showing increased ACSL5 expression in the more favorable prognosis dMMR cases.^[^ ^49^ ^]^ E, F) Representative IHC staining against ACSL5 in paired adjacent normal and colorectal cancer tissues (scale bars, 20 µm) (E) together with Kaplan‐Meier curves of overall survival in patients with colorectal cancer based on ACSL5 expression (F). ACSL5 expression was determined using IHC in a 90‐patient TMA‐based cohort with stratification into ACSL5 low and high cases using median staining intensities. G) Kaplan–Meier curves for overall survival in patients from the GEO GSE40967 dataset.^[^ ^49^ ^]^ Cases were stratified into low (left) and high (right) ACSL5 expression groups using mean ACSL5 transcript levels respectively, in concert with chemotherapy treatment status. Statistical notation: (A, C, and D) two‐tailed unpaired *t* test; (B, F, and G) log‐rank test. ^*^ *P* < 0.05.

Lastly, to clarify the clinical significance of ACSL5 expression in colorectal cancer, we analyzed expression and survival data in the TCGA COAD dataset. Overall comparisons of tissue expression revealed that ACSL5 mRNA levels were differentially upregulated in colorectal cancer versus normal colonic tissues (**Figure** **9**A). Further stratification of cases by median expression showed that patients whose tumors expressed higher ACSL5 expression had significantly better prognoses (Figure 9B). Other established features in CRC that predict clinical behavior and prognosis involve primary tumor location (right‐ or left‐sided) as well as microsatellite instability (MSI) status, where ≈15% of cases display MSI due to deficient DNA mismatch repair (dMMR). We found that higher ACSL5 expression was enriched in better survival‐associated left‐sided tumors (Figure 9C; Table S3, Supporting Information) while also being elevated in the improved prognosis subgroup of cases showing deficient DNA mismatch repair (dMMR) in combination with high MSI (Figure 9D; Table S4, Supporting Information). However, since these findings were based on transcriptome data, we analyzed an alternative cohort of CRC patients using immunohistochemical staining against ACSL5 on tissue microarrays to evaluate ACSL5 protein levels (Table S5, Supporting Information). Consistently, we found that ACSL5 staining in tumor nests was higher than in adjacent normal colonic epithelia (Figure 9E), with survival analyses indicating that high tumor ACSL5 expression was associated with better patient outcomes (Figure 9F). This presents an intriguing paradox: while our data confirm that ACSL5 promotes tumor cell proliferation, its association with better prognosis suggests its clinical impact is driven by mechanisms beyond proliferation alone.

Notably, given that elevated ACSL5 levels were shown to potentiate the effects of oxaliplatin, we hypothesized that its role might be contextually dependent upon chemotherapy. To test this, we stratified colorectal cancer patients by both ACSL5 expression and chemotherapy status. Strikingly, chemotherapy had no significant effect on survival in tumors with low ACSL5 expression. In contrast, patients with high ACSL5‐expressing tumors who received chemotherapy showed significantly improved survival (Figure 9G; Table S4, Supporting Information). Thus, the relative expression of ACSL5 plays a significant role in colorectal cancer outcomes, advancing opportunities for ACSL5 as a biomarker and treatment target.

## 3 Discussion

**Figure 10:**
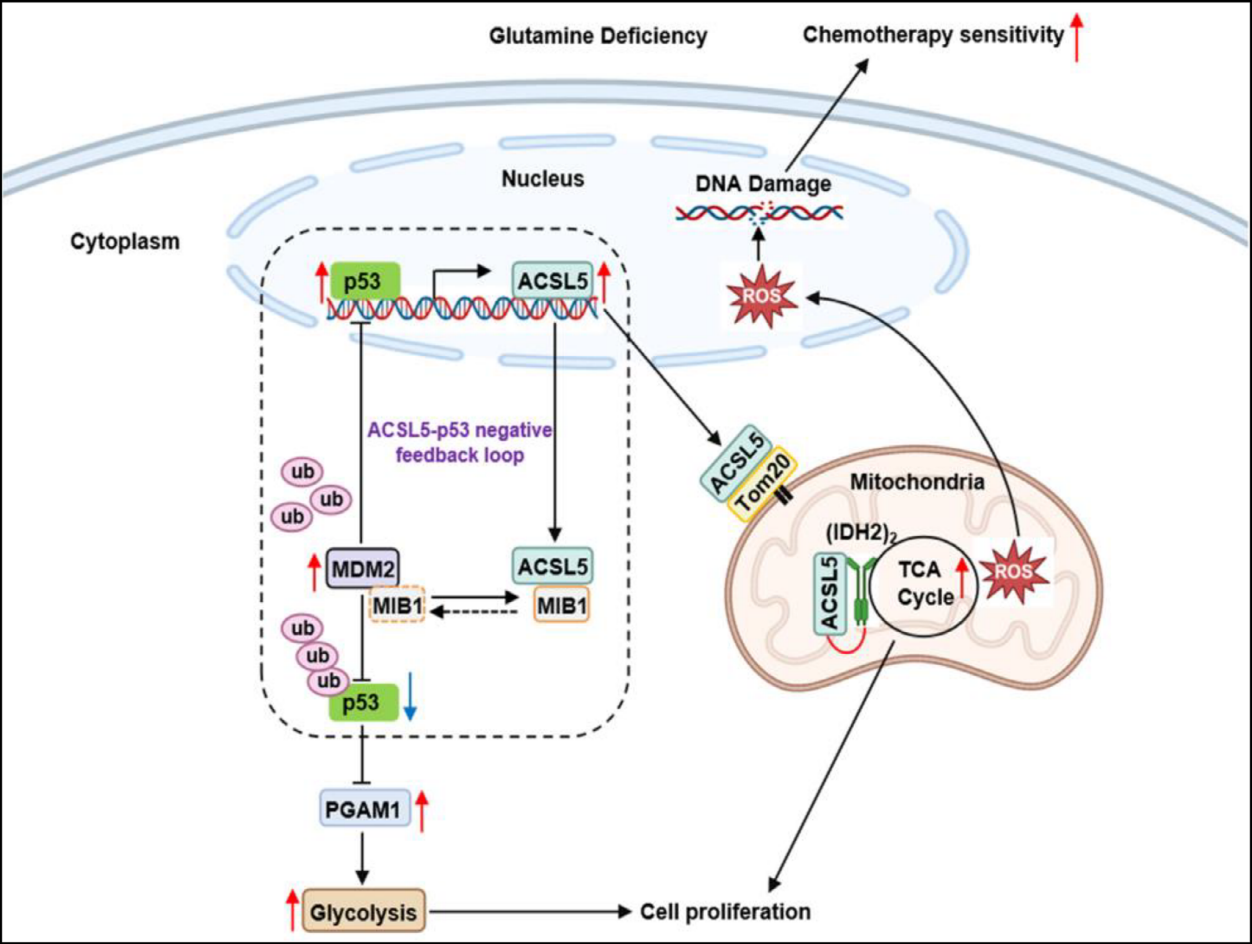
Working model of the dual metabolic actions of ACSL5 in colorectal cancer and its impact on chemotherapy. Deficiencies in glutamine availability trigger upregulation of p53, which in turn transcriptionally upregulates the expression of ACSL5. This increases ACSL5 protein pools in both the cytoplasm and mitochondria with distinct actions. Cytoplasmic ACSL5 competes with MDM2 for MIB1 binding, thus inhibiting the ubiquitination and degradation of MDM2 by MIB1, a mechanism that dampens the upregulation of p53 by reducing its protein stability. This feedback significantly relieves the inhibition of PGAM1 by p53, stimulating glycolysis. Alternatively, ACSL5 enters mitochondria by binding to TOM20, directly promoting the dimerization and activation of IDH2 toward enhancing the tricarboxylic acid (TCA) cycle. These collective functions aid in the provision of energy and metabolites to ensure cell survival and proliferation within the harsh conditions of the tumor microenvironment. The heightened metabolic activities associated with increased ACSL5 expression also drive net increases in ROS levels, leading to DNA damage, together with the weakening of DNA repair mechanisms initiated via p53. These effects create a cellular environment primed to be susceptible to DNA‐damaging chemotherapeutic agents.

We initiated our study to uncover new insights into survival mechanisms enacted by tumor cells under glutamine‐limited conditions, a condition relevant to the tumor microenvironment where heightened nutrient demands often outweigh their availability. We observed that ACSL5 was commonly upregulated by glutamine withdrawal in a panel of mixed‐origin carcinoma cell lines. Focusing on colorectal cancer cells, we subsequently found that ACSL5 oversees adaptive metabolic changes that support the survival and proliferation under glutamine restriction. The supplementation of both cytoplasmic and mitochondrial pools of ACSL5 protein in turn elicits compartmentalized effects on both glycolysis and mitochondrial respiration, respectively. The increases in ACSL5 were shown to substantially involve its transcriptional upregulation by p53, which itself is upregulated following glutamine deprivation. Notably, the increased cytoplasmic ACSL5 levels establish a feedback loop to modulate p53 expression. The latter was shown to involve competitive binding interactions between ACSL5 to MIB1, subsequently diverting MIB1 from targeting MDM2 for degradation, thereby reducing p53 protein stability. This establishes the conditions to fine‐tune glycolysis via relieving the transcriptional inhibition of PGAM1 by p53, with increased PGAM1 levels promoting glycolysis. We also found that ACSL5 interacts with TOM20 to enter mitochondria, where it positively influences IDH2 dimerization toward sustaining mitochondrial respiration and TCA cycle flux. Together, these mechanisms coordinately sustain energy production and metabolic intermediate supplies toward promoting cancer cell proliferation and survival under glutamine‐limiting conditions, as illustrated in our working model (**Figure** **10**).

A logical follow‐on question concerns the significance of ACSL5 in the oncology clinic. Because of their implicit role in fatty acid metabolism, ACSL family proteins have been considered as therapeutic targets, although individual differences exist in their tissue expression, cellular localization, not to mention substrate preferences.^[^ ^36^ ^]^ For instance, ACSL1, ACSL4, and ACSL5 are considered to localize to mitochondria, at least in part.^[^ ^37^ ^]^ Our results support this conclusion while revealing additional details, namely the essential role of its predicted mitochondrial transmembrane sequence^[^ ^24^ ^]^ and the recognition of ACSL5 by TOM20 to facilitate its mitochondrial entry. It must also be considered that ACSL proteins are positioned as dual actors in cancer, some showing oncogenic functions while others, such as ACSL5 are mainly reported to exert tumor suppressive functions. For example, early pathological reports suggested a progressive decline in ACSL5 expression between epithelia, premalignant adenomas, and adenocarcinomas sequence in small intestine carcinogenesis^[^ ^38^ ^]^ whereas low ACSL5 expression in primary colorectal tumor samples was suggested to predict early cancer recurrence, although not long‐term patient survival.^[^ ^39^ ^]^ A recent report employing autologous mouse models showed that ACSL5 functions as a tumor suppressor in melanoma and lung cancer, but in a nuanced manner, showing that ACSL5 promotes tumor antigen presentation.^[^ ^40^ ^]^ Nonetheless, the role of ACSL proteins in cancer must be considered in context, for instance, ACSL5 was reported to act as an oncogene in glioma, allowing tumor cells to overcome the deleterious effects of acidosis in the tumor microenvironment through upregulation of pro‐tumorigenic factor midkine.^[^ ^41^ ^]^ Our investigations involving TCGA data and tissue microarrays showed that ACSL5 was commonly increased in primary colorectal cancers, and, much like reported for melanoma and lung cancer,^[^ ^40^ ^]^ increased ACSL5 expression was associated with improved patient outcomes. Nevertheless, our functional studies revealed that a general positive relationship exists between ACSL5 levels and tumor cell growth both in vitro and in vivo. This apparent contradiction provides difficulties in assigning a purely oncogenic or tumor suppressive role to ACSL5. However, further evidence provided by our study serves to reconcile this point. We found that only colorectal patients whose tumors expressed high levels of ACSL5 benefited from chemotherapy, thus assigning a key impact of ACSL5 on treatment efficacy.

Our experiments further uncovered the likely basis of the effects of ACSL5 on chemotherapy. We found that ROS generation was a significant consequence of heightened ACSL5 expression, resulting from its enhancement of fatty acid β‐oxidation and accelerated glucose metabolism. Excessive ROS triggers oxidative stress, which we found to significantly increase DNA damage. Furthermore, by inhibiting p53 expression as part of a negative feedback loop, ACSL5 induction also effectively impairs DNA damage repair mechanisms. The synergistic effects of these mechanisms augment tumor cell sensitivity to oxaliplatin chemotherapy, proposing how increased ACSL5 expression contributes to improved patient prognosis. Nevertheless, other impacts of ACSL5, which must be considered, involve the effects of ACSL5 expression on tumor immunity. A recent study in lung cancer showed that ACSL5 expression potentiates the expression of major histocompatibility complex class I (MHC‐I) antigens, which effectively potentiates the actions of immune checkpoint blockade therapies.^[^ ^40^ ^]^ However, the general positive relationship between ACSL5 and tumorigenesis observed with manipulations of human colorectal cancer cells lines is not to be discounted, especially given independent data from our orthotopic carcinogenesis model. Namely, that less tumors arose in the null background under the acute inflammatory pressure of AOM/DSS treatment. This conceivably is due to relative differences in genomic stability caused by unresolved DNA damage; indeed, the orthotopic cancer tissues phenocopied our cell line data, showing ACSL5 wildtype tumors with higher PGAM1 expression and IDH2 activity, along with lessened p53 expression, underscoring the effects of ACSL5 on metabolism and DNA repair mechanisms.

Lastly, it is worth noting that Bowman and colleagues previously reported that ACSL5 knockout mice display metabolic alterations of reduced fat but increased lean mass resulting from increased energy utilization.^[^ ^42^ ^]^ Nonetheless, there was no overall change in body weight in their study, with the phenotype only becoming evident in 4‐month‐old mice. We did not encounter this phenomenon, but our experiments were completed before reaching the 4‐month stage. However, we did find that genome‐wide loss of ACSL5 may induce infertility, with haploinsufficiency impacting embryonic survival to some degree, although the exact mechanisms remain unclear.

In summary, our study sought to elucidate novel signaling pathways enabling cancer cells to sustain proliferation and survival under glutamine deprivation. We identified ACSL5 as a key mediator that concurrently enhances glycolytic flux and TCA cycle activity, thereby overcoming glutamine deficiency. Further mechanistic investigations revealed that ACSL5 not only drives metabolic reprogramming but also promotes tumor initiation and progression in colorectal cancer. Notably, ACSL5‐induced metabolic hyperactivity led to accumulation of DNA damage, compounded by its interference with DNA repair pathways, serving to directly influence the sensitivity of cancer cells to genotoxic stress. This dual role—metabolic sustainment and genomic instability—suggests ACSL5 as a critical facilitator of tumor evolution by increasing mutational burden and malignant potential. Our work provides a foundational framework for targeting ACSL5 in colorectal and other glutamine‐dependent cancers, offering new avenues for precision therapy.

## 4 Experimental Section

### Cell Lines

Cell lines were obtained from multiple sources: HCT116, HEK293T, HepG2, and HT29 were purchased from the American Type Culture Collection (ATCC); RKO and 786‐O were acquired from the Shanghai Institute of Cell Biology, Chinese Academy of Sciences; KYSE450 and A549 were respectively obtained from Henan Provincial People's Hospital and the University of Science and Technology of China (Table S6, Supporting Information). All cell lines were routinely cultured in either Dulbecco's Modified Eagle Medium (DMEM; GIBCO) or RPMI 1640 medium (GIBCO), supplemented with 10% fetal bovine serum (FBS; Biological Industries) and 1% penicillin‐streptomycin (Solarbio) in a humidified incubator at 37 °C with 5% CO2. Cell identity was authenticated using STR analysis, and regular contamination checks were performed using mycoplasma detection and cell culture contamination detection kits (Thermo Fisher Scientific).

### Animals

Six‐ to eight‐week‐old BALB/c nude mice were purchased from GemPharmatech, and transgenic mice (C57BL/6JCya‐Acsl5 em1C/Cya) were obtained from Cyagen Biosciences (Suzhou). The mice were housed in the SPF‐grade animal facility of the Zhengzhou University Animal Experiment Center. All mice were maintained under pathogen‐free conditions and used strictly in accordance with protocols approved by the Zhengzhou University Animal Care and Use Committee. The study complied with all relevant ethical regulations for animal research. All mice in the xenograft and transgenic experiments were subjected to treatments in a randomized manner. Mice exhibiting weight loss exceeding 15% after adding DSS to the drinking water were excluded for humane reasons. Except for husbandry purposes and embryo collection, all animals used in this study were male. Animal experiments related to this project were approved by the Zhengzhou University Laboratory Animal Ethics Committee (Approval Number: ZZU‐LAC20230915).

### Cell Viability Assays

Cells were seeded at a density of 4000 cells per well in 96‐well plates and cultured overnight. Subsequently, 10 µL of CCK‐8 reagent was added to each well according to the manufacturer's instructions, followed by incubation at 37 °C for 1.5 h. Absorbance at 450 nm was measured using a Varioskan LUX microplate reader (Thermo Fisher Scientific) with treatment data normalized to control wells.

### RNA Interference and Gene Overexpression

Lentiviral particles were generated in 293T cells transfected with the indicated plasmids using Lipofectamine 2000 (Invitrogen). For knockdown experiments, PLKO.1‐based shRNAs were co‐transfected with pREV, pGag, and pVSVG at a ratio of 2:2:2:1. For overexpression experiments, pCDH/pLV2‐TetOn, pspax2, and pmd2.g were co‐transfected at a ratio of 2:2:1. After 48 h, the supernatants were collected, filtered through a 0.45 µm filter, and incubated with target cells in the presence of 8 µg mL^−1^ polybrene (Sigma). Following 48 h of incubation, transduced cells were selected with 2 µg mL^−1^ puromycin. Target sequences are listed in Table S7 (Supporting Information).

### Proteomics and LC‐MS/MS Analysis

For proteomic‐seq, cells cultured with normal or glutamine‐free DMEM for 48 h were lysed and subjected to trypsin digestion. The resulting peptides were successively processed by TMT labeling, High pH Reverse Phase Fractionation (HPRP), and nano LC‐MS/MS analysis. The LC‐MS/MS analysis was performed using a Q Exactive mass spectrometer (Thermo Scientific) coupled with an EASY‐nLC 1200 ultra‐high‐pressure liquid chromatography system (Thermo Scientific). Peptides were separated at a flow rate of 300 nL min^−1^. The MS raw data were combined and analyzed using MaxQuant (v1.5.3.17) for identification and quantification. Tandem mass spectra were searched against the human uniprot database (9606 entries) with a reverse decoy database for false discovery rate (FDR) control. An initial search was set at a precursor mass window of 10 ppm. The search followed an enzymatic cleavage rule of Trypsin/P and allowed a maximal of two missed cleavage sites and a mass tolerance of 20 ppm for fragment ions. Carbamidomethyl on Cys was specified as fixed modification, and Oxidation (M) was specified as a variable modifications. FDR was adjusted to ≤ 0.01.

### Clone Formation

Cells were seeded into six‐well plates at a density of 1000 cells per well and cultured for 2 weeks. The cells were then fixed with 4% formaldehyde and stained with 0.5% crystal violet. After staining, the cells were washed three times with PBS and imaged using the Bio‐Rad GelDoc XR^+^ imaging system (Bio‐Rad). Cell colonies were quantitatively analyzed using the ImageJ plugin “ColonyArea”.

### Glycolysis and Mitochondrial Stress Tests

Glycolysis and mitochondrial stress tests were performed using the Seahorse XFe96 Analyzer (Agilent) according to the manufacturer's instructions using 24‐well XF Cell Culture Microplates. Cells were seeded at a density of 4000 cells per well were allowed to attach before conducting standard glycolysis stress tests using the sequential addition of glucose (10 mm), oligomycin (1 mm), and 2‐DG (50 mm) to the XF base medium. Alternatively, mitochondrial stress tests were performed by adding oligomycin (1.5 µm), FCCP (1.0 µm), and Rot/AA (0.5 µm).

### Subcellular Fractionation

A suspension of 2 × 10⁷ total cells was divided into three parts at a ratio of 1:2:7 for the extraction of total protein, nuclear, cytoplasmic, and mitochondrial fractions. Total proteins were extracted using conventional cell lysis, while nuclear, cytoplasmic, and mitochondrial extracts were prepared following the instructions of the Nuclear and Cytoplasmic Extraction Kit (Thermo 78833). For the nuclear fractions, cells were mixed with 500 µL of CER I and incubated on ice for 10 min before the addition of 27.5 µL of CER II, followed by vortex mixing and centrifugation at 16 000 g for 5 min. Pellets were resuspended in 250 µL of NER and incubated on ice for 40 min, followed by centrifugation at 16 000 g for 10 min, with supernatants collected as the nuclear extract. For the cytoplasmic and mitochondrial fractions, cells were incubated with Reagent A on ice for 2 min before homogenization using 50 strokes of a dounce homogenizer. Thereafter, 800 µL of Reagent C was added before centrifuging the mixture at 700 g for 10 min, collecting the supernatant, which was further centrifuged at 12 000 g for 15 min. The final supernatant was collected as the cytoplasmic fraction, and the pellet, washed with Reagent C, was used as the mitochondrial fraction. All fractions were quantified using the BCA method, and equal protein amounts were analyzed by Western blot.

### Immunofluorescence (IF)

Cells were cultured on coverslips, and after attachment, switched to a glutamine‐free medium for 48 h. Mito‐Tracker dye (Thermo Fisher) was then added, and the cells were incubated for 30 min. After staining, the cells were washed three times with PBS and fixed with 4% formaldehyde at room temperature. Following fixation, the cells were washed three times with PBS and permeabilized with permeabilization buffer (0.1% Triton X‐100 in PBA) for 5 min. Subsequently, the cells were blocked with 4% BSA at room temperature for 1 h and incubated overnight at 4 °C with ACSL5 primary antibodies. The next day, the cells were washed three times with PBS and incubated at room temperature with Alexa Fluor 488‐conjugated secondary antibody for 1 h. Cell nuclei were then stained with DAPI for 5 min, followed by PBS washing. The coverslips were mounted using Fluorescence Mounting Medium (Dako) and images obtained with a laser confocal microscope (Leica SP8).

### Immunohistochemistry (IHC)

The human colorectal cancer tissue microarray was procured from Shanghai Outdo Biotech Company, Ltd. (Ethics Approval No. SHYJS‐CP‐1404002). Tumor tissues from subcutaneous xenografts in nude mice or colorectal tissues from C57BL/6J mice were excised and fixed with 4% formaldehyde, followed by paraffin embedding and sectioning. The sections were deparaffinized and rehydrated before undergoing hematoxylin and eosin (H&E) staining or immunohistochemical (IHC) staining with antibodies recognizing Ki67 (1:2000 dilution) or ACSL5 (1:1000 dilution). Bound antibodies were detected and visualized using the rabbit polymer detection system (ZSGB‐BIO) and the DAB substrate kit (ZSGB‐BIO) before collecting digital images using the TISSUEFAXS system (TissueGnostics Asia). Ki67‐positive (Ki67⁺) cells were then quantified using ImageJ software.

### Comet Assays

Comet assays were performed as described previously using the Comet Assay kit (BD Biosciences).

### Quantitative PCR (qPCR)

Total RNA was extracted from the collected cells using the GeneJET RNA Purification Kit (Thermo Fisher Scientific, #K0731) according to the manufacturer's instructions. A total of 1 µg of RNA was reverse transcribed into cDNA using the PrimeScript RT Reagent Kit (TaKaRa). Subsequently, a 20 µL qPCR reaction mixture was prepared using 2×TB Green Premix Ex Taq II (TaKaRa), ROX reference dye, cDNA, and 0.4 µm primers. The samples were amplified for 35 cycles using the StepOnePlus real‐time PCR system (Thermo Fisher Scientific). The relative expression levels of the target gene, normalized to the GAPDH reference gene, were calculated using the 2^^−ΔΔCT^ method. Primer sequences are listed in Table S8 (Supporting Information).

### Luciferase Reporter Assays

Experiments were conducted using the Dual Luciferase Reporter detection system according to the manufacturer's instructions (Promega, E1910). The pGL3 construct containing the ACSL5 promoter was co‐transfected with the Renilla luciferase plasmid in combination with the other indicated manipulations. After transfection for 24 h, firefly luciferase and Renilla luciferase measurements were conducted using the Varioskan LUX microplate reader (ThermoFisher) with Renilla luciferase values used to normalize firefly luciferase activity.

### Immunoprecipitation

Cells were lysed in IP lysis buffer (50 mm Tris‐HCl, pH 7.4, 150 mm NaCl, 1 mm EDTA, 5% glycerol, 0.4% Triton X‐100, 0.4% NP‐40) supplemented with a protease inhibitor cocktail at 4 °C for 30 min. Subsequently, lysates were centrifuged at 12 000 rpm at 4 °C for 20 min, and supernatants were collected. Ten percent of the supernatant was used as the input control, while the remaining lysate was incubated with the primary antibodies overnight at 4 °C, followed by capture with Protein A/G beads for 2 h at 4 °C. The beads were washed five times with lysis buffer and eluted with 2×SDS loading buffer. The samples were heated at 95 °C for 10 min prior to electrophoresis and Western blot analysis.

### Western Blotting

Cell suspensions were lysed using RIPA buffer containing protease inhibitors, while tumor tissues were first homogenized in RIPA buffer using a tissue homogenizer. The samples were then centrifuged at 14 000 rpm at 4 °C for 25 min, and the clarified supernatants were collected for analysis. Protein concentrations were estimated using BCA assays and equal protein amounts separated by SDS‐PAGE before transfer onto nitrocellulose membranes. Membranes were blocked with 5% skim milk at room temperature, incubated with the specified primary antibodies overnight at 4 °C, and then incubated with species‐matched secondary antibodies at room temperature for 1 h. Protein bands were visualized using ECL chemiluminescence reagents and recorded with a chemiluminescence imaging system (Tanon). Semi‐quantitative analysis of the protein bands was performed using NIH ImageJ software.

### Chromatin Immunoprecipitation

ChIP assays (Beyotime) were performed according to the manufacturer's instructions using anti‐rabbit IgG as a negative control. The DNA fragments of interest were analyzed by semi‐quantitative using specific primers (Table S8, Supporting Information).

### Base Excision Repair (BER) Efficiency Assay

BER capacity was evaluated using a fluorescence‐based plasmid reactivation assay as previously described.^[^ ^43^, ^44^ ^]^ Briefly, cells were co‐transfected with a pEGFP‐C1 reporter plasmid containing an 8‐oxoG lesion within the coding sequence and an mCherry‐expressing plasmid as an internal transfection control. After 24 h, GFP and mCherry fluorescence were quantified by flow cytometry. BER efficiency was calculated as the ratio of GFP‐positive to mCherry‐positive cells, reflecting the extent of functional reporter reactivation following repair of the 8‐oxoG lesion.

### Nucleotide Excision Repair (NER) Efficiency Assay

NER capacity was evaluated using a dual‐luciferase host cell reactivation (HCR) assay as previously described.^[^ ^45^ ^]^ Pyrimidine dimers were induced in the pGL3 reporter plasmid by UV‐C light irradiation (254 nm, 300 J per m^−^^2^ for 300 s). Cells were co‐transfected with UV‐damaged or undamaged pGL3‐basic Firefly luciferase plasmids and a Renilla luciferase plasmid as an internal control. After 24 h, Firefly and Renilla luciferase activities were measured using the dual luciferase assay system (Promega). Nucleotide excision repair (NER) efficiency was calculated using the formula: Nucleotide Excision Repair (NER) Efficiency (%) = (Luminescence Value of Damaged Plasmids / Luminescence Value of Undamaged Plasmids) × 100.

### Mouse Genotyping

Toe samples from mice aged 7–10 days were collected and lysed according to the instructions of the mouse genotyping rapid detection kit (Biosharp), followed by PCR amplification. The PCR products were separated on a 1% agarose gel via electrophoresis and visualized and recorded using a gel imaging system (Tanon). Specific primers used to detect the ACSL5 wildtype and knockout alleles are listed in Table S7 (Supporting Information).

### Subcutaneous Tumor Model

For the knockdown xenograft model, HCT116 cells (5 × 10⁶) expressing Tet‐inducible negative control (sh‐Ctrl) or sh‐ACSL5 based on the Fh1tutg plasmid system^[^ ^46^ ^]^ were subcutaneously injected into the lateral back of 5‐week‐old male nude mice. After allowing ≈7 days for tumor establishment, ACSL5 knockdown was induced by adding doxycycline (2 mg mL^−1^) to the drinking water. Another 7 days later, the mice were switched to either glutamine‐free feed (TP 01A0L01, Trophic Animal Feed High‐Tech Co., Ltd, China) or control feed as indicated. Tumor size was measured every 3 days throughout the protocol, and when tumor volumes reached ≈1500 mm^3^, the mice were humanely euthanized and tumor tissues collected. Alternatively, for the overexpression/ chemotherapy model, HCT116 cells (5 × 10⁶) expressing Tet‐inducible negative control (Ctrl) or ACSL5 overexpression based on the pLVX system^[^ ^47^ ^]^ were subcutaneously injected into the lateral back of 5‐week‐old male nude mice. After allowing ≈7 days for tumor establishment, ACSL5 overexpression was induced by adding doxycycline (2 mg mL^−1^) to the drinking water. Tumor size was measured every 3 days, and when there was a significant difference in tumor size between the control group and the experimental group, oxaliplatin (1.5 mg kg^−1^) chemotherapy was administered intraperitoneally. When tumor volumes reached ≈1000 mm^3^, the mice were humanely euthanized and tumor tissues collected.

### AOM/DSS Induced Inflammatory Carcinogenesis Model

Eight‐week‐old male *Acsl5* ^−/−^ mice and their littermate wild‐type *Acsl5* ^+/+^ mice were intraperitoneally injected with azoxymethane (AOM, Sigma–Aldrich, 10 mg kg^−1^). Seven days later, 2% dextran sulfate sodium (DSS, MP Biomedicals) was added to the drinking water for one week, followed by two weeks of regular drinking water. The DSS cycle was repeated a further two times. In the 7th and 9th weeks, PBS or oxaliplatin (1.5 mg kg^−1^) chemotherapy was administered intraperitoneally. Mouse body weight measurements were conducted regularly throughout the experiment to monitor morbidity, with all animals humanely euthanized on day 77 of the protocol.

### Quantification and Statistical Analysis

Statistical analysis was performed using GraphPad Prism software to evaluate differences between groups. For comparisons between two groups, a two‐tailed Student's *t*‐test was used. For comparisons among three groups, one‐way analysis of variance (ANOVA) followed by Tukey's post‐hoc test was applied. For binary comparisons, two‐way ANOVA with Tukey's post‐hoc test was used. A *P*‐value of less than 0.05 was considered statistically significant, with results indicated as follows: ns (not significant), ^*^ *p* < 0.05, ^**^ *p* < 0.01, ^***^ *p* < 0.001.

## Conflict of Interest

The authors declare no conflict of interest.

## Author Contributions

M.W. and W.H. jointly supervised this work. M.W., S.T., X.Y., X.S., and W.H. conceived and designed the study. S.T. performed all parts of the experiment and data analysis. Q.J. and Z.S. participated in the construction of plasmids. Q.Z., Z.S., and Y.L. coordinated key animal and clinical experimentation. Q.Z., Z.S., X.Y., and Q.Z. provided the necessary technical support. S.T., R.F.T., and M.W. wrote the manuscript.

## Supporting information



Supporting Information

## Data Availability

The data that support the findings of this study are available from the corresponding author upon reasonable request.
