# Supplementary material for: ACSL5 Regulates Glucose Metabolism and Chemotherapy Sensitivity in Colorectal Cancer Cells under Glutamine Deficiency
Source: Adv Sci (Weinh). 2025 Dec 8;13(7):e10801. doi: 10.1002/advs.202510801 (PMC12866851; doi:10.1002/advs.202510801)
Supplement: Supplementary file 1 — Supporting Information [file ADVS-13-e10801-s001.docx]

**Supporting Information**

**ACSL5 Regulates Glucose Metabolism and Chemotherapy**

**Sensitivity in Colorectal Cancer Cells under**

**Glutamine Deficiency**

Shuai Tian^1^, Qiaoxia Zhang^1^, Xuedan Sun^2^, Rick Francis Thorne^1^, Zeyuan Shi^1^, Qiang Ji^1^, Zhangran Sun^3^, Yuanxiang Lu^4^, Qun Zhao^5^, Xianjun Yu^5^✉, Wanglai Hu^6^✉ and Mian Wu^1^✉

1. Translational Research Institute of Peoples Hospital of Zhengzhou University and Academy of Medical Science, Tianjian Laboratory of Advanced Biomedical Sciences, Zhengzhou University, Henan International Joint Laboratory of Non-coding RNA and Metabolism in Cancer, Zhengzhou, 450003 China.

2. Department of Hepatobiliary Surgery, Centre for Leading Medicine and Advanced Technologies of IHM, The First Affiliated Hospital of USTC, Division of Life Sciences and Medicine, University of Science and Technology of China, Hefei, 230031 China.

3. Department of Pharmacology, School of Basic Medical Sciences, Academy of Medical Science, Zhengzhou University, Zhengzhou, 450000 China.

4. Department of Breast Surgery, Zhengzhou University People Hospital & Henan Provincial People’s Hospital, Zhengzhou, 450003 China.

5. School of Basic Medical Sciences, Hubei Key Laboratory of Embryonic Stem Cell Research, Biomedical Research Institute, Hubei University of Medicine, Shiyan, 442000 China.

6. Academy of Medical Science, Tianjian Laboratory of Advanced Biomedical Sciences, State Key Laboratory of Metabolic Dysregulation & Prevention and Treatment of Esophageal Cancer, Zhengzhou University, Translational Research Institute, People's Hospital of Zhengzhou University, Henan International Joint Laboratory of Non-coding RNA and Metabolism in Cancer, Zhengzhou, 450003 China.

**Figure S1. Supplementary figures related to Figure 1.**

A) Proteomic sequencing was performed on HepG2 cells cultured with or without glutamine for 48 hours and data analyzed to determine differentially expressed proteins (Table S1). The Box and Whisker plot depicts the normalized FC expression values of the indicated upregulated and downregulated candidate genes (log2 (Gln-/Gln+) value greater than 2 and a P-value (Gln- vs. Gln+) less than 0.05).

B) Heatmap comparing the expression of candidate genes from (A) based on qPCR analyses of representative carcinoma cells including the colorectal (HCT116), lung (A549), liver (HepG2), breast (KYSE450) and renal (786-O) lines cultured with or without glutamine for 48 hours.

C) Western blot analysis of ACSL5 protein levels in whole cell lysates (Wcl) or subcellular fractions including cytosol (Cy), nucleus (Nu), and mitochondria (Mt) in HCT116 cells after 24 hours treatment with DMSO carrier or CB-839 (3 μM), respectively. The fidelity of the nuclear, mitochondrial and cytosolic fractions was verified by detection of PARP, VDAC1 and β-Tubulin, respectively.

D) Representative confocal images of HCT116 cells treated for 24 hours with DMSO carrier or CB-839 (3μM) before conducting immunofluorescence staining against ACSL5 (green) in concert with MitoTracker (red) and DAPI staining to decorate mitochondria and nuclei, respectively. Scale bars, 5 μm.

E, F) Mitochondria were purified from HCT116 and RKO cells were subjected to digestion without or with proteinase K at the indicated concentrations before conducting Western blotting against ACSL5 along with markers of outer mitochondrial membranes (OMM; TOM70, TOM20) and inner mitochondrial membranes (IMM; COXIV, ATP5A1) (E). Further Proteinase K protection assays performed on purified mitochondria as per (F) in the absence and presence of the permeabilizing agent Triton X-100 (F).

G, H) HCT116 cells transduced with an shRNA control (shCtrl) or shTOM40 (G) or shTOM70 (H) constructs were cultured with or without glutamine for 48 hours before Western blot analysis of ACSL5 in whole cell lysates or the indicated subcellular fractions (fraction fidelity demonstrated as per (C)).

1. Data represent mean and interquartile ranges with statistical differences determined by t test. (B-H) represent three independent experiments. *P < 0.05, **P < 0.01.

**Figure S2. Supplementary figures related to Figure 3.**

A) Schematic illustrating consensus binding sites for the indicated transcription factors in the ACSL5 proximal promoter region.

B-E) HCT116 cells were transduced with control shRNA (pLKO.1) or shRNAs targeting SP1 (B), HSF1 (C), STAT3 (D) and NF-kB (E), respectively, before culturing cells in the presence or absence of glutamine for 48 hours. The relative levels of ACSL5 mRNA were determined by qPCR (top) with Western blotting used to verify the effects of knockdown (bottom). β-Actin was used throughout as a loading control.

F-I) The relative levels of ACSL5 mRNA were determined by qPCR (top) in HCT116 cells (F), RKO cells (G), p53 null (*TP53^-/-^*) HCT116 cells (H) or p53-mutant HT29 cells (I) subjected to glutamine withdrawal for 24 or 48 hours. Western blotting was used in concert to measure the levels of ACSL5 and p53 protein (bottom).

(B-I) represent three independent experiments. Data are mean ± SD: two-tailed multiple t test; ns, not signiﬁcant, *P < 0.05, **P < 0.01, ***P < 0.001.

**Figure S3. Supplementary figures related to Figure 4.**

A) Relative p53 mRNA levels measured by qPCR in HCT116 cells transduced with shCtrl or two independent shRNAs targeting ACSL5.

B) Ubiquitination assays against p53 were conducted in sh-ctrl or sh-MDM2 HCT116 cells in the indicated combinations with OE-ACSL5 (pCDNA-3.1-ACSL5) followed by MG132 treatment. Inputs and p53 immunoprecipitants were subject to immunoblotting against ubiquitin (ub), p53, MDM2 and ACSL5 as indicated.

C) Relative MDM2 mRNA levels measured by qPCR in HCT116 cells transduced with shCtrl or two independent shRNAs targeting ACSL5.

D) Venn diagram illustrating the intersection of E3 ubiquitin ligases showing binding ACSL5 and MDM2. This analysis is based on the mass spectrographic analysis conducted in this study (Table S2) in concert with published data recording the protein-interactome of MDM2.^[30]^

E) Western blotting analysis of the levels of ACSL5, MDM2 and known MDM2-targeting ligases in HCT116 cells following transduction with control (pCDH-EV) or ACSL5 overexpression (pCDH-ACSL5) constructs.

F, G) Western blotting against MIB1 and MDM2 in HCT116 cells transfected with control siRNAs or two independent siRNAs targeting MIB1 (F). Cycloheximide chase assays conducted on the cells in (F) comparing the levels and stability of MDM2 using Western blot (G).

H) Western blotting analysis of the concentration-dependent effects of ACSL5 on MDM2 levels. HCT116 cells were transfected with control (pCDNA3.1-EV) or with different amounts of (pCDNA3.1-ACSL5) constructs to achieve gradient overexpression of ACSL5.

I-K) Schematic illustrating the functional domain organization of MIB1 and corresponding truncation constructs (I). 293T cells were transfected with Flag-MDM2 (J) or Flag-ACSL5 (K) along with the indicated combinations of control HA-vector (EV), or HA-tagged versions of wildtype (WT) or truncated MIB1 mutants. After immunoprecipitation with anti-HA antibodies, coprecipitating MDM2 or ACSL5 proteins were revealed with blotting using anti-Flag antibodies.

(A-C, E-H, J, K) represent three independent experiments. (A, C) Data are mean ± SD: two-tailed multiple t test; ns, not signiﬁcant.


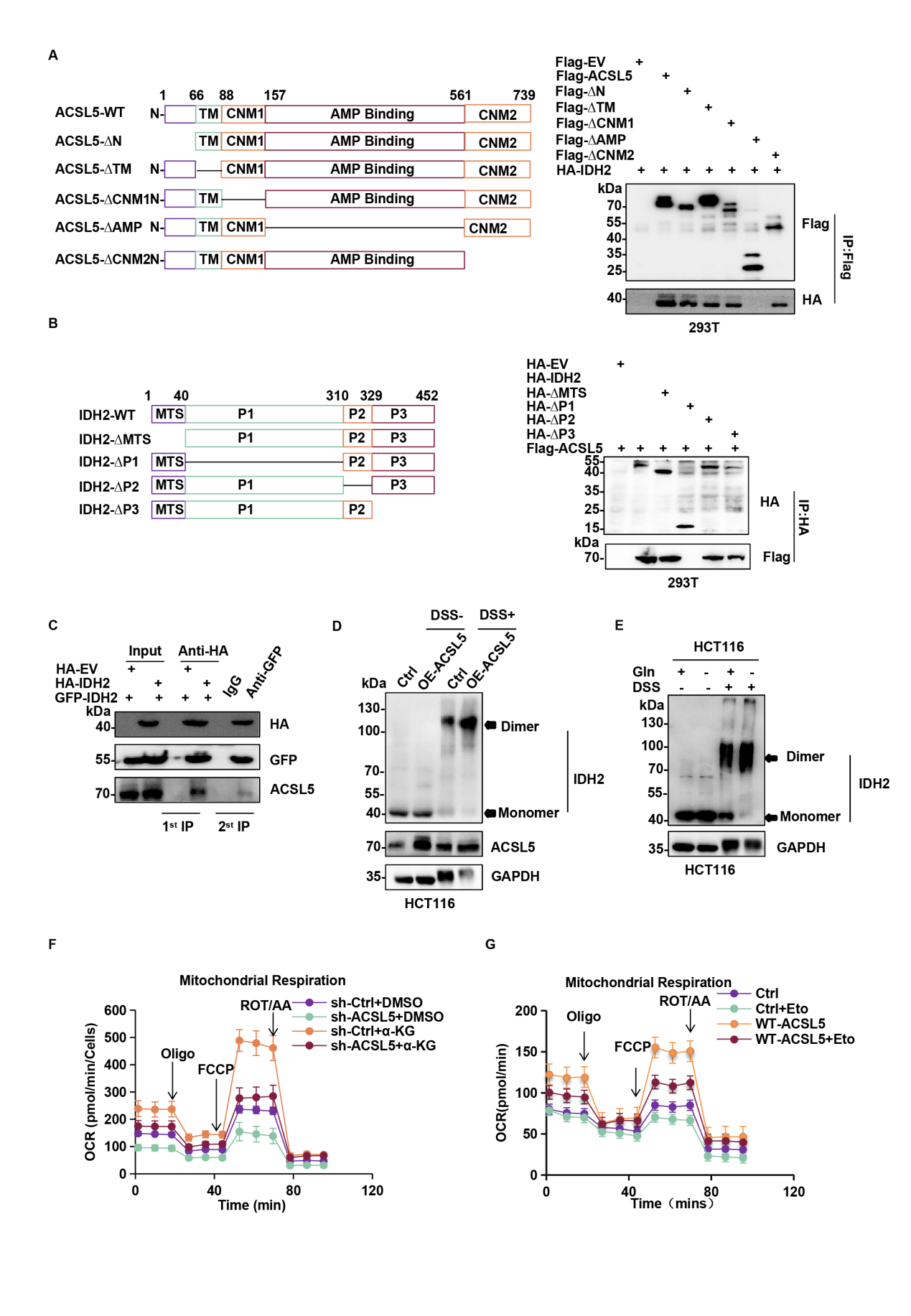


**Figure S4. Supplementary figures related to Figure 6.**

A) Schematic illustrating the protein domain organization of ACSL5 and the corresponding design of Flag-tagged truncation constructs (left). 293T cells were transfected with HA-IDH2 in combination with empty Flag-vector (EV) or Flag-tagged versions of wildtype (WT) or truncated ACSL5 mutants as indicated. After immunoprecipitation with anti-Flag antibodies, coprecipitating IDH2 was revealed with blotting using anti-HA antibodies (right).

B) Schematic illustrating the protein domain organization of IDH2 and the corresponding design of HA-tagged truncation constructs (left). 293T cells were transfected with Flag-ACSL5 in combination with empty HA-vector (EV) or HA-tagged versions of wildtype (WT) or truncated IDH2 mutants as indicated. After immunoprecipitation with anti-HA antibodies, coprecipitating ACSL5 was revealed with blotting using anti-Flag antibodies (right).

C) Two phase immunoprecipitations performed using HCT116 cells co-transfected with HA-IDH2 and GFP-IDH2. Lysates were first immunoprecipitated with anti-HA antibodies before elution and sequential immunoprecipitation with anti-GFP antibodies followed by immunoblotting of samples with HA, GFP and ACSL5 antibodies.

D) HCT116 cells transduced with control (pCDH-EV) or with OE-ACSL5 (pCDH-ACSL5) were treated without or with DSS crosslinker before subjecting whole cell lysates to Western blotting against IDH2, ACSL5 and GAPDH.

E) HCT116 cells cultured under glutamine-replete and -deprived conditions were treated without or with DSS crosslinker before subjecting whole cell lysates to Western blotting against IDH2 and GAPDH.

F) Mitochondrial stress tests measuring oxygen consumption rates (OCR) were performed on HCT116 cells transduced with control (sh-Ctrl) or sh-ACSL5 lentiviruses in combination with 5 mM α-KG-treatment for 24 hours using the Seahorse XF Analyzer.

G) Mitochondrial stress tests measuring oxygen consumption rates (OCR) were performed on HCT116 cells transfected with pCMV-EV (Ctrl) or pCMV-ACSL5 (WT-ACSL5) using the Seahorse XF Analyzer after pretreatment with DMSO carrier or 50 μM Etomoxir for 24 hours.

(A-G) represent three independent experiments. (F-G) Data are mean ± SD.

**Figure S5. Supplementary figures related to Figure 7.**

A) Intracellular ROS level measurements undertaken in HCT116 transduced with shctrl or ACSL5 knockdown (shACSL5) in combination with 100 nM Elesclomol treatment for 6 hours.

B) Western blot analysis of the cells in (A) detecting ACSL5 and γ-H2A.X. b-Actin was used throughout as a loading control.

C) Representative confocal images (left) showing immunostaining against γ-H2A.X and nuclei decorated by DAPI in the cells from (A) (scale bar, 5 μm). The percentage of cells with ≥10 g-H2A.X foci/nucleus were determined from 10 random cells (right).

D) DNA damage measurements undertaken in the cells from (A) using comet assays. Representative single cell electrophoresis images (upper) and analysis of tail moments using CaspLab software (bottom, n = 30 cells/group, AU: arbitrary units).

E) Control (sh-Ctrl) or ACSL5 knockdown (sh-ACSL5) HCT116 cells were untreated or treated with 40 uM etoposide for 2 hours before changing the medium prior to cell harvest at 1 or 6 hours followed by Western blot analyses against against ACSL5, γ-H2A.X and p53.

F) Dose titration curves (left) and calculated IC50 values (right) for HCT116 cells bearing control (pCDH-EV) or ACSL5 overexpression (pCDH-ACSL5) constructs for 5-FU, Oxaliplatin, Cisplatin and irinotecan, respectively.

G) Relative cell viability measurements conducted using CCK-8 assays undertaken in HCT116 cells transduced with control (pCDH) or ACSL5 overexpression (pCDH-ACSL5) constructs combination with NAC (5 mM, 4 hours) and oxaliplatin (OXA: 5 uM, 24 hours).

(A-G) represent three independent experiments. (A, C-D, F-G) Data are mean ± SD: two-way ANOVA. ns, not signiﬁcant, **P < 0.01, ***P < 0.001.

**Figure S6. Supplementary figures related to Figure 7.**

A-C) Transcriptome sequencing was performed on HCT116 cells transduced with shCtrl or shACSL5. Bubble plot shows top 20 KEGG upregulated pathway enrichment derived from differentially expressed genes (A). GSEA enrichment score lines plots based on ranked gene expression data illustrating the DNA replication / mismatch repair / double-strand break repair via homologous recombination and base excision repair pathways (B). Bubble plot of expression changes in core genes related to DNA replication (left) and p53 signaling pathways (right) in HCT116 cells following knockdown of ASCL5 (C).

D) HCT116 cells were transduced with shCtrl or two independent shRNAs targeting ACSL5 before undertaking Western blotting against key BER and NER proteins. Representative blot panel (left) and normalized protein levels measured by densitometry from n = 3 independent experiments (right).

E) Western blot measuring the levels of key BER and NER proteins in colon tissues of wildtype (*Acsl5*^+/+^) and KO-ACSL5 (*Acsl5*^-/-^) mice (top). Normalized protein levels measured by densitometry (bottom; n = 3 tissues/group).

F) Base excision repair (BER) efficiency measurements comparing HCT116 cells transduced with control (shCtrl) or shACSL5 lentiviruses were conducted using plasmid reactivation assays. Cells were co-transfected with pEGFP-c1 plasmids carrying BER damage substrates and the internal control mCherry plasmid to normalize transfection efficiency. The ratio of GFP-positive cells to mCherry-positive cells was analyzed by flow cytometry after 24 h to calculate BER efficiency. Representative flow cytometric gating (left) and quantitation (right) from n = 3 replicates.

G) Nucleotide excision repair (NER) efficiency measurements comparing HCT116 cells transduced with control (shCtrl) or shACSL5 lentiviruses were conducted using the Host Cell Reactivation (HCR) reporter system. Cells were co-transfected with either undamaged or UV-damaged PGL3-basic plasmids containing pyrimidine dimers in combination with the internal reference Renilla luciferase plasmid. Dual luciferase assays were performed after 24 h to measure firefly and Renilla luminescence with NER repair efficiency calculated as a percentage (%) of damaged to undamaged plasmids.

(D, F, G) represent three independent experiments; (D-G) Data are mean ± SD: two-way ANOVA with Tukey’s test; ns, not signiﬁcant, *P < 0.05, **P < 0.01.

**Table S1.** Metabolism-related candidate proteins identified by proteomic screening of HepG2 cells after culture with or without glutamine for 48 hours (related to Figure S1).

| candidate proteins | | Ctrl | Gln- |  |  |
| --- | --- | --- | --- | --- | --- |
| Protein | Gene Name | average mock | average Gln | Gln/mock | t test p value |
| P08833 | IGFBP1 | 80713666.67 | 294793333.3 | 3.652334797 | 0.03356928 |
| Q96KC2 | ARL5B | 47765666.67 | 172708333.3 | 3.61574213 | 0.030607802 |
| Q9C0B1 | FTO | 133813333.3 | 393840000 | 2.943204464 | 0.004396617 |
| O60779 | SLC19A2 | 138443333.3 | 328920000 | 2.375845713 | 0.002431647 |
| Q9ULC5 | ACSL5 | 2734033333 | 5863533333 | 2.144645883 | 0.000828781 |
| Q8TB72 | PUM2 | 108746333.3 | 230763333.3 | 2.122033233 | 0.001208729 |
| P30086 | PEBP1 | 15395666667 | 7690600000 | 0.499530171 | 0.00156566 |
| Q99956 | DUSP9 | 3542200000 | 1473900000 | 0.416097341 | 0.002434422 |
| P62942 | FKBP1A | 845846666.7 | 348346666.7 | 0.411831932 | 0.011801737 |
| P16278 | GLB1 | 795706666.7 | 256346666.7 | 0.322162271 | 0.001481807 |
| O95864 | FADS2 | 981616666.7 | 215866666.7 | 0.219909333 | 0.004386329 |
| Q92947 | GCDH | 417546666.7 | 90358333.33 | 0.216402957 | 0.005187092 |

**Table S2.** Mass spectrometry identification of E3 ubiquitin ligases binding to ACSL5 and Mdm2 (related to Figure S3).

| **Table S2.1** E3 ubiquitin ligases recovered in anti-ACSL5 immunoprecipitates. | | | | | | | |
| --- | --- | --- | --- | --- | --- | --- | --- |
| Accession | Protein | Coverage [%] | # Unique Peptides | # Peptides | # AAs | MW [kDa] | calc. pI |
| P19474 | TRIM21 | 15 | 6 | 6 | 475 | 54.1 | 6.38 |
| P49792 | RANBP2 | 2 | 5 | 5 | 3224 | 358 | 6.2 |
| Q9C037 | TRIM4 | 9 | 4 | 4 | 500 | 57.4 | 8.1 |
| H3BQQ2 | ZNF598 | 4 | 3 | 3 | 849 | 93.2 | 8.53 |
| E9PH99 | ZNF451 | 3 | 3 | 3 | 1041 | 119.3 | 7.27 |
| Q9ULV8 | CBLC | 5 | 3 | 3 | 474 | 52.4 | 7.69 |
| Q5VTR2 | RNF20 | 1 | 1 | 1 | 975 | 113.6 | 5.94 |
| A0A087WTZ8 | NSMCE2 | 5 | 1 | 1 | 157 | 17.6 | 7.24 |
| Q86YT6 | MIB1 | 3 | 1 | 1 | 1006 | 110.1 | 6.92 |
| **Table S2.2** E3 ubiquitin ligases recovered in anti-Mdm2 immunoprecipitates. | | | | | | | |
| Accession | Protein | Coverage  [%] | # Unique Peptides | # Peptides | # AAs | MW [kDa] | calc. pI |
| F5H8E5 | USP7 | 6.48 | 4 | 4 | 1003 | 116.9 | 5.90 |
| F5H6W4 | March7 | 12.76 | 4 | 4 | 635 | 70.08008287 | 8.147949219 |
| Q96JH7 | VCPIP1 | 5.16 | 4 | 4 | 1222 | 134.2358576 | 7.195800781 |
| P49792 | RANBP2 | 1.52 | 4 | 4 | 3224 | 357.9740692 | 6.201660156 |
| Q13049 | TRIM32 | 5.51 | 3 | 3 | 653 | 71.94240221 | 6.976074219 |
| P61956-2 | SUMO2 | 30.99 | 2 | 2 | 71 | 8.106002765 | 5.414550781 |
| Q86YT6 | MIB1 | 1.49 | 1 | 1 | 1006 | 110.0657539 | 6.917480469 |
| B4DJ81 | NDUFS1 | 2.62 | 1 | 1 | 611 | 66.87900977 | 5.376464844 |
| Q5T447 | HECTD3 | 1.63 | 1 | 1 | 861 | 97.05145544 | 5.643066406 |
| V9GXZ5 | USP43 | 1.21 | 1 | 1 | 910 | 100.4120465 | 9.422363281 |
| O00308-3 | WWP2 | 2.78 | 1 | 1 | 431 | 51.0463488 | 6.277832031 |

**Table S3.** Patient characteristics and ACSL5 expression in primary colorectal tumors from the GSE103479 dataset (related to Figure 9C).

| GEO_accession | Location | Type of surgery | ACSL5 mRNA  expression (log2 normalized intensity signal) |
| --- | --- | --- | --- |
| GSM2772122 | Left | Sigmoid colectomy | 6.345235775 |
| GSM2772124 | Left | Sigmoid colectomy | 6.699858022 |
| GSM2772126 | Left | Sigmoid colectomy | 6.026093161 |
| GSM2772129 | Left | Sigmoid colectomy | 6.886156124 |
| GSM2772131 | Left | Anterior resection | 6.60202741 |
| GSM2772133 | Left | Sigmoid colectomy | 6.1644677 |
| GSM2772134 | Left | Sigmoid colectomy | 6.744746123 |
| GSM2772135 | Left | Sigmoid colectomy | 7.378408981 |
| GSM2772138 | Left | Sigmoid colectomy | 6.629765363 |
| GSM2772139 | Left | Left hemicolectomy | 6.705284729 |
| GSM2772140 | Left | Sigmoid colectomy | 6.598588347 |
| GSM2772141 | Left | Sigmoid colectomy | 6.620501752 |
| GSM2772144 | Left | Sigmoid colectomy | 6.626687565 |
| GSM2772146 | Left | Sigmoidectomy | 6.701156726 |
| GSM2772148 | Left | Sigmoidectomy | 6.8627725 |
| GSM2772151 | Left | Sigmoidectomy | 6.285975027 |
| GSM2772153 | Left | Sigmoidectomy | 6.90478032 |
| GSM2772154 | Left | Sigmoidectomy | 7.157972022 |
| GSM2772155 | Left | Sigmoidectomy | 6.792122626 |
| GSM2772156 | Left | Sigmoidectomy | 7.268817017 |
| GSM2772159 | Left | Sigmoidectomy | 6.48859728 |
| GSM2772160 | Left | Left colectomy | 6.936304302 |
| GSM2772162 | Left | Sigmoidectomy | 6.761251593 |
| GSM2772164 | Left | Sigmoidectomy | 6.896334236 |
| GSM2772165 | Left | Sigmoidectomy | 6.33623725 |
| GSM2772167 | Left | Sigmoidectomy | 7.055747784 |
| GSM2772168 | Left | Sigmoidectomy | 7.114873178 |
| GSM2772169 | Left | Left colectomy | 7.350553559 |
| GSM2772171 | Left | colectomy | 6.559607202 |
| GSM2772174 | Left | Sigmoidectomy | 7.639164126 |
| GSM2772175 | Left | Sigmoidectomy | 6.280338237 |
| GSM2772177 | Left | Sigmoidectomy- bladder-prostate | 7.679558571 |
| GSM2772180 | Left | Subtotal hemicolectomy (transverse left | 6.82216206 |
| GSM2772182 | Left | left hemicolectomy/cecum | 7.125079387 |
| GSM2772183 | Left | Left hemicolectomy | 7.011393684 |
| GSM2772185 | Left | UK | 7.314424141 |
| GSM2772186 | Left | Open Anterior Resection | 6.68271689 |
| GSM2772188 | Left | Laparoscopic Anterior Resection | 6.725045911 |
| GSM2772189 | Left | Open Anterior Resection | 6.851316795 |
| GSM2772192 | Left | Laparoscopic Anterior Resection | 6.027410657 |
| GSM2772194 | Left | Laparoscopic assisted High Anterior Resection | 6.67831503 |
| GSM2772196 | Left | Laparoscopic sigmoid colectomy | 6.76786112 |
| GSM2772197 | Left | Open Anterior Resection | 6.315097383 |
| GSM2772200 | Left | Laparoscopic Anterior Resection | 6.619529274 |
| GSM2772201 | Left | Laparoscopic Anterior Resection | 7.321984302 |
| GSM2772205 | Left | Sigmoid colectomy | 7.577537523 |
| GSM2772208 | Left | RAR | 6.110439154 |
| GSM2772209 | Left | Anterior Resection | 6.44494934 |
| GSM2772213 | Left | RAR | 6.403797565 |
| GSM2772215 | Left | left hemicolectomy RAR | 7.29450142 |
| GSM2772216 | Left | left hemicolectomy | 7.538997394 |
| GSM2772219 | Left | Sigmoidectomy | 6.875201203 |
| GSM2772222 | Left | Sigmoidectomy | 6.240595836 |
| GSM2772223 | Left | Sigmoidectomy | 6.773986964 |
| GSM2772224 | Left | Sigmoidectomy | 6.884149353 |
| GSM2772226 | Left | Sigmoidectomy | 6.161232066 |
| GSM2772228 | Left | Sigmoidectomy | 6.881834195 |
| GSM2772231 | Left | Left colectomy | 6.478882127 |
| GSM2772232 | Left | PROCECTOMY | 6.284264165 |
| GSM2772233 | Left | PROCECTOMY | 7.110219893 |
| GSM2772234 | Left | PROCECTOMY | 6.084694175 |
| GSM2772238 | Left | Sigmoidectomy | 7.313593936 |
| GSM2772241 | Left | Sigmoidectomy | 7.393771984 |
| GSM2772242 | Left | Sigmoidectomy | 6.309448021 |
| GSM2772244 | Left | Laparoscopic Anterior Resection | 6.635961643 |
| GSM2772246 | Left | Left hemicolectomy | 7.143739544 |
| GSM2772247 | Left | Laparoscopic left hemicolectomy | 7.010533369 |
| GSM2772248 | Left | Laparoscopic Anterior Resection | 5.473152384 |
| GSM2772249 | Left | Laparoscopic converted sigmoid colectomy | 6.616737781 |
| GSM2772250 | Left | Open Anterior Resection | 7.334215869 |
| GSM2772251 | Left | Open Anterior Resection | 7.341723663 |
| GSM2772252 | Left | Open Hartmann's Procedure | 6.540494102 |
| GSM2772253 | Left | Open subtotal colectomy | 6.860767421 |
| GSM2772254 | Left | Laparoscopic Anterior Resection | 6.544996028 |
| GSM2772256 | Left | Open High Anterior Resection | 7.02810263 |
| GSM2772258 | Left | Laparoscopic Anterior Resection | 7.318781585 |
| GSM2772259 | Left | Laparoscopic converted sigmoid colectomy | 6.00412279 |
| GSM2772260 | Left | Laparoscopic converted Anterior Resection | 6.579912597 |
| GSM2772262 | Left | Laparoscopic Anterior Resection | 6.891825789 |
| GSM2772269 | Left | Open Anterior Resection | 7.338904423 |
| GSM2772270 | Left | Sigmoid colectomy | 6.043480384 |
| GSM2772271 | Left | Sigmoid colectomy | 7.143229101 |
| GSM2772273 | Left | Sigmoid colectomy | 7.125647904 |
| GSM2772275 | Left | PROCECTOMY | 6.686295423 |
| GSM2772277 | Left | Left hemicolectomy | 6.517881452 |
| GSM2772178 | Right | Extended right hemicolectomy | 6.757331979 |
| GSM2772202 | Right | Right hemicolectomy | 6.779854439 |
| GSM2772204 | Right | Laparoscopic right hemicolectomy | 5.642982131 |
| GSM2772207 | Right | Right hemicolectomy | 6.434438969 |
| GSM2772123 | Right | Right hemicolectomy | 6.528765751 |
| GSM2772125 | Right | Right hemicolectomy | 7.221893161 |
| GSM2772127 | Right | Right hemicolectomy | 6.765788301 |
| GSM2772128 | Right | Right hemicolectomy | 5.396293803 |
| GSM2772130 | Right | Right hemicolectomy | 6.015958778 |
| GSM2772132 | Right | Right hemicolectomy | 6.545428301 |
| GSM2772137 | Right | Ext right hemicolectomy | 6.493482935 |
| GSM2772142 | Right | Right hemicolectomy | 6.351460417 |
| GSM2772143 | Right | Extended right hemicolectomy | 5.80107185 |
| GSM2772145 | Right | Right Hemicolectomy | 6.28550086 |
| GSM2772147 | Right | Colectomy | 6.425400439 |
| GSM2772149 | Right | Right Hemicolectomy | 6.61133169 |
| GSM2772150 | Right | colectomy | 6.50904978 |
| GSM2772152 | Right | Right Hemicolectomy | 6.943166209 |
| GSM2772157 | Right | Right Hemicolectomy | 6.554193956 |
| GSM2772158 | Right | Right Hemicolectomy | 5.838393195 |
| GSM2772161 | Right | Right Hemicolectomy | 7.217665746 |
| GSM2772163 | Right | Right Hemicolectomy | 6.516690781 |
| GSM2772166 | Right | Right Hemicolectomy | 6.547585776 |
| GSM2772170 | Right | Right Hemicolectomy | 5.039425016 |
| GSM2772172 | Right | Right Hemicolectomy | 6.798579041 |
| GSM2772173 | Right | Right Hemicolectomy | 6.558384021 |
| GSM2772176 | Right | Right Hemicolectomy | 7.208753179 |
| GSM2772179 | Right | Right Hemicolectomy | 7.076563368 |
| GSM2772184 | Right | Right hemicolectomy | 6.804725504 |
| GSM2772187 | Right | Laparoscopic sub-total colectomy | 6.179188119 |
| GSM2772190 | Right | Laparoscopic right hemicolectomy | 6.443394257 |
| GSM2772191 | Right | Open right hemicolectomy | 6.758412261 |
| GSM2772193 | Right | Open right hemicolectomy | 6.883058466 |
| GSM2772195 | Right | Open transverse colectomy | 6.317942664 |
| GSM2772198 | Right | Open right hemicolectomy | 6.401218574 |
| GSM2772199 | Right | Open right hemicolectomy | 6.804664121 |
| GSM2772203 | Right | Laparoscopic right hemicolectomy | 6.414590339 |
| GSM2772206 | Right | Right hemicolectomy | 7.426340791 |
| GSM2772214 | Right | right hemicolectomy | 6.774570843 |
| GSM2772217 | Right | subtotal colectomy (left right) | 6.481609404 |
| GSM2772218 | Right | Right Hemicolectomy | 6.753120678 |
| GSM2772220 | Right | Right Hemicolectomy | 6.252604692 |
| GSM2772221 | Right | Right Hemicolectomy | 6.616682846 |
| GSM2772225 | Right | Right Hemicolectomy | 7.03886817 |
| GSM2772227 | Right | Right Hemicolectomy | 7.065915146 |
| GSM2772229 | Right | Right Hemicolectomy | 7.098374997 |
| GSM2772230 | Right | Right Hemicolectomy | 6.63315083 |
| GSM2772235 | Right | Right Hemicolectomy | 6.585066057 |
| GSM2772236 | Right | Right Hemicolectomy | 6.905153993 |
| GSM2772237 | Right | Right Hemicolectomy | 6.222467742 |
| GSM2772239 | Right | Right Hemicolectomy | 5.985965149 |
| GSM2772240 | Right | Right Hemicolectomy | 7.701395782 |
| GSM2772243 | Right | Open right hemicolectomy | 6.792909907 |
| GSM2772245 | Right | Laparoscopic right hemicolectomy | 6.948968528 |
| GSM2772255 | Right | Laparoscopic right hemicolectomy | 6.093077125 |
| GSM2772257 | Right | Laparoscopic extended right hemicolectomy | 7.141476613 |
| GSM2772261 | Right | Laparoscopic right hemicolectomy | 6.655680042 |
| GSM2772263 | Right | Open right hemicolectomy | 6.000477466 |
| GSM2772264 | Right | Open right hemicolectomy | 6.386032324 |
| GSM2772265 | Right | Laparoscopic assisted right hemicolectomy | 7.367147757 |
| GSM2772266 | Right | Laparoscopic right hemicolectomy | 6.551154251 |
| GSM2772267 | Right | Laparoscopic assisted right hemicolectomy | 6.90267732 |
| GSM2772268 | Right | Laparoscopic right hemicolectomy | 6.110810152 |
| GSM2772272 | Right | Right hemicolectomy | 6.876038862 |
| GSM2772274 | Right | Right Hemicolectomy | 6.330903374 |
| GSM2772276 | Right | Right Hemicolectomy | 6.796039432 |
| GSM2772181 | Right | colon resection | 7.043436018 |
| GSM2772210 | Right | Intestinal resection | 7.031049096 |
| GSM2772211 | Right | Intestinal resection | 7.531199249 |
| GSM2772212 | Right | intestinal resection | 7.128639143 |

**Table S4.** Patient characteristics and ACSL5 expression in primary colorectal tumors from the GSE40967 dataset (related to Figure 9D).

| Variable | | | | | |
| --- | --- | --- | --- | --- | --- |
| geo_accession | chemotherapy | death-yes 1, no 0 | survival time (months) | mmr.status | ACSL5 mRNA expression (log2 normalized intensity signal) |
| GSM1681353 | N | 0 | 131 | pMMR | 10.41180017 |
| GSM1681354 | N | 0 | 142 | pMMR | 10.35869176 |
| GSM1681355 | Y | 0 | 141 | pMMR | 10.29866706 |
| GSM1681356 | N | 0 | 134 | pMMR | 9.447959274 |
| GSM1681357 | N | 0 | 123 | pMMR | 10.59360823 |
| GSM1681359 | N | 1 | 36 | pMMR | 10.31877711 |
| GSM1681360 | N | 1 | 30 | dMMR | 10.46606265 |
| GSM1681361 | N | 0 | 86 | pMMR | 10.13162348 |
| GSM1681362 | N | 0 | 86 | pMMR | 10.06398737 |
| GSM1681363 | Y | 0 | 85 | dMMR | 10.33721337 |
| GSM1681364 | Y | 0 | 85 | pMMR | 10.56737304 |
| GSM1681365 | Y | 0 | 86 | dMMR | 11.07973836 |
| GSM1681367 | Y | 0 | 76 | pMMR | 10.46772479 |
| GSM1681368 | Y | 0 | 81 | pMMR | 10.14233362 |
| GSM1681369 | Y | 1 | 65 | pMMR | 9.557862774 |
| GSM1681370 | N | 0 | 74 | pMMR | 10.4275351 |
| GSM1681371 | N | 0 | 68 | dMMR | 9.937721925 |
| GSM971957 | N | 1 | 10 | dMMR | 10.77511316 |
| GSM971958 | N | 1 | 9 | pMMR | 8.409567148 |
| GSM971959 | N | 0 | 52 | pMMR | 9.539639312 |
| GSM971960 | N | 0 | 74 | pMMR | 11.16973815 |
| GSM971961 | Y | 1 | 32 | pMMR | 8.738464613 |
| GSM971962 | Y | 0 | 73 | pMMR | 8.327938677 |
| GSM971963 | Y | 1 | 20 | pMMR | 8.526861351 |
| GSM971964 | N | 0 | 35 | pMMR | 10.22605759 |
| GSM971965 | N | 0 | 33 | pMMR | 9.70933689 |
| GSM971966 | N | 0 | 63 | N/A | 9.706082245 |
| GSM971967 | N | 1 | 31 | pMMR | 8.591653561 |
| GSM971968 | N | 0 | 112 | pMMR | 9.787999565 |
| GSM971969 | N | 0 | 63 | dMMR | 10.22699966 |
| GSM971970 | N | 0 | 86 | dMMR | 9.212336714 |
| GSM971971 | N | 0 | 65 | dMMR | 9.964076859 |
| GSM971972 | N | 0 | 75 | dMMR | 10.64928832 |
| GSM971973 | N | 0 | 46 | dMMR | 10.42628818 |
| GSM971974 | N | 0 | 56 | pMMR | 9.605001816 |
| GSM971975 | N | 0 | 46 | pMMR | 10.4797573 |
| GSM971976 | N | 0 | 36 | N/A | 11.00081673 |
| GSM971977 | N | 0 | 49 | dMMR | 9.328927456 |
| GSM971978 | N | 0 | 109 | dMMR | 10.36684632 |
| GSM971979 | N | 0 | 53 | pMMR | 7.461875465 |
| GSM971980 | N | 0 | 59 | dMMR | 9.914504509 |
| GSM971981 | N | 0 | 48 | pMMR | 8.720020077 |
| GSM971982 | N | 1 | 42 | dMMR | 9.297503427 |
| GSM971983 | N | 0 | 87 | dMMR | 9.685394635 |
| GSM971984 | N | 0 | 81 | dMMR | 9.785320117 |
| GSM971985 | N | 0 | 7 | dMMR | 10.29259405 |
| GSM971986 | N | 0 | 68 | pMMR | 9.898243761 |
| GSM971987 | N | 0 | 68 | pMMR | 10.23149265 |
| GSM971988 | N | 1 | 18 | pMMR | 10.52786078 |
| GSM971989 | N | 1 | 24 | pMMR | 10.46908128 |
| GSM971990 | N | 1 | 12 | pMMR | 9.930046676 |
| GSM971991 | N | 1 | 55 | dMMR | 10.95849876 |
| GSM971992 | N | 1 | 20 | pMMR | 10.54067135 |
| GSM971993 | N | 0 | 56 | pMMR | 9.699686006 |
| GSM971994 | N | 0 | 43 | dMMR | 11.05557679 |
| GSM971995 | N | 0 | 25 | pMMR | 9.919023852 |
| GSM971996 | N | 0 | 76 | pMMR | 8.925838701 |
| GSM971997 | N | 0 | 89 | pMMR | 8.903470395 |
| GSM971998 | N | 1 | 23 | pMMR | 10.35223658 |
| GSM971999 | N | 0 | 1 | pMMR | 9.824362344 |
| GSM972000 | Y | 0 | 9 | dMMR | 10.66312668 |
| GSM972001 | N | 0 | 73 | pMMR | 11.04388706 |
| GSM972002 | N | 0 | 26 | pMMR | 9.362421186 |
| GSM972003 | Y | 1 | 27 | dMMR | 10.4276301 |
| GSM972004 | N | 0 | 89 | pMMR | 9.634325047 |
| GSM972005 | N | 0 | 44 | pMMR | 9.376268383 |
| GSM972006 | Y | 1 | 23 | pMMR | 9.950506667 |
| GSM972007 | N | 1 | 2 | pMMR | 9.159359305 |
| GSM972008 | N | 1 | 72 | pMMR | 9.239866351 |
| GSM972009 | N | 1 | 10 | pMMR | 9.900665012 |
| GSM972010 | N | 1 | 46 | pMMR | 10.3176037 |
| GSM972011 | N | 1 | 20 | pMMR | 8.153424925 |
| GSM972012 | N | 0 | 101 | pMMR | 10.38439977 |
| GSM972013 | N | 1 | 43 | dMMR | 10.92922398 |
| GSM972014 | Y | 1 | 29 | pMMR | 10.25458294 |
| GSM972015 | N | 0 | 91 | dMMR | 9.347909424 |
| GSM972016 | N | 1 | 16 | pMMR | 8.820750674 |
| GSM972017 | Y | 0 | 43 | pMMR | 10.01482573 |
| GSM972018 | Y | 0 | 117 | pMMR | 10.27175876 |
| GSM972019 | Y | 1 | 25 | pMMR | 9.867058478 |
| GSM972020 | Y | 0 | 78 | dMMR | 10.78465415 |
| GSM972021 | Y | 1 | 37 | pMMR | 8.882867453 |
| GSM972022 | Y | 0 | 82 | pMMR | 9.680888647 |
| GSM972023 | Y | 0 | 16 | pMMR | 10.19638576 |
| GSM972024 | Y | 0 | 43 | pMMR | 10.32371973 |
| GSM972025 | Y | 0 | 53 | dMMR | 10.86037304 |
| GSM972026 | Y | 1 | 34 | pMMR | 9.080930684 |
| GSM972027 | Y | 0 | 94 | dMMR | 10.79062156 |
| GSM972028 | Y | 0 | 78 | pMMR | 10.39680724 |
| GSM972029 | Y | 0 | 36 | pMMR | 10.06563923 |
| GSM972030 | Y | 0 | 52 | pMMR | 8.317942188 |
| GSM972031 | N | 0 | 55 | pMMR | 9.717269796 |
| GSM972032 | N | 0 | 60 | pMMR | 10.23248268 |
| GSM972033 | N | 0 | 50 | pMMR | 10.88132675 |
| GSM972034 | N | 0 | 50 | pMMR | 10.52047364 |
| GSM972035 | N | 0 | 39 | pMMR | 10.02111291 |
| GSM972036 | N | 0 | 50 | pMMR | 9.797803487 |
| GSM972037 | N | 0 | 33 | pMMR | 10.18597641 |
| GSM972038 | N | 1 | 1 | pMMR | 10.39641851 |
| GSM972039 | Y | 1 | 52 | pMMR | 7.196333529 |
| GSM972040 | N | 0 | 63 | dMMR | 11.19323433 |
| GSM972041 | N | 0 | 55 | pMMR | 10.17757133 |
| GSM972042 | N | 0 | 51 | pMMR | 10.41571662 |
| GSM972043 | Y | 1 | 27 | pMMR | 10.27709542 |
| GSM972044 | Y | 0 | 42 | pMMR | 9.093672605 |
| GSM972045 | Y | 1 | 11 | pMMR | 10.09197286 |
| GSM972046 | N | 0 | 58 | pMMR | 9.298505394 |
| GSM972047 | N | 0 | 33 | pMMR | 9.353136048 |
| GSM972048 | Y | 0 | 30 | pMMR | 10.28751272 |
| GSM972049 | N | 0 | 26 | pMMR | 8.318112855 |
| GSM972050 | N | 0 | 19 | pMMR | 10.64617938 |
| GSM972051 | Y | 1 | 28 | pMMR | 9.238153292 |
| GSM972052 | N | 0 | 15 | pMMR | 10.10415355 |
| GSM972053 | N | 0 | 29 | pMMR | 10.34274622 |
| GSM972054 | N | 0 | 26 | pMMR | 9.438879488 |
| GSM972055 | N | 0 | 93 | N/A | 10.28558987 |
| GSM972056 | N | 0 | 92 | N/A | 9.453252546 |
| GSM972057 | N | 1 | 26 | N/A | 9.846141332 |
| GSM972058 | N | 0 | 60 | N/A | 8.160149811 |
| GSM972059 | N | 0 | 59 | N/A | 9.484808489 |
| GSM972060 | N | 0 | 82 | N/A | 10.34521835 |
| GSM972061 | N | 1 | 43 | N/A | 10.4168203 |
| GSM972062 | Y | 1 | 23 | pMMR | 10.0333033 |
| GSM972063 | N | 0 | 70 | N/A | 10.27437976 |
| GSM972064 | Y | 1 | 22 | dMMR | 10.66458765 |
| GSM972065 | Y | 0 | 46 | N/A | 9.933309125 |
| GSM972066 | Y | 0 | 49 | N/A | 10.04486037 |
| GSM972067 | N | 1 | 183 | N/A | 10.2919647 |
| GSM972068 | Y | 1 | 22 | N/A | 9.369680679 |
| GSM972069 | N | 0 | 201 | N/A | 10.42796406 |
| GSM972070 | Y | 1 | 68 | dMMR | 10.53491776 |
| GSM972071 | N | 0 | 183 | N/A | 10.45142184 |
| GSM972072 | N | 0 | 172 | N/A | 9.313506293 |
| GSM972073 | N | 0 | 163 | N/A | 9.888997743 |
| GSM972074 | Y | 1 | 75 | N/A | 10.83280113 |
| GSM972075 | N | 1 | 44 | N/A | 9.918835407 |
| GSM972076 | N | 0 | 153 | N/A | 8.914058883 |
| GSM972077 | N | 1 | 31 | N/A | 10.01008214 |
| GSM972078 | Y | 1 | 145 | N/A | 10.70093111 |
| GSM972079 | Y | 0 | 168 | N/A | 9.659282059 |
| GSM972080 | Y | 0 | 165 | N/A | 9.00326085 |
| GSM972081 | N | 0 | 26 | N/A | 10.65664294 |
| GSM972082 | Y | 1 | 132 | N/A | 8.846581432 |
| GSM972083 | N | 0 | 127 | N/A | 10.21565907 |
| GSM972084 | N | 0 | 129 | N/A | 9.714156103 |
| GSM972085 | N | 0 | 97 | N/A | 10.53531226 |
| GSM972086 | N | 1 | 43 | pMMR | 10.13345566 |
| GSM972087 | N | 1 | 32 | pMMR | 10.12956073 |
| GSM972088 | N | 0 | 79 | pMMR | 9.455796999 |
| GSM972089 | N | 0 | 87 | pMMR | 8.215577234 |
| GSM972090 | N | 0 | 69 | pMMR | 10.80255275 |
| GSM972091 | N | 0 | 70 | pMMR | 10.89432449 |
| GSM972092 | N | 0 | 47 | pMMR | 10.01574618 |
| GSM972093 | Y | 0 | 74 | pMMR | 10.25654024 |
| GSM972094 | N | 0 | 61 | pMMR | 9.898893694 |
| GSM972095 | N | 0 | 67 | pMMR | 9.501382686 |
| GSM972096 | Y | 0 | 15 | pMMR | 10.09892769 |
| GSM972097 | Y | 1 | 12 | pMMR | 8.623905819 |
| GSM972098 | Y | 1 | 15 | pMMR | 9.124764642 |
| GSM972099 | Y | 0 | 15 | dMMR | 11.14688634 |
| GSM972100 | Y | 0 | 25 | pMMR | 9.073597822 |
| GSM972101 | Y | 0 | 35 | pMMR | 9.332452811 |
| GSM972102 | Y | 0 | 46 | pMMR | 9.988474761 |
| GSM972103 | Y | 0 | 58 | pMMR | 10.24698144 |
| GSM972104 | Y | 0 | 41 | pMMR | 9.239496563 |
| GSM972105 | Y | 0 | 32 | pMMR | 10.63179945 |
| GSM972106 | Y | 0 | 72 | pMMR | 10.84113016 |
| GSM972107 | Y | 1 | 60 | pMMR | 10.12304114 |
| GSM972108 | Y | 0 | 103 | pMMR | 10.47238792 |
| GSM972109 | Y | 0 | 36 | pMMR | 10.09195273 |
| GSM972110 | Y | 1 | 7 | pMMR | 10.29488206 |
| GSM972111 | Y | 0 | 22 | pMMR | 9.845387554 |
| GSM972112 | Y | 1 | 32 | pMMR | 8.2580444 |
| GSM972113 | Y | 0 | 27 | pMMR | 9.829058566 |
| GSM972114 | Y | 0 | 46 | pMMR | 10.15750979 |
| GSM972115 | Y | 1 | 75 | pMMR | 10.4017895 |
| GSM972116 | Y | 1 | 29 | pMMR | 9.936581847 |
| GSM972117 | Y | 0 | 32 | N/A | 10.21360981 |
| GSM972118 | Y | 0 | 30 | pMMR | 9.809980064 |
| GSM972119 | Y | 0 | 37 | pMMR | 10.88609323 |
| GSM972120 | Y | 0 | 36 | pMMR | 9.650643773 |
| GSM972121 | Y | 0 | 35 | pMMR | 9.317614262 |
| GSM972122 | Y | 1 | 19 | dMMR | 10.69394904 |
| GSM972123 | N | 0 | 71 | pMMR | 10.35208057 |
| GSM972124 | Y | 0 | 75 | pMMR | 10.50365181 |
| GSM972125 | Y | 0 | 68 | pMMR | 9.940211119 |
| GSM972126 | N | 0 | 63 | pMMR | 10.39519811 |
| GSM972127 | N | 0 | 85 | pMMR | 10.10826292 |
| GSM972128 | Y | 0 | 93 | pMMR | 10.12755014 |
| GSM972129 | N | 0 | 11 | pMMR | 9.155680356 |
| GSM972130 | N | 0 | 55 | dMMR | 11.13953163 |
| GSM972131 | N | 0 | 86 | pMMR | 10.30941874 |
| GSM972132 | N | 1 | 61 | pMMR | 9.733495816 |
| GSM972133 | Y | 1 | 53 | pMMR | 9.684107977 |
| GSM972134 | N | 1 | 14 | pMMR | 10.12210194 |
| GSM972135 | N | 1 | 13 | pMMR | 9.62476611 |
| GSM972136 | Y | 0 | 130 | pMMR | 10.25855226 |
| GSM972137 | Y | 0 | 129 | pMMR | 10.67389341 |
| GSM972138 | N | 1 | 53 | pMMR | 9.759929604 |
| GSM972139 | Y | 0 | 102 | pMMR | 10.95708939 |
| GSM972140 | N | 0 | 172 | pMMR | 9.717316953 |
| GSM972141 | Y | 0 | 84 | pMMR | 10.16036086 |
| GSM972142 | N | 1 | 35 | pMMR | 10.65926107 |
| GSM972143 | N | 0 | 154 | pMMR | 10.25484997 |
| GSM972144 | N | 0 | 121 | pMMR | 10.77543364 |
| GSM972145 | Y | 0 | 135 | pMMR | 10.49799462 |
| GSM972146 | Y | 0 | 66 | pMMR | 9.161899314 |
| GSM972147 | N | 0 | 140 | pMMR | 10.53955036 |
| GSM972148 | Y | 0 | 127 | pMMR | 10.77745301 |
| GSM972149 | N | 0 | 132 | pMMR | 10.45064725 |
| GSM972150 | Y | 0 | 109 | pMMR | 10.72597946 |
| GSM972151 | Y | 0 | 77 | pMMR | 10.46495964 |
| GSM972152 | Y | 0 | 56 | pMMR | 10.92365549 |
| GSM972153 | N | 1 | 66 | pMMR | 9.840094147 |
| GSM972154 | Y | 1 | 44 | pMMR | 9.901676684 |
| GSM972155 | Y | 1 | 24 | pMMR | 10.28542327 |
| GSM972156 | Y | 1 | 112 | pMMR | 10.43318575 |
| GSM972157 | Y | 0 | 118 | pMMR | 10.14202714 |
| GSM972158 | Y | 0 | 120 | pMMR | 9.811357691 |
| GSM972159 | Y | 0 | 93 | pMMR | 9.922792214 |
| GSM972160 | N | 0 | 77 | pMMR | 10.52189299 |
| GSM972161 | N | 1 | 11 | pMMR | 9.984832197 |
| GSM972162 | Y | 0 | 77 | pMMR | 9.615375265 |
| GSM972163 | Y | 0 | 41 | pMMR | 9.931541802 |
| GSM972164 | Y | 1 | 69 | dMMR | 10.74533182 |
| GSM972165 | N | 1 | 87 | pMMR | 10.36950854 |
| GSM972166 | Y | 1 | 12 | pMMR | 10.62246951 |
| GSM972167 | N | 1 | 45 | pMMR | 10.57743172 |
| GSM972168 | N | 1 | 8 | pMMR | 9.319690188 |
| GSM972169 | N | 0 | 164 | pMMR | 11.07269377 |
| GSM972170 | N | 0 | 73 | pMMR | 10.05980087 |
| GSM972171 | N | 0 | 130 | pMMR | 8.362189471 |
| GSM972172 | N | 1 | 5 | dMMR | 11.21644428 |
| GSM972173 | Y | 1 | 26 | pMMR | 10.55917012 |
| GSM972174 | Y | 0 | 97 | pMMR | 10.26016238 |
| GSM972175 | N | 1 | 43 | pMMR | 9.589148091 |
| GSM972176 | N | 1 | 36 | pMMR | 10.06625173 |
| GSM972177 | N | 1 | 22 | pMMR | 10.46127917 |
| GSM972178 | Y | 0 | 67 | pMMR | 10.01044781 |
| GSM972179 | N | 1 | 18 | pMMR | 10.17310637 |
| GSM972180 | Y | 1 | 24 | pMMR | 8.770461791 |
| GSM972181 | Y | 1 | 34 | pMMR | 7.952625668 |
| GSM972182 | N | 1 | 58 | pMMR | 10.00737965 |
| GSM972183 | Y | 1 | 32 | pMMR | 8.692872425 |
| GSM972184 | N | 1 | 39 | pMMR | 7.583701379 |
| GSM972185 | Y | 0 | 39 | pMMR | 10.15391653 |
| GSM972186 | Y | 1 | 47 | pMMR | 8.570417967 |
| GSM972187 | N | 0 | 49 | pMMR | 9.434281611 |
| GSM972188 | Y | 0 | 22 | pMMR | 10.3478643 |
| GSM972189 | N | 0 | 192 | pMMR | 10.54968161 |
| GSM972190 | Y | 0 | 108 | pMMR | 10.25428425 |
| GSM972191 | Y | 0 | 122 | pMMR | 9.947946604 |
| GSM972192 | N | 0 | 158 | pMMR | 10.74589101 |
| GSM972193 | Y | 0 | 73 | dMMR | 10.93404431 |
| GSM972194 | Y | 0 | 83 | pMMR | 9.693713822 |
| GSM972195 | Y | 0 | 120 | pMMR | 10.08373034 |
| GSM972196 | N | 0 | 73 | pMMR | 8.723342792 |
| GSM972197 | N | 0 | 50 | pMMR | 9.576180317 |
| GSM972198 | Y | 0 | 57 | dMMR | 10.68187835 |
| GSM972199 | Y | 0 | 66 | pMMR | 10.21715924 |
| GSM972200 | N | 0 | 54 | dMMR | 11.39018192 |
| GSM972201 | Y | 0 | 67 | pMMR | 10.22806633 |
| GSM972202 | Y | 0 | 53 | pMMR | 9.790972027 |
| GSM972203 | Y | 0 | 54 | pMMR | 10.01774011 |
| GSM972204 | Y | 0 | 44 | pMMR | 9.512669326 |
| GSM972205 | N | 1 | 35 | pMMR | 10.41729266 |
| GSM972206 | N | 0 | 42 | pMMR | 10.11455376 |
| GSM972207 | Y | 0 | 82 | pMMR | 9.843282869 |
| GSM972208 | N/A | 1 | 25 | pMMR | 9.998587766 |
| GSM972209 | N/A | 1 | 13 | pMMR | 8.581971709 |
| GSM972210 | N/A | 1 | 46 | pMMR | 9.917982719 |
| GSM972211 | Y | 0 | 57 | pMMR | 10.79623999 |
| GSM972212 | N/A | 1 | 18 | pMMR | 10.17888289 |
| GSM972213 | N/A | 1 | 1 | pMMR | 8.331469244 |
| GSM972214 | N/A | 1 | 2 | pMMR | 8.966961872 |
| GSM972215 | Y | 0 | 47 | pMMR | 9.708133942 |
| GSM972216 | N/A | 0 | 94 | pMMR | 10.61896115 |
| GSM972217 | N/A | 0 | 70 | pMMR | 10.22308117 |
| GSM972218 | N/A | 1 | 7 | pMMR | 9.723755369 |
| GSM972219 | N/A | 0 | 53 | pMMR | 9.853013265 |
| GSM972220 | N | 0 | 1 | dMMR | 9.561736588 |
| GSM972221 | N | 0 | 65 | dMMR | 9.070698941 |
| GSM972222 | N | 0 | 57 | dMMR | 9.470653277 |
| GSM972223 | N | 1 | 33 | dMMR | 9.0460937 |
| GSM972224 | Y | 1 | 25 | pMMR | 8.856470862 |
| GSM972225 | Y | 0 | 37 | pMMR | 10.09192931 |
| GSM972226 | N | 0 | 20 | pMMR | 8.927085009 |
| GSM972227 | Y | 1 | 20 | pMMR | 10.2095465 |
| GSM972228 | N | 0 | 1 | pMMR | 10.18643976 |
| GSM972229 | N | 0 | 0 | dMMR | 9.569297693 |
| GSM972230 | Y | 0 | 27 | pMMR | 10.209199 |
| GSM972231 | Y | 0 | 14 | pMMR | 10.12152821 |
| GSM972232 | Y | 0 | 21 | pMMR | 9.929559332 |
| GSM972233 | N | 0 | 15 | pMMR | 10.60572503 |
| GSM972234 | Y | 1 | 10 | pMMR | 9.688396412 |
| GSM972235 | Y | 0 | 7 | pMMR | 9.395254775 |
| GSM972236 | Y | 0 | 12 | pMMR | 10.6501598 |
| GSM972237 | N | 0 | 25 | pMMR | 10.49527202 |
| GSM972238 | Y | 0 | 19 | pMMR | 9.67747874 |
| GSM972239 | N | 0 | 14 | pMMR | 10.33308411 |
| GSM972240 | Y | 0 | 14 | pMMR | 7.94463639 |
| GSM972241 | N | 1 | 17 | dMMR | 10.23810469 |
| GSM972242 | N | 0 | 17 | pMMR | 10.61398481 |
| GSM972243 | N | 0 | 2 | pMMR | 10.67806978 |
| GSM972244 | N | 0 | 14 | pMMR | 9.671726631 |
| GSM972245 | N | 1 | 0 | pMMR | 10.22144116 |
| GSM972246 | N | 0 | 0 | pMMR | 10.43060582 |
| GSM972247 | N | 0 | 6 | pMMR | 10.07435319 |
| GSM972248 | N | 0 | 14 | pMMR | 10.07356092 |
| GSM972249 | N | 0 | 14 | pMMR | 10.39281572 |
| GSM972250 | Y | 0 | 12 | pMMR | 10.4482089 |
| GSM972251 | Y | 1 | 7 | pMMR | 10.23786065 |
| GSM972252 | Y | 0 | 16 | pMMR | 10.06127959 |
| GSM972253 | Y | 0 | 16 | pMMR | 10.78380884 |
| GSM972254 | Y | 0 | 14 | dMMR | 10.5689442 |
| GSM972255 | N | 0 | 5 | pMMR | 10.3678931 |
| GSM972256 | Y | 0 | 19 | pMMR | 9.395858402 |
| GSM972257 | N | 1 | 21 | pMMR | 9.465779575 |
| GSM972258 | N | 1 | 0 | pMMR | 10.37227902 |
| GSM972259 | Y | 0 | 29 | pMMR | 8.865976171 |
| GSM972260 | Y | 0 | 13 | pMMR | 10.20479624 |
| GSM972261 | N | 1 | 7 | dMMR | 9.310218629 |
| GSM972262 | N/A | 0 | 5 | pMMR | 7.911336343 |
| GSM972263 | Y | 0 | 76 | dMMR | 10.31546884 |
| GSM972264 | N | 0 | 73 | dMMR | 9.604483717 |
| GSM972265 | Y | 0 | 60 | pMMR | 8.085968268 |
| GSM972266 | Y | 1 | 20 | dMMR | 9.987274872 |
| GSM972267 | N | 1 | 67 | pMMR | 10.53634064 |
| GSM972268 | Y | 0 | 48 | pMMR | 10.13734508 |
| GSM972269 | N | 0 | 79 | pMMR | 9.126763245 |
| GSM972270 | N | 0 | 65 | pMMR | 8.569141991 |
| GSM972271 | N | 1 | 14 | pMMR | 9.392745591 |
| GSM972272 | Y | 1 | 15 | pMMR | 10.2823566 |
| GSM972273 | N | 1 | 16 | pMMR | 10.41684058 |
| GSM972274 | N | 0 | 57 | dMMR | 9.675287068 |
| GSM972275 | N | 1 | 4 | pMMR | 7.889792211 |
| GSM972276 | Y | 0 | 43 | dMMR | 9.273093054 |
| GSM972277 | N | 0 | 51 | pMMR | 7.51804875 |
| GSM972278 | N | 0 | 45 | dMMR | 9.765561518 |
| GSM972279 | Y | 0 | 52 | dMMR | 9.521585033 |
| GSM972280 | N | 1 | 46 | pMMR | 7.740628106 |
| GSM972281 | N | 0 | 56 | pMMR | 10.30342927 |
| GSM972282 | N | 0 | 51 | dMMR | 10.37072237 |
| GSM972283 | N | 0 | 51 | pMMR | 8.77180648 |
| GSM972284 | Y | 1 | 27 | pMMR | 10.24749551 |
| GSM972285 | N | 1 | 2 | pMMR | 8.335799934 |
| GSM972286 | N | 0 | 37 | dMMR | 9.747413951 |
| GSM972287 | N | 0 | 43 | dMMR | 8.267140598 |
| GSM972288 | Y | 1 | 35 | pMMR | 10.77979048 |
| GSM972289 | Y | 1 | 17 | pMMR | 10.3400422 |
| GSM972290 | N | 0 | 39 | dMMR | 10.50955576 |
| GSM972291 | Y | 0 | 32 | dMMR | 9.685031204 |
| GSM972292 | N | 0 | 25 | pMMR | 8.867127746 |
| GSM972293 | N | 0 | 50 | pMMR | 9.653239762 |
| GSM972294 | N | 0 | 25 | dMMR | 8.329577504 |
| GSM972295 | Y | 0 | 61 | pMMR | 9.565217101 |
| GSM972296 | N | 0 | 22 | dMMR | 10.80543668 |
| GSM972297 | N | 1 | 46 | pMMR | 9.552244619 |
| GSM972298 | N | 0 | 9 | dMMR | 8.998040564 |
| GSM972299 | Y | 1 | 41 | pMMR | 10.31324352 |
| GSM972300 | N | 0 | 41 | pMMR | 9.282318618 |
| GSM972301 | N | 1 | 105 | pMMR | 9.987525615 |
| GSM972302 | N | 1 | 27 | pMMR | 9.906291144 |
| GSM972303 | N | 0 | 19 | pMMR | 10.46427327 |
| GSM972304 | Y | 0 | 30 | pMMR | 10.47870318 |
| GSM972305 | N | 0 | 31 | dMMR | 9.660849412 |
| GSM972306 | Y | 0 | 56 | pMMR | 10.22543144 |
| GSM972307 | Y | 0 | 82 | pMMR | 10.26505162 |
| GSM972308 | N | 0 | 92 | pMMR | 9.464704913 |
| GSM972309 | Y | 0 | 95 | pMMR | 9.981215663 |
| GSM972310 | N | 0 | 25 | pMMR | 9.271024634 |
| GSM972311 | Y | 0 | 106 | pMMR | 8.007438392 |
| GSM972312 | N | 1 | 97 | pMMR | 10.24629587 |
| GSM972313 | N | 1 | 91 | pMMR | 10.14981401 |
| GSM972314 | N | 0 | 46 | dMMR | 8.875874922 |
| GSM972315 | N | 0 | 84 | dMMR | 10.76632856 |
| GSM972316 | N | 0 | 83 | dMMR | 8.825323109 |
| GSM972317 | N | 1 | 30 | dMMR | 9.15095631 |
| GSM972318 | N | 1 | 36 | pMMR | 9.635746901 |
| GSM972319 | Y | 1 | 78 | pMMR | 9.919544239 |
| GSM972320 | N | 1 | 58 | pMMR | 9.786189186 |
| GSM972321 | N | 0 | 9 | pMMR | 9.74827153 |
| GSM972322 | Y | 1 | 86 | pMMR | 10.29280478 |
| GSM972323 | Y | 0 | 80 | pMMR | 10.18186426 |
| GSM972324 | N | 0 | 82 | pMMR | 10.6092979 |
| GSM972325 | N | 0 | 71 | pMMR | 9.802390149 |
| GSM972326 | Y | 0 | 85 | pMMR | 10.19170338 |
| GSM972327 | N | 0 | 83 | pMMR | 9.868084528 |
| GSM972328 | Y | 0 | 82 | pMMR | 10.11348891 |
| GSM972329 | N | 0 | 78 | pMMR | 10.61568959 |
| GSM972330 | N | 0 | 86 | dMMR | 10.07727544 |
| GSM972331 | N | 1 | 75 | pMMR | 10.5907574 |
| GSM972332 | N | 0 | 87 | pMMR | 8.654251647 |
| GSM972333 | Y | 0 | 80 | pMMR | 10.72556537 |
| GSM972334 | Y | 0 | 77 | dMMR | 9.216885356 |
| GSM972335 | N | 1 | 46 | pMMR | 10.24756754 |
| GSM972336 | Y | 0 | 50 | pMMR | 10.05559629 |
| GSM972337 | N | 1 | 41 | dMMR | 9.997037887 |
| GSM972338 | Y | 0 | 74 | pMMR | 10.83533373 |
| GSM972339 | Y | 0 | 76 | pMMR | 9.610996298 |
| GSM972340 | N | 0 | 84 | pMMR | 11.06819501 |
| GSM972341 | N | 0 | 69 | pMMR | 9.2214846 |
| GSM972342 | N | 0 | 28 | pMMR | 10.86931182 |
| GSM972343 | N | 1 | 52 | pMMR | 10.25882385 |
| GSM972344 | N | 0 | 75 | pMMR | 9.208575841 |
| GSM972345 | N | 1 | 42 | pMMR | 10.68691351 |
| GSM972346 | N | 1 | 72 | pMMR | 8.414992983 |
| GSM972347 | N | 0 | 7 | pMMR | 10.22803971 |
| GSM972348 | Y | 0 | 62 | pMMR | 9.88578296 |
| GSM972349 | Y | 1 | 5 | pMMR | 8.329326479 |
| GSM972350 | N | 1 | 9 | pMMR | 10.46125439 |
| GSM972351 | N | 0 | 24 | N/A | 10.6543271 |
| GSM972352 | Y | 0 | 139 | pMMR | 9.803125818 |
| GSM972353 | Y | 0 | 133 | dMMR | 9.312414274 |
| GSM972354 | N | 1 | 95 | pMMR | 8.819134956 |
| GSM972355 | Y | 1 | 34 | pMMR | 9.892839975 |
| GSM972356 | N | 1 | 53 | pMMR | 10.05355135 |
| GSM972357 | N | 1 | 42 | pMMR | 10.67046792 |
| GSM972358 | Y | 0 | 81 | pMMR | 10.04849123 |
| GSM972359 | Y | 1 | 30 | pMMR | 9.135687022 |
| GSM972360 | Y | 0 | 141 | pMMR | 10.70446451 |
| GSM972361 | Y | 1 | 19 | pMMR | 9.500184108 |
| GSM972362 | Y | 0 | 98 | pMMR | 10.07664021 |
| GSM972363 | Y | 0 | 140 | pMMR | 10.77109524 |
| GSM972364 | Y | 0 | 103 | pMMR | 7.764077588 |
| GSM972365 | Y | 0 | 47 | pMMR | 9.095913051 |
| GSM972366 | Y | 1 | 24 | pMMR | 9.870693714 |
| GSM972367 | Y | 0 | 95 | pMMR | 9.67399111 |
| GSM972368 | Y | 0 | 92 | pMMR | 10.42332069 |
| GSM972369 | Y | 1 | 102 | pMMR | 9.604881745 |
| GSM972370 | Y | 1 | 71 | pMMR | 10.59214488 |
| GSM972371 | Y | 0 | 81 | pMMR | 10.75967048 |
| GSM972372 | Y | 1 | 50 | pMMR | 10.43556838 |
| GSM972373 | Y | 0 | 79 | pMMR | 11.12076816 |
| GSM972374 | Y | 0 | 90 | pMMR | 10.87534226 |
| GSM972375 | Y | 0 | 117 | pMMR | 10.1838461 |
| GSM972376 | Y | 0 | 87 | pMMR | 10.13347247 |
| GSM972377 | Y | 0 | 82 | pMMR | 10.2264358 |
| GSM972378 | Y | 0 | 86 | pMMR | 9.851199258 |
| GSM972379 | Y | 0 | 95 | pMMR | 10.51363286 |
| GSM972380 | Y | 1 | 15 | pMMR | 9.343549385 |
| GSM972381 | Y | 1 | 48 | pMMR | 11.25842192 |
| GSM972382 | N | 1 | 38 | pMMR | 10.35548025 |
| GSM972383 | N | 0 | 92 | dMMR | 10.87925981 |
| GSM972384 | N | 1 | 106 | pMMR | 9.799398146 |
| GSM972385 | N | 0 | 63 | pMMR | 10.54812189 |
| GSM972386 | N | 0 | 90 | pMMR | 9.654725205 |
| GSM972387 | N | 0 | 123 | pMMR | 10.23219218 |
| GSM972388 | N | 1 | 83 | pMMR | 10.12935369 |
| GSM972389 | N | 0 | 93 | pMMR | 9.954715813 |
| GSM972390 | N | 1 | 8 | dMMR | 8.561929107 |
| GSM972391 | N | 1 | 52 | dMMR | 9.528170658 |
| GSM972392 | N | 1 | 44 | pMMR | 10.94678512 |
| GSM972393 | N | 1 | 47 | pMMR | 10.534185 |
| GSM972394 | N | 1 | 11 | pMMR | 9.956641021 |
| GSM972395 | Y | 0 | 147 | pMMR | 9.735905257 |
| GSM972396 | N | 0 | 107 | pMMR | 10.26000095 |
| GSM972397 | N | 0 | 105 | pMMR | 9.612113621 |
| GSM972398 | N | 1 | 64 | pMMR | 10.02161278 |
| GSM972399 | N | 0 | 90 | pMMR | 10.741776 |
| GSM972400 | Y | 1 | 11 | pMMR | 9.963870406 |
| GSM972401 | N | 1 | 0 | pMMR | 8.834327802 |
| GSM972402 | Y | 1 | 31 | N/A | 10.0795195 |
| GSM972403 | N | 0 | 56 | N/A | 9.305537521 |
| GSM972404 | N | 0 | 48 | N/A | 10.33889899 |
| GSM972405 | N | 1 | 79 | N/A | 11.15822013 |
| GSM972406 | N | 0 | 106 | N/A | 10.37953724 |
| GSM972407 | Y | 0 | 132 | N/A | 10.65039424 |
| GSM972408 | Y | 1 | 32 | N/A | 10.9955338 |
| GSM972409 | N | 0 | 104 | N/A | 9.653453733 |
| GSM972410 | N | 0 | 91 | N/A | 10.19668669 |
| GSM972411 | Y | 0 | 125 | pMMR | 10.11254247 |
| GSM972412 | Y | 1 | 8 | pMMR | 10.93043765 |
| GSM972413 | Y | 1 | 20 | pMMR | 9.6401064 |
| GSM972414 | N | 0 | 53 | pMMR | 10.09069928 |
| GSM972415 | Y | 1 | 42 | pMMR | 10.52081532 |
| GSM972416 | Y | 1 | 8 | pMMR | 10.05681658 |
| GSM972417 | Y | 0 | 146 | pMMR | 10.43378531 |
| GSM972418 | N | 0 | 70 | pMMR | 10.75883075 |
| GSM972419 | N | 1 | 32 | pMMR | 10.22894843 |
| GSM972420 | N | 1 | 6 | pMMR | 8.874514305 |
| GSM972421 | N | 0 | 28 | pMMR | 9.93313989 |
| GSM972422 | N | 1 | 37 | dMMR | 9.30547074 |
| GSM972423 | Y | 0 | 40 | pMMR | 9.818841324 |
| GSM972424 | Y | 0 | 104 | pMMR | 10.74049454 |
| GSM972425 | Y | 0 | 59 | pMMR | 9.410007974 |
| GSM972426 | Y | 0 | 81 | pMMR | 9.803198501 |
| GSM972427 | N | 0 | 53 | pMMR | 10.76134241 |
| GSM972428 | Y | 0 | 55 | pMMR | 10.37982084 |
| GSM972429 | Y | 0 | 62 | pMMR | 10.31877744 |
| GSM972430 | Y | 0 | 53 | pMMR | 9.989808416 |
| GSM972431 | Y | 0 | 54 | pMMR | 9.85018651 |
| GSM972432 | Y | 0 | 55 | pMMR | 10.05550833 |
| GSM972433 | N | 1 | 16 | pMMR | 9.816756291 |
| GSM972434 | N | 1 | 42 | pMMR | 10.05428338 |
| GSM972435 | Y | 0 | 40 | pMMR | 10.69265262 |
| GSM972436 | N | 1 | 18 | pMMR | 9.354625491 |
| GSM972437 | N/A | 1 | 7 | pMMR | 10.04857925 |
| GSM972438 | N/A | 1 | 30 | pMMR | 8.32713977 |
| GSM972439 | N/A | 1 | 2 | pMMR | 10.38670108 |
| GSM972440 | Y | 0 | 52 | pMMR | 9.751247659 |
| GSM972441 | N/A | 0 | 68 | pMMR | 9.237575292 |
| GSM972442 | N/A | 1 | 6 | pMMR | 10.61197949 |
| GSM972443 | N | 0 | 48 | dMMR | 10.53969288 |
| GSM972444 | Y | 1 | 14 | pMMR | 8.269650772 |
| GSM972445 | N | 0 | 35 | pMMR | 8.691492364 |
| GSM972446 | N/A | N/A | N/A | dMMR | 8.595495037 |
| GSM972447 | N | 0 | 31 | dMMR | 9.483759475 |
| GSM972447 | N | 0 | 31 | dMMR | 9.483759475 |
| GSM972448 | N | N/A | N/A | pMMR | 6.915149798 |
| GSM972449 | Y | 1 | 41 | pMMR | 10.20795003 |
| GSM972450 | N | 1 | 47 | pMMR | 10.40785608 |
| GSM972451 | N | 0 | 97 | pMMR | 10.65651165 |
| GSM972452 | Y | 0 | 99 | N/A | 9.411971995 |
| GSM972453 | Y | 0 | 100 | pMMR | 10.07555284 |
| GSM972454 | N | 0 | 75 | pMMR | 10.56937325 |
| GSM972455 | N | 0 | 74 | dMMR | 8.651635957 |
| GSM972456 | N | 1 | 0 | pMMR | 9.832862461 |
| GSM972457 | Y | 0 | 68 | pMMR | 10.17470078 |
| GSM972458 | N | 1 | 17 | dMMR | 9.839370726 |
| GSM972459 | N | 0 | 12 | pMMR | 9.109506673 |
| GSM972460 | Y | 0 | 96 | pMMR | 10.39441674 |
| GSM972461 | N | 0 | 96 | pMMR | 10.51625964 |
| GSM972462 | N | 0 | 88 | pMMR | 9.619224084 |
| GSM972463 | N | 1 | 2 | pMMR | 10.5588803 |
| GSM972464 | N | 0 | 86 | pMMR | 9.861187539 |
| GSM972465 | N | 1 | 84 | pMMR | 8.966385886 |
| GSM972466 | Y | 0 | 75 | pMMR | 10.10119075 |
| GSM972467 | Y | 0 | 72 | pMMR | 8.897592525 |
| GSM972468 | N | 1 | 24 | pMMR | 10.58860727 |
| GSM972469 | N | 0 | 86 | pMMR | 10.36115058 |
| GSM972470 | Y | 0 | 85 | dMMR | 9.974251816 |
| GSM972471 | N | N/A | N/A | dMMR | 9.297767731 |
| GSM972472 | Y | 0 | 80 | pMMR | 8.987619442 |
| GSM972473 | N | 0 | 117 | pMMR | 10.6311708 |
| GSM972474 | Y | 0 | 119 | pMMR | 10.52390578 |
| GSM972475 | N | 0 | 87 | pMMR | 9.928510951 |
| GSM972476 | Y | 0 | 64 | pMMR | 10.00335627 |
| GSM972477 | N | 0 | 81 | pMMR | 10.0029345 |
| GSM972478 | N | 0 | 69 | pMMR | 10.32413391 |
| GSM972479 | N | 0 | 3 | pMMR | 6.826736109 |
| GSM972480 | N | 0 | 69 | pMMR | 10.34887136 |
| GSM972481 | Y | 1 | 65 | pMMR | 10.30653564 |
| GSM972482 | N | 0 | 42 | pMMR | 8.982533161 |
| GSM972483 | N | 1 | 28 | pMMR | 9.923233694 |
| GSM972484 | Y | 0 | 76 | pMMR | 10.15746832 |
| GSM972485 | N | 0 | 75 | pMMR | 9.634756544 |
| GSM972486 | N | 0 | 33 | dMMR | 10.82476768 |
| GSM972487 | N | 0 | 67 | N/A | 10.04527728 |
| GSM972488 | N | 0 | 49 | pMMR | 10.03554186 |
| GSM972489 | N | 0 | 74 | pMMR | 10.27256611 |
| GSM972490 | N | 0 | 74 | pMMR | 10.52127537 |
| GSM972491 | N | 0 | 70 | pMMR | 10.1440548 |
| GSM972492 | N | 0 | 68 | pMMR | 10.52761629 |
| GSM972493 | N | 0 | 59 | pMMR | 10.17225448 |
| GSM972494 | N | 0 | 57 | dMMR | 10.20571057 |
| GSM972495 | Y | 1 | 27 | dMMR | 10.61287465 |
| GSM972496 | N | 0 | 142 | pMMR | 10.74245056 |
| GSM972497 | N | 0 | 1 | pMMR | 9.470815456 |
| GSM972498 | N | 1 | 59 | pMMR | 9.570204418 |
| GSM972499 | Y | 0 | 72 | pMMR | 8.604836055 |
| GSM972500 | N | N/A | N/A | pMMR | 7.599335542 |
| GSM972501 | N | 1 | 106 | pMMR | 9.041351075 |
| GSM972502 | N | 0 | 131 | pMMR | 9.933197155 |
| GSM972503 | Y | 0 | 146 | pMMR | 9.577313352 |
| GSM972504 | N | 1 | 30 | pMMR | 9.775028917 |
| GSM972505 | N | 0 | 144 | pMMR | 9.192157694 |
| GSM972506 | N | 0 | 141 | pMMR | 9.713984044 |
| GSM972507 | N | 0 | 42 | dMMR | 9.990300609 |
| GSM972508 | N | 0 | 134 | pMMR | 10.36855603 |
| GSM972509 | N | 0 | 14 | dMMR | 10.72768341 |
| GSM972510 | N | 0 | 86 | pMMR | 10.80806759 |
| GSM972511 | Y | 1 | 47 | pMMR | 9.632268954 |
| GSM972512 | Y | 1 | 23 | pMMR | 10.19649104 |
| GSM972513 | N | 1 | 48 | pMMR | 10.92849506 |
| GSM972514 | Y | 1 | 32 | pMMR | 9.911563371 |
| GSM972515 | N | 0 | 118 | pMMR | 10.67274313 |
| GSM972516 | N | 0 | 66 | pMMR | 10.20195338 |
| GSM972517 | N | 1 | 37 | pMMR | 10.34838305 |
| GSM972518 | N | 1 | 35 | pMMR | 9.97766473 |
| GSM972519 | N | 1 | 14 | pMMR | 9.957854715 |
| GSM972520 | Y | 1 | 62 | pMMR | 9.569501695 |
| GSM972521 | N | 1 | 76 | pMMR | 9.791915115 |
| GSM972522 | Y | 0 | 99 | pMMR | 9.362305891 |

**Table S5**. Patient characteristics and ACSL5 expression determined by immunohistochemistry in primary colorectal cancers tumors in a 90-patient TMA-based cohort (related to Figure 9E, F).

| Location | ACSL5 expression | death-yes 1，no 0 | survival time (months) |
| --- | --- | --- | --- |
| D5 | ++++ | 1 | 99 |
| G11 | ++++ | 0 | 99 |
| G13 | +++ | 0 | 99 |
| H9 | ++ | 0 | 99 |
| H7 | ++ | 0 | 99 |
| H3 | + | 0 | 99 |
| A7 | +++ | 1 | 98 |
| H17 | +++ | 0 | 98 |
| H13 | +++ | 0 | 98 |
| I5 | + | 0 | 98 |
| H15 | + | 0 | 98 |
| I17 | ++++ | 0 | 97 |
| H1 | +++ | 1 | 97 |
| I11 | ++ | 0 | 97 |
| J5 | ++ | 0 | 97 |
| I7 | + | 0 | 97 |
| J7 | + | 0 | 96 |
| J15 | ++ | 0 | 95 |
| J17 | + | 0 | 95 |
| J13 | + | 0 | 95 |
| B11 | + | 1 | 89 |
| E15 | +++ | 0 | 85 |
| G15 | ++ | 1 | 68 |
| C9 | ++ | 0 | 67 |
| I3 | ++ | 0 | 67 |
| F9 | +++ | 0 | 54 |
| A1 | ++++ | 0 | 108 |
| A13 | ++++ | 0 | 107 |
| A9 | + | 0 | 107 |
| B5 | ++ | 0 | 106 |
| B13 | +++ | 0 | 105 |
| C1 | + | 0 | 105 |
| C11 | ++++ | 0 | 104 |
| C15 | ++++ | 0 | 104 |
| C7 | +++ | 0 | 104 |
| C3 | +++ | 0 | 104 |
| C17 | ++ | 0 | 104 |
| D1 | ++ | 0 | 104 |
| D3 | ++++ | 0 | 103 |
| B7 | +++ | 1 | 103 |
| D9 | +++ | 0 | 103 |
| D11 | ++ | 0 | 103 |
| D15 | + | 0 | 102 |
| F11 | +++ | 1 | 101 |
| E5 | +++ | 0 | 101 |
| E7 | ++ | 0 | 101 |
| E3 | + | 0 | 101 |
| E11 | + | 0 | 101 |
| F15 | ++ | 0 | 100 |
| F3 | ++ | 0 | 100 |
| F5 | + | 0 | 100 |
| E17 | + | 0 | 100 |
| B3 | +++ | 1 | 66 |
| G1 | + | 1 | 66 |
| D17 | + | 1 | 64 |
| G7 | + | 1 | 52 |
| G5 | + | 1 | 52 |
| I9 | +++ | 1 | 48 |
| J11 | + | 1 | 48 |
| E9 | ++ | 1 | 47 |
| A15 | + | 1 | 47 |
| F13 | + | 1 | 45 |
| E1 | +++ | 1 | 43 |
| F7 | ++ | 1 | 43 |
| F1 | + | 1 | 42 |
| G3 | +++ | 1 | 36 |
| I13 | + | 1 | 35 |
| A3 | + | 1 | 34 |
| D7 | ++++ | 1 | 30 |
| D13 | ++++ | 1 | 28 |
| G9 | ++ | 1 | 27 |
| I15 | + | 1 | 26 |
| A11 | + | 1 | 24 |
| G17 | + | 1 | 24 |
| H11 | + | 1 | 24 |
| A5 | + | 1 | 23 |
| A17 | + | 1 | 23 |
| B1 | ++ | 1 | 19 |
| B9 | + | 1 | 18 |
| J3 | ++ | 1 | 17 |
| C5 | ++ | 1 | 16 |
| F17 | + | 1 | 16 |
| B17 | + | 1 | 14 |
| C13 | + | 1 | 13 |
| J9 | + | 1 | 9 |
| J1 | + | 1 | 7 |
| H5 | + | 1 | 6 |
| B15 | + | 1 | 3 |
| E13 | ++++ | 1 | 1 |
| I1 | + | 1 | 1 |

**Table S6**. Cell line sources and culture media.

| Cell line | RRIDs | source | Culture medium |
| --- | --- | --- | --- |
| HEK293 | CVCL_0063 | Anwei-sci Cell Center，Shanghai，China, Cell NO. AW-CH0004 | DMEM (GIBCO:10566016) |
| HCT116 | CVCL_0291 | Anwei-sci Cell Center，Shanghai，China, Cell NO. AW-CH0109 | DMEM (GIBCO:10566016) |
| HepG2 | CVCL_0027 | Anwei-sci Cell Center，Shanghai，China, Cell NO. AW-CH0092 | MEM (GIBCO:11095080) |
| HT29 | CVCL_0320 | Anwei-sci Cell Center，Shanghai，China, Cell NO.AW-CH0157 | RPMI 1640 (GIBCO:11875093) |
| RKO | CVCL_0504 | Anwei-sci Cell Center，Shanghai，China, Cell NO.AW-CH0299 | MEM (GIBCO:11095080) |
| 786-O | CVCL_1051 | From Henan Provincial Key Laboratory of Kidney Disease and Immunology, Henan Provincial People’s Hospital, a donation from Professor Yanliang Wang | RPMI 1640 (GIBCO:11875093) |
| KYSE450 | CVCL_1353 | From Translational Research Institute, Henan Provincial People’s Hospital, a donation from Professor Xiaoying Liu | RPMI 1640 (GIBCO:11875093) |
| A549 | CVCL_0023 | From Division of Life Sciences and Medicine, University of  Science and Technology of China, a donation from Professor Mian Wu | F-12K (Solarbio:LA1320) |

**Table S7.** shRNA and PCR primer sequences.

|  | Primer name | Primer Sequence |
| --- | --- | --- |
| shRNA | sh-ACSL5-1 | GCAAGAAAGCTAACACTTA |
| shRNA | sh-ACSL5-2 | ACAAACGTGTTCAAAGGAT |
| shRNA | sh-NF-κB | ACGTTCCTATTGTCATTAA |
| shRNA | sh-SP1 | CAGCTTCAGGCTGTTCCAA |
| shRNA | sh-HSF1 | CAGCTCCTTGAGAACATCA |
| shRNA | sh-p53 | AGCAGCAACCCAGACTATA |
| shRNA | sh-STAT3 | ACCAACGACCTGCAGCAAT |
| shRNA | sh-MDM2 | GTGCCAAGCTTCTCTGTGA |
| shRNA | sh-TOM20 | TACAGCTTGGTGAAGAGTT |
| shRNA | sh-TOM70 | AGGAAAATATGAACAAGCT |
| shRNA | sh-TOM40 | CCATGCAAGTTACTACCAC |
| siRNA | si-MIB1-1 | CCGGAAUAACCGGGUGAUGG |
| siRNA | si-MIB1-2 | CACUUCCCGGUGUAGUAAUU |
| siRNA | si-PGAM1 | CCCUUCUGGAAUGAAGAAAUA |
| PCR | pCDH-ACSL5-F | CGGAATTCATGGACGCTCTGAAGCCACC |
| PCR | pCDH-ACSL5-R | GGACTAGTTCCTGGATGTGCTCATACAG |
| PCR | pSIN-3xflag-ACSL5-F | GAATTCATGGACGCTCTGAAGCCACCCT |
| PCR | pSIN-3xflag-ACSL5-R | ACTAGTTGTATGAGCACATCCAGGATTAG |
| PCR | pLVX-ACSL5-F | CTCGTAAAGAATTCATGGACGCTCTGAAG |
| PCR | pLVX-ACSL5-R | TTTGTAGTCGGATCCATCCTGGATGTGCTC |
| PCR | pCDNA3.1-3xflag-ACSL5-F | GGATCCCGAATTCTAATGGACGCTCTGAAGCCACCC |
| PCR | pCDNA3.1-3xflag-ACSL5-R | CCCTCTAGACTCGAGATCCTGGATGTGCTCATACA |
| PCR | pCDNA3.1-3xflag-ACSL5-TM-F | TCGGATCCCGAATTCATTCCCCACTTCCGACCCC |
| PCR | pCDNA3.1-3xflag-ACSL5-CNM1/AMP/CNM2-F | TCGGATCCCGAATTCTAACCAGACCTCAACCCGTC |
| PCR | pCDNA3.1-3xflag-ACSL5-TM-R | CCCTCTAGACTCGAGGATCAGCCACAAGAAGATGGCA |
| PCR | pCDNA3.1-3xflag-ACSL5-AMP-F | TCGGATCCCGAATTCTACAGCCCTACAGATGGCTATC |
| PCR | pCDNA3.1-3xflag-ACSL5-CNM1-R | GGGCCCTCTAGACTCGAGGTTTGGTTTTCTATATC |
| PCR | pCDNA3.1-3xflag-ACSL5-CNM2-F | TCGGATCCCGAATTCTAAAGGACCCTGAGAAG |
| PCR | pCDNA3.1-3xflag-ACSL5-AMP-R | GCCCTCTAGACTCGAGCAGGTATCCTTTGAACACG |
| PCR | pGL3-P1-F | GCTAGTCTCTCCTCCACACA |
| PCR | pGL3-P1-R | AGGCTCAAGCATGGCAGGCT |
| PCR | pGL3-P2/P3-F | CTTTATGTTTTTGGCGTCTTCCA |
| PCR | pGL3-P2/P3-R | ACCATGTTGGCCAGGCTGGT |
| PCR | pGL3-P4-F | CTCATGACTCTGAGTCAGGTGC |
| PCR | pGL3-P4-R | ACTGGCAGGCAGCTTCACCT |
| PCR | pCDNA3.1-HA-MIB1-F | TGGCTAGTTAAGCTTGGATCCATGTACCCATACGA |
| PCR | pCDNA3.1-HA-MIB1-R | GAATTCTTAATACAAAAGAATCCTTCGTTCAATAGC |
| PCR | pCDNA3.1-HA-MIB1(1-429)-F | TGGCTAGTTAAGCTTGGATCCATGTACCCATACGA |
| PCR | pCDNA3.1-HA-MIB1(1-429)-R | CCGCGGTACCTCGAGGTCACCAGATTCTTG |
| PCR | pCDNA3.1-HA-MIB1(430-729)-F | TGGAGGCCCGAATTCTATATGACCTCAATGAAGAAT |
| PCR | pCDNA3.1-HA-MIB1(430-729)-R | CCGCGGTACCTCGAGAGCATCCACCTTCCCC |
| PCR | pCDNA3.1-HA-MIB1(430-1006)-F | TGGAGGCCCGAATTCTATATGACCTCAATGAAGAAT |
| PCR | pCDNA3.1-HA-MIB1(430-1006)-R | GAATTCTTAATACAAAAGAATCCTTCGTTCAATAGC |
| PCR | pCDNA3.1-HA-MIB1-RING-F | TGGAGGCCCGAATTCTATATGCCTGGGAGCCATCC |
| PCR | pCDNA3.1-HA-MIB1-RING-R | GAATTCTTAATACAAAAGAATCCTTCGTTCAATAGC |
| PCR | pCDNA3.1-HA-MIB1-∆RING-F | TGGCTAGTTAAGCTTGGATCCATGTACCCATACGA |
| PCR | pCDNA3.1-HA-MIB1-∆RING-R | CCGCGGTACCTCGAGAGCATCCACCTTCCCC |
| PCR | pEGFP-IDH2-F | GACGAGCTGTACAAGATGTCCAAAAAAATC |
| PCR | pEGFP-IDH2-R | TCTAGATCCGGTGGATTAAAGTTTGGCCTG |
| PCR | pCMV-HA-IDH2-F | CCCGAATTCATGGCCGGCTACCTGCGGGT |
| PCR | pCMV-HA-IDH2-R | GGTACCTCGAGCTACTGCCTGCCCAGGGCTC |
| PCR | pCMV-HA-IDH2-MTS-F | TGGAGGCCCGAATTCATGGCCGGCTACCTGCGGGT |
| PCR | pCMV-HA-IDH2-MTS-R | CCGCGGCCGCGGTACCTCGAGATAGTGGCGCCGCG |
| PCR | pCMV-HA-IDH2-∆MTS-F | TGGAGGCCCGAATTCGCCGACAAAAGGATC |
| PCR | pCMV-HA-IDH2-∆MTS-R | CCGCGGCCGCGGTACCTCGAGCTACTGCCTGCCCAG |
| PCR | pCMV-HA-IDH2-P2/P3-F | TGGAGGCCCGAATTCAACTATGACGGAGAT |
| PCR | pCMV-HA-IDH2-MTS/P1-R | CCGCGGTACCTCGAGGTTCTTGCAGGCCCACACAA |
| PCR | pCMV-HA-IDH2-P3-F | TGGAGGCCCGAATTCTAATGACGTCCGTCCTGGTCTG |
| PCR | pCMV-HA-IDH2-∆P3-R | CCGCGGTACCTCGAGCATCAGGCCAAGGGAGCCAA |
| PCR | F1（for ACSL5 knockout mice） | AAGTCCCAGCCCCTTCTACC |
| PCR | R1（for ACSL5 knockout mice） | GGGATTATGTGCTCAGAGAAAGACA |
| PCR | F2（for ACSL5 knockout mice） | GCTCTAGTCTATGTGGTTGGACTT |
| PCR | R2（for ACSL5 knockout mice） | CTACTGTGGGCACCTTAATCTCAA |

**Table S8.** qRT-PCR primer sequences.

|  | Name | Primer Sequence 5’-3’ |
| --- | --- | --- |
| qPCR-PCR | qPCR-ACSL5-F | GGCCAAACAGAATGCACAG |
| qPCR-PCR | qPCR-ACSL5-R | GGAGTCCCAACATGACCTG |
| qPCR-PCR | qPCR-p53-F | AGAGTCTATAGGCCCACCCC |
| qPCR-PCR | qPCR-P53-R | GCTCGACGCTAGGATCTGAC |
| qPCR-PCR | qPCR-MDM2-F | ATCCGGATCTTGATGCTGGT |
| qPCR-PCR | qPCR-MDM2-R | CTCTCCCCTGCCTGATACAC |
| qPCR-PCR | qPCR-IDH2-F | ATCTCAGGTTTTGCGCACAG |
| qPCR-PCR | qPCR-IDH2-R | AAGACTTGAGGACCTGAGCC |
| qPCR-PCR | qPCR-GAPDH-F | GCACCGTCAAGGCTGAGAAC |
| qPCR-PCR | qPCR-GAPDH-R | TGGTGAAGACGCCAGTGGA |
| qPCR-PCR | qPCR-p21-F（for Chip） | CTGTCCTCCCCGAGGTCA |
| qPCR-PCR | qPCR-p21-R（for Chip） | ACATCTCAGGCTGCTCAGAGTCT |
| qPCR-PCR | qPCR-GAPDH-F（for Chip） | TACTAGCGGTTTTACGGGCG |
| qPCR-PCR | qPCR-GAPDH-R（for Chip） | TCGAACAGGAGGAGCAGAGAGCGA |
| qPCR-PCR | qPCR-ACSL5-p1-F（for Chip） | CTGGCTGAGGCTGGAGCC |
| qPCR-PCR | qPCR-ACSL5-p1-R（for Chip） | CGGGGCTGTACGCGGGG |
| qPCR-PCR | qPCR-ACSL5-p2/p3-F（for Chip） | CTGGCTGAGGCTGGAGCC |
| qPCR-PCR | qPCR-ACSL5-p2/p3-R（for Chip） | CTTAACAAGTGGTACGCCACC |
| qPCR-PCR | qPCR-ACSL5-p4-F（for Chip） | TTAGCACCCCGGCCAGCG |
| qPCR-PCR | qPCR-ACSL5-p4-R（for Chip） | CAGGAGCCCACGGAGGGTGG |
